# Synthesis and biophysical properties of carbamate-locked nucleic acid (LNA) oligonucleotides with potential antisense applications†
†Electronic supplementary information (ESI) available. See DOI: 10.1039/c9ob00691e


**DOI:** 10.1039/c9ob00691e

**Published:** 2019-05-17

**Authors:** Cameron Thorpe, Sven Epple, Benjamin Woods, Afaf H. El-Sagheer, Tom Brown

**Affiliations:** a Department of Chemistry , University of Oxford , 12 Mansfield Road , Oxford OX1 3TA , UK . Email: tom.brown@chem.ox.ac.uk; b Chemistry Branch , Department of Science and Mathematics , Faculty of Petroleum and Mining Engineering , Suez University , Suez 43721 , Egypt

## Abstract

Antisense oligonucleotides (ASOs) are becoming important drugs for hard to treat diseases. Modifications to their DNA backbones are essential to inhibit degradation *in vivo*, but they can reduce binding affinity to RNA targets. To address this problem we have combined the enzymatic resistance of carbamate (CBM) DNA backbone analogues with the thermodynamic stability conferred by locked nucleic acid sugars (LNA). Using a dinucleotide phosphoramidite strategy and automated solid phase synthesis, we have synthesised a set of oligonucleotides modified with multiple LNA-CBM units. The LNA sugars restore binding affinity to RNA targets, and in this respect LNA position with respect to the CBM linkage is important. Oligonucleotides containing carbamate flanked on its 5′and 3′-sides by LNA form stable duplexes with RNA and unstable duplexes with DNA, which is desirable for antisense applications. Carbamate-LNA modified oligonucleotides also show increased stability in the presence of snake venom and foetal bovine serum compared to LNA or CBM backbones alone.

## Abstract 2

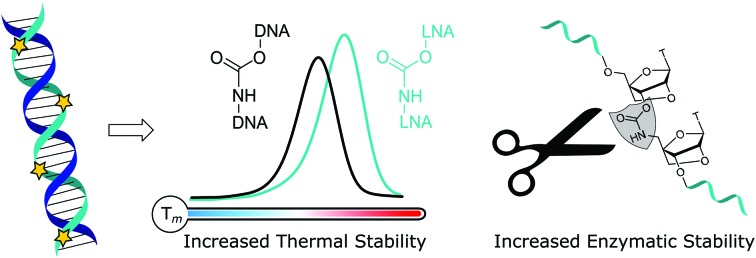
Carbamate-LNA oligonucleotides have improved biophysical properties for theraputic applications.

## Introduction

Antisense oligonucleotides (ASOs) are short ∼20 mer sequences that bind to complementary ribonucleic nucleic acid (RNA) targets through Watson–Crick base pairing to promote enzymatic degradation (RNase H), induce RNA interference (RNAi) or regulate protein expression (splice modulation).^1^ Enabled by target specific binding, there are now several FDA approved ASO-based drugs in the clinic treating a range of genetic and hereditary diseases.^2^ Eteplirsen (Exondys 51)^3^ and Nusinersen (Spinraza)^4^ have been developed for the treatment of Duchenne muscular dystrophy and spinal muscular atrophy respectively. Antisense therapeutics are also making headway against severe hypercholesterolemia following the release of Mipomersen.^5^ Furthermore, with the recent approval in 2018 of Patisiran^6^ and Inotersen^7^ treating fatal hereditary transthyretin-mediated amyloidosis, the ASO field demonstrates its versatility for treatment of diseases that could be described as “undruggable” by small molecule approaches. Unfortunately, due to poor cell penetration^8^ combined with fast cellular degradation, unmodified sequences are less than ideal candidates for therapeutic applications. To overcome this, much research has focused on the incorporation of backbone modifications^9^,^10^ into ASOs. In regard to degradation of the phosphodiester backbone of ASOs by nuclease enzymes, chemical modification of sugar-phosphate backbones can significantly improve biological stability. Several classes of antisense modifications have emerged, and have been used in FDA approved ASOs. Common modifications include 2′-*O*-methoxy or 2′-*O*-(2-methoxyethyl) (MOE) ribose sugars, backbone modifications such as phosphorothioates^11^ (Mipomersen and Nusinersen) or phosphorodithioates and sugar alternatives such as morpholino (Eteplirsen) or threose nucleic acid (TNA).^12^,^13^ Amide^14^–^18^ and triazole^19^–^21^ artificial DNA backbones also have potential, but are yet to reach maturity in the antisense field. Further improvements to the design of chemically modified oligonucleotides (ONs), specifically those that increase hybridisation strength (RNA-target affinity) and stability *in vivo* are sought after, particularly if they also influence other properties such as cellular uptake.^22^

**Fig. 1:**
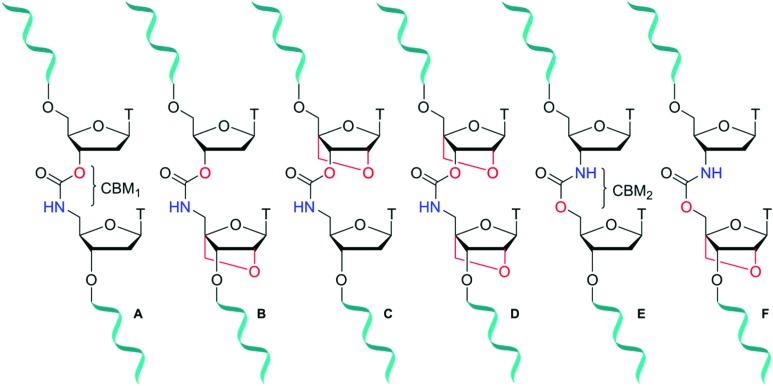
Carbamate-modified DNA backbones containing LNA sugars. Set 1 (A–D) and set 2 (E, F) comprise of dimers with the constitutional carbamate isomers CBM_1_ and CBM_2_ respectively. A = DNA-CBM_1_-DNA, B = DNA-CBM_1_-LNA, C = LNA-CBM_1_-DNA, D = LNA-CBM_1_-LNA, E = DNA-CBM_2_-DNA, F = DNA-CBM_2_-LNA.

Locked nucleic acid (LNA), also known as bridged nucleic acid (BNA), has been a transformative development in the nucleic acid field. LNA is a bicyclic ribose analogue with a 2′-*O*-4′-methylene sugar bridge. The C3′-*endo* conformation is preferred and consequently LNA displays unparalleled binding affinity to complementary RNA strands; LNA modifications can stabilise duplexes by up to +7 °C per modification.^23^,^24^ Building on the seminal work of Wengel^23^,^25^ and Obika,^26^,^27^ LNA has been used in a variety of applications including siRNA-mediated gene silencing, CRISPR-Cas9,^28^ and triplex formation.^29^,^30^ A combination of LNA with a DNA backbone mimic comprised of a six-atom triazole linkage improves duplex stability compared to the unmodified counterpart.^20^ In contrast, Watts *et al.*^31^ found that addition of LNA to a four-atom triazole linkage caused duplex destabilisation. This indicates that the contributions of linkage length and flexibility of DNA backbone analogues when combined with LNA as modulators of duplex stability is still poorly understood. It is thus important to expand the range of artificial backbones-LNA combinations beyond the triazole and amide systems.^32^,^33^ The carbamate backbone is a four-atom, flexible, charge-neutral linkage with two constitutional isomers CBM_1_ (Fig. 1A) and CBM_2_ (Fig. 1E). Carbamate backbone incorporation into DNA has previously been reported by Waldner and De Mesmaeker *et al.*^34^ They found that duplex destabilisation occurs, but good stability against enzymatic degradation is achieved. Hence, in isolation carbamates are less than ideal candidates for antisense applications because of reduced binding affinity. In pursuit of new modified oligonucleotide constructs, we sought to combine the enzymatic stability of carbamate backbones with the favourable thermodynamic properties of LNA. We now report the synthesis of a set of dinucleotide phosphoramidites designed to introduce the carbamate-LNA modifications in Fig. 1 into oligonucleotides sequences using standard solid phase synthesis. The modified oligomers were synthesised and evaluated by ultraviolet melting (hybridisation) studies in order to determine the most favourable carbamate-LNA combinations as modified DNA backbones. Enzymatic digestion assays were carried out using snake venom and foetal bovine serum.

## Results and discussion

### Synthesis of LNA-CBM_1_ dinucleotides

**Scheme 1:**
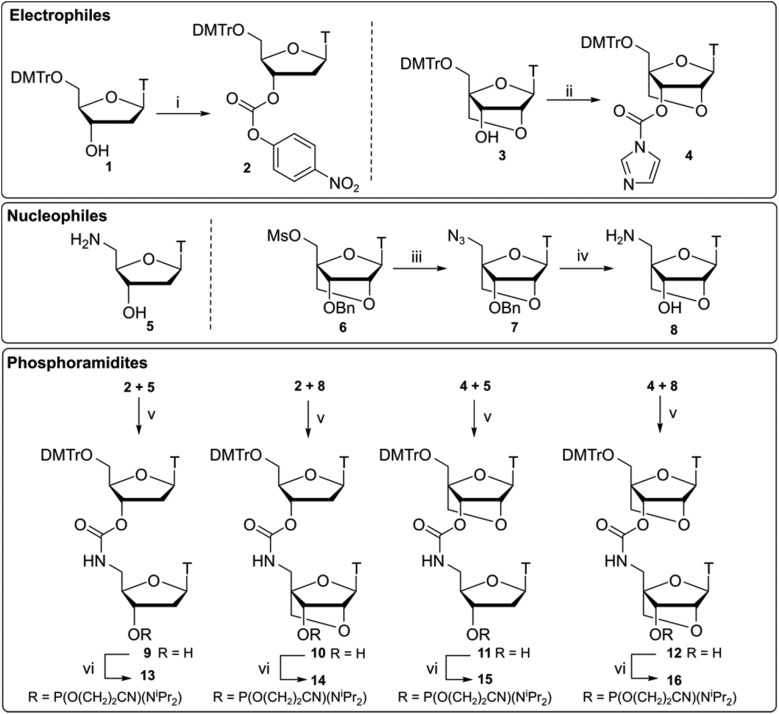
Synthesis of CBM_1_-linked dinucleotides **13–16** using different combinations of 3′-activated carbonates **2**, **4** and 5′-amines **5**, **8**. Reagents and conditions (i) *p*-nitrophenyl chloroformate, pyridine, 80 °C, 20 h, crude **2** 60%; (ii) CDI, THF, rt, 20 h, crude **4** 84%; (iii) NaN_3_, DMF, 65 °C, 20 h, **7** 90%; (iv) H_2_, Pd(OH)_2_ (20%)/C MeOH, rt, 3 h; NH_4_HCO_2_, reflux, 4 h, **8** 87%; (v) DMAP, pyridine, 80 °C, 20 h, **9** 62%, **10** 51%, **11** 63%, **12** 70%; (vi) Chloro(diisopropylamino)-β-cyanoethoxyphosphine, DIPEA, CH_2_Cl_2_, rt, 1 h, **13** 54%, **14** 57%, **15** 36%, **16** 88%.

Compounds **13–16** (Scheme 1) were synthesised by ligation of 3′-activated carbonyls **2** and **4** with 5′-nucleophilic amines **5** and **8** following a previously reported synthesis by Waldner *et al.*^34^ Starting material **1** was reacted with *p*-nitrophenyl (PNP) chloroformate to give the activated nucleoside **2**. Subsequent reaction with amine **5** under basic conditions gave dinucleotide **9** which was converted to phosphoramidite **13** using standard phosphitylating conditions. For the LNA monomers, a divergent synthetic strategy was used. Compounds **3** and **6** were synthesised^35^ and compound **3** was converted to an activated imidazole carbamate **4**. Unlike DNA analogue **2**, activation of the 3′-OH by *p*-nitrophenyl (PNP) chloroformate was slow and resulted in poor yields. It has been previously suggested that PNP carbonates could be susceptible to degradation in basic solvent,^36^ preventing efficient activation of the 3′-position. To overcome this, we opted for a one pot strategy directly reacting alcohol **3** with CDI to form imidazole carbamate **4** which was then used without further purification.

Following work by Obika,^37^ LNA nucleoside **6** was reacted with sodium azide to form its 5′-azide **7**. Reduction of the azido group was performed as a one pot procedure by palladium-catalysed hydrogenation under H_2_ gas. Subsequent deprotection of the 3′-benzyl group was then facilitated by the addition of NH_4_HCO_2_ to give amino nucleoside **8**. Combinations of the required DNA and LNA monomers **2**, **4**, **5**, **8** were then used to give CBM_1_-linked dinucleotides analogues **10–12**. As to be expected, coupling of the more sterically hindered 3′-activated LNA monomer **4** was found to be slower but proceeded with comparable yields to the DNA analogues. These compounds were then converted to their respective phosphoramidites **14–16**.

### Synthesis of LNA-CBM_2_ dinucleotide analogues

**Scheme 2:**
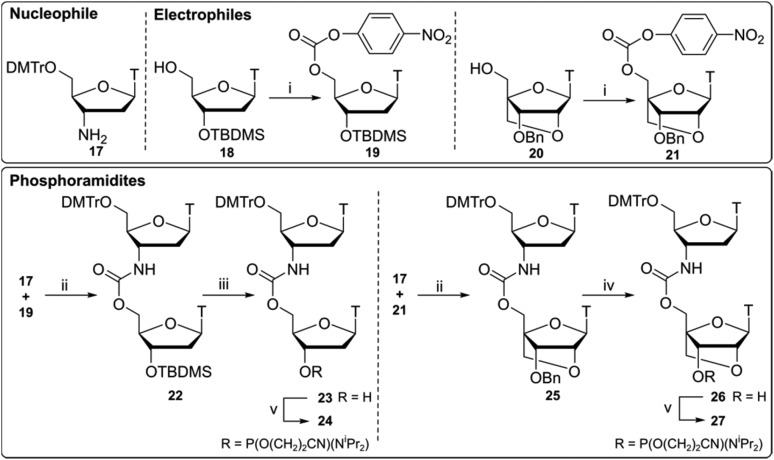
Synthesis of CBM_2_ dinucleotides **24**, **27** using activated 3′-amine **17** and 5′-activated carbonates **19**, **21**. Reagents and conditions (i) *p*-nitrophenyl chloroformate, pyridine, 80 °C, 20 h, **19** 76%, **21** crude 73%, (ii) HOBt, pyridine, 80 °C, 20 h, **22** 67%, **25** 63%; (iii) TBAF, THF, 0 °C, 2 h, **23** 77%; (iv) Pd(OH)_2_ (20%)/C, NH_4_HCO_2_, 65 °C, 2 h, **26** 97%; (v) Chloro(diisopropylamino)-β-cyanoethoxyphosphine, DIPEA, CH_2_Cl_2_, rt, 1 h, **24** 80%, **27** 43%.

Compounds **24** and **27** (Scheme 2) were synthesised using a 3′-nucleophilic amine and activated 5′-carbonate (reverse connectivity to CBM_1_). Compound **17** was obtained by reduction of commercially available 3′-azidothymidine (AZT) over palladium on activated charcoal in good yield. In order to synthesise compound **19** we activated the 5′-OH of unprotected thymidine but this led to an intramolecular reaction *via* 3′-OH nucleophilic attack on the PNP carbonate. Thus, a 5′/3′ protection strategy was employed similar to that of Utagawa *et al.*,^38^ where 5′-*O*-DMTr/3′-*O*-TBDMS protected thymidine was used in the generation of the 5′-activated PNP carbonate (compound **19**) *via* compound **18**. Compound **22** was formed under basic conditions and 3′-*O*-TBDMS protection was removed to form alcohol **23**. This was subsequently phosphitylated to give the phosphoramidite DNA–DNA dimer **24**.

Following a protocol from Koshkin *et al.*,^35^ 5′-activated **21** (Scheme 2) was synthesised. Dimer **25** was formed by coupling of amine **17** with LNA monomer **21** and the 3′-benzyl ether was deprotected by reduction over a Pd(OH)_2_-catalyst to yield alcohol **26** followed by conversion to the phosphoramidite DNA-LNA building block **27**. Previous DNA carbamate duplex melting experiments^34^ indicate that the CBM_2_ linkage is more destabilising than CBM_1_ 22 and recent biophysical studies with LNA-triazole backbones also showed that 5′-LNA adjacent to the modified linkage is highly destabilising in both DNA and RNA duplexes.^20^ Based on these observations we did not pursue the synthesis of this 5′-LNA carbamate monomer and subsequent 5′-LNA carbamate dimers.^39^–^41^

### Duplex stability (hybridisation) studies

#### Comparison of carbamate backbones

Oligonucleotides ON2–13 (Tables 1 and 2), with either single or triple incorporations of dimer phosphoramidites **13–16**, **24** and **27** were prepared by standard solid phase synthesis for thermal duplex stability analyses by UV-melting. No issues with aqueous solubility were observed, and as expected introduction of carbamate backbones between DNA sugars reduced duplex stability when the oligonucleotide was hybridised to either DNA or RNA targets (Table 1, ON2 and ON6). Comparing the two carbamate backbones without LNA modification we confirmed previous reports^34^ that the CBM_1_-linkage causes less duplex disruption against both DNA and RNA targets. In addition, we observed that both CBM_1_ and CBM_2_ isomers were better accommodated within DNA : DNA duplexes than within DNA : RNA hybrids, causing smaller reductions in melting temperature (additional data, Fig. S1 and S2†).

**Table 1:** Melting temperatures (*T*_m_) from single incorporation of backbone modified LNA at central position. x = CBM_1_, y = CBM_2_, T^L^ = LNA thymidine. Values were obtained from the maxima d*A*_260_*vs*. *t* for 3 mM of each oligonucleotide in 10 mM phosphate, 200 mM NaCl buffer, pH 7.0. Δ*T*_m_ are relative to the unmodified control ON1. Addition of LNA on the 5′ site caused large reduction in *T*_m_ against both targets whereas 3′ addition to CBM_1_ linkages gave the most stable modified DNA duplex and 5′/3′ was the most stable against RNA

ON	ON sequence (5′-3′)	DNA target		RNA target	
		*T*_m_	Δ*T*_m_	*T*_m_	Δ*T*_m_
ON1	GCTTGCTTCGTTCC	60.2	—	63.6	—
ON2	GCTTGCT**x**TCGTTCC	57.5	–2.7	57.2	–6.4
ON3	GCTTGCT^L^**x**TCGTTCC	50.5	–9.7	56.1	–7.5
ON4	GCTTGCT**x**T^L^CGTTCC	60.3	0.1	61.3	–2.3
ON5	GCTTGCT^L^**x**T^L^CGTTCC	53.4	–6.8	62.1	–1.5
ON6	GCTTGCT**y**TCGTTCC	53.6	–6.6	55.0	–8.6
ON7	GCTTGCT**y**T^L^CGTTCC	51.7	–8.5	57.1	–6.5

**Table 2:** Melting temperatures (*T*_m_) from triple incorporation of backbone modified LNA at central and terminal positions. x = CBM_1_, y = CBM_2_, T^L^ = LNA thymidine. Values were obtained from the maxima d*A*_260_*vs*. *t* of 3 mM of each oligonucleotide in 10 mM phosphate, 200 mM NaCl buffer, pH 7.0. Δ*T*_m_ are relative to the unmodified control ON1. Incorporation of multiple CBM-LNA modifications was approximately additive compared to single modifications

ON	ON sequence (5′-3′)	DNA target		RNA target	
		*T*_m_	Δ*T*_m_	*T*_m_	Δ*T*_m_
ON1	GCTTGCTTCGTTCC	60.2	—	63.6	—
ON8	GCT**x**TGCT**x**TCGT**x**TCC	52.1	–8.1	44.8	–18.8
ON9	GCT^L^**x**TGCT^L^**x**TCGT^L^**x**TCC	<30	<–30.2	40.6	–23.0
ON10	GCT**x**T^L^GCT**x**T^L^CGT**x**T^L^CC	60.1	–0.1	59.6	–4.0
ON11	GCT^L^**x**T^L^GCT^L^**x**T^L^CGT^L^**x**T^L^CC	41.2	–19.0	61.6	–2.0
ON12	GCT**y**TGCT**y**TCGT**y**TCC	37.9	–22.3	36.5	–27.1
ON13	GCT**y**T^L^GCT**y**T^L^CGT**y**T^L^CC	34.7	–25.5	46.8	–16.8

**Fig. 2:**
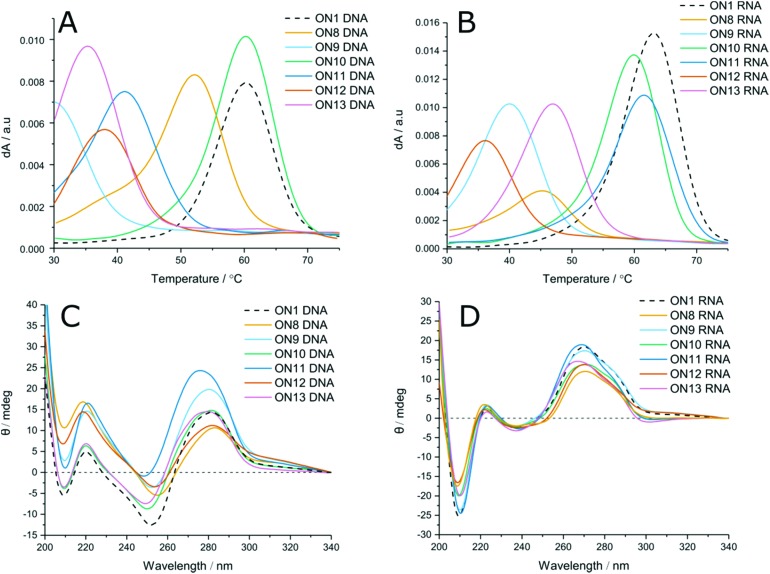
(Top) UV-melting data, 3 μM of oligonucleotides were added to 10 mM phosphate, 200 mM NaCl buffer, pH 7.0 and heated from 20–85 °C. *T*_m_ values were determined from an average of six ramps from a smoothed plot of d*A*/*t*. (A) First derivative *T*_m_ curves against DNA targets containing triple modifications of CBM-LNA, (B) First derivative *T*_m_ curves against RNA targets containing triple modifications of CBM-LNA. (Bottom) Circular dichroism spectra of 3 μM oligonucleotides in 10 mM phosphate, 200 nM NaCl buffer, pH 7.0. Spectra were averaged from four scans and smoothed to 20 points using a third order polynomial. (C) Triple incorporation of CBM-LNA modifications against DNA targets, (D) Triple incorporation of CBM-LNA modifications against RNA targets. Overall duplex conformation against both targets remained largely unchanged. Against DNA targets hypsochromic shifts were observed with addition of LNA.

LNA has been shown to increase duplex stability by up to +7 °C per modification in unmodified phosphodiester duplexes.^42^ To evaluate the positional effects of LNA on carbamate modified duplexes, LNA-containing dinucleotides **14–16** were used to incorporate LNA sugars adjacent to the modified linkages. Unlike with phosphodiester backbones, the presence of 5′-LNA (Fig. 2A and B, ON9) caused further destabilisation to the duplexes compared to the CBM_1_ backbone with unmodified sugars (ON8). Introduction of an LNA modification to the 3′ side (Table 1 ON4, Table 2 ON10, Fig. 2A and B, ON10) had the inverse effect, stabilising duplexes against both DNA and RNA targets by +2.6 and +4.1 °C (ON2 *vs*. ON4) per modification respectively. Addition of LNA to both sides of the linkage against DNA targets resulted in moderate destabilisation, seemingly combining the two effects of 3′ and 5′ LNA (Fig. 2A, ON11). In contrast, LNA addition to both sides of the carbamate linkage against RNA targets (Fig. 2B, ON11) resulted in the most stable carbamate duplexes, outperforming the 3′ addition alone. This is likely to be a consequence of the conformational influence of the LNA sugar. In unmodified DNA duplexes the deoxyribose sugar is C2′-*endo*, leading to a B-form helix. Conversely, LNA prefers a C3′-*endo* conformation characteristic of A-form RNA. Hence, as the LNA content of the DNA strand increases, it can more efficiently hybridise with RNA. As a result, increased LNA content improves hybridisation of modified DNA to complementary RNA sequences. These results also demonstrate that the LNA sugar influences the conformation of the backbone linkage directly on its 3′-side, as observed by Petersen *et al.*,^43^ even if this is not a canonical phosphodiester. However, its influence on a phosphodiester is much greater than on the carbamate analogue. The closer an analogue is to a pure phosphodiester, the greater will be the positive influence of LNA. For example, ON4 and ON10 display high levels of stability because influence of LNA is directed to the phosphodiester linkage at its 3′-side, as in normal LNA : RNA duplexes. When 5′-LNA modifications are introduced (*e.g.* in ON9), LNA-induced conformational change is now directed to the 3′-carbamate linkage which clearly cannot accommodate the necessary structural differences to the same degree as the phosphodiester. This may be due to the relative conformational rigidity of the carbamate group.

To evaluate the effect of increased numbers of modifications, LNA-CBM_1_ dimers were introduced at three distinct sites within the ON sequences (ON8–13, Table 2, and Fig. 2). In these templates the same destabilising and stabilising effects were seen as with the single addition. In most cases, the change in *T*_m_ per modification was observed to be additive. 5′-LNA modifications were shown to be highly destabilising, reducing *T*_m_ by >30 °C and 23 °C against DNA and RNA respectively. However, the reduction in stability caused by the CBM_1_ backbone in a DNA oligonucleotide hybridised to a DNA target can be reversed by the presence of an adjacent 3′-LNA (ON10) or by a 5′/3′-LNA sandwich in DNA : RNA hybrids (ON11). Combining the results from single and triple incorporations of LNA-CBM_1_ combinations, the trends in duplex stability against DNA targets is as follows: T**x**T^L^ > T**x**T > T^L^**x**T^L^ > T^L^**x**T and against RNA targets T^L^**x**T^L^ > T**x**T^L^ > T**x**T > T^L^**x**T.

#### Hybridisation of the LNA-CBM_2_ modification to DNA and RNA

To determine the backbone with the most favourable biophysical properties, we also prepared oligonucleotides with CBM_2_ linkages and adjacent 3′-LNA sugars (ON7, ON13, Tables 1, 2). Previous studies on triazole^20^ and amide^16^ linkages have shown that the 3′-LNA modification improves duplex stability of modified backbones compared to the unmodified DNA analogue. Addition of LNA to the 3′-side of the CBM_2_-linkage (ON7) gave opposing results to the CBM_1_-linkages (ON4). It caused further destabilisation against DNA targets compared to the DNA-CBM_2_ modification (ON7 *vs*. ON6 = –1.9 °C). Likewise, one incorporation against RNA targets also produced a decrease in stability (ON7 *vs*. ON6 = –2.1 °C). Linkage conformational properties are clearly important,^20^,^31^ the CBM_2_ backbone linkage is significantly destabilising and the LNA sugar is unable to compensate. As was found with CBM_1_ modifications, three incorporations of CBM_2_ produced additive effects compared to single modified templates. Triple addition of the CBM_2_ caused a 22.3 and 27.1 °C reduction in stability against DNA and RNA targets respectively (ON12). Addition of 3′-LNA, a stabilising addition to CBM_1_ modifications, was for CBM_2_ further destabilising against DNA targets (ON13 *vs*. ON12 = –3.2 °C) but stabilising in DNA : RNA hybrids (ON13 *vs*. ON12 = +10.3 °C). This again highlights possible structural and conformational differences between CBM_1_ (x) and CBM_2_ (y) isomers. In summary, for DNA targets we find duplex stability T**y**T > T**y**T^L^ whereas against RNA targets T**y**T^L^ > T**y**T. Summarising *T*_m_ results it is clear that the CBM_1_-linkage has better stabilising properties compared to CBM_2_.

### Circular dichroism

Circular dichroism (CD) was used to evaluate changes in global duplex structure by measurement of polarised light absorption from 200–340 nm (Fig. 2C and D). From these results it was found that the helical conformation remains essentially unperturbed and that structural changes compared to the unmodified control are modest. DNA : DNA duplexes adopt B-form structures characterised by transitions at 220, 255 and 280 nm. Likewise, DNA : RNA hybrids adopt A-like duplexes with negative and positive peaks at 210 and 270 nm. Increasing the proportion of LNA in both duplexes (ON11) results in a hypsochromic shift consistent with reports in the literature.^20^ Comparison of DNA : DNA to DNA : RNA hybrids suggests that LNA has greater structural effects on DNA : DNA B-form duplexes. High levels of LNA within the DNA duplex favour a B → A transition, indicated by hypsochromic shift from 280 nm towards 270 nm and greater deviation from the unmodified control. In DNA : RNA hybrids, the duplex is already in or close to the A-form. Therefore, no significant transition is observed, and the CD spectrum deviates to a lesser extent from the unmodified sequence (additional CD data Fig. S3†).

### Enzymatic stability assays

Enzymatic stability of modified ONs is important to ensure optimal biological half-life and therapeutic efficacy. To evaluate if CBM_1_-LNA modifications enhance enzymatic stability, oligonucleotides were incubated with phosphodiesterase 1 from *C. adamanteus* and foetal bovine serum to determine the stability of the modified templates towards a range of nucleases.

**Fig. 3:**
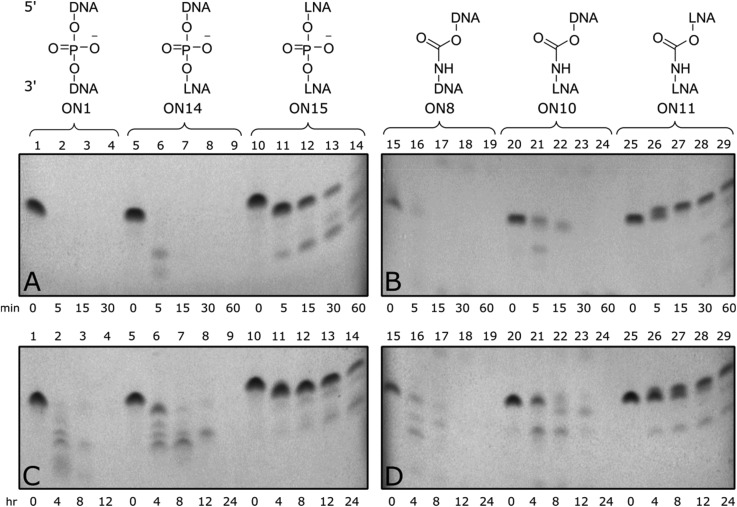
(Top) 20% denatured polyacrylamide gel electrophoresis (PAGE) results from snake venom 3′-exonuclease assay. Samples were incubated in 50 mM Tris-HCl, 10 mM MgCl_2_ buffer pH 9.0 at 37 °C. Aliquots were removed at *t* = 0, 5, 15, 30 and 60 min and frozen before analysis. (A) Gel showing unmodified and LNA controls, (B) gel showing oligonucleotides with carbamate and combined LNA-carbamate modification. ON14 GCTT^L^GCTT^L^CGTT^L^CC, ON15 GCT^L^T^L^GCT^L^T^L^CGT^L^T^L^CC, ^L^ = LNA. Results show increased nuclease resistance when introducing three separate 5′/3′-LNA-CBM_1_ modifications (ON11). (Bottom) 20% denatured polyacrylamide gel electrophoresis (PAGE) results from FBS assay of modified templates containing triple incorporation of CBM_1_-LNA dinucleotides. Samples were incubated in Dulbecco's PBS buffer at 37 °C. Aliquots were removed at *t* = 0, 4, 8, 12 and 24 h and frozen before analysis. (C) Gel showing unmodified and LNA controls, (D) gel showing oligonucleotides with carbamate and combined LNA-carbamate modification. Combined 5′/3′-LNA and CBM_1_ modifications (ON11) showed increased serum resistance compared to unmodified control.

Triply modified ONs were incubated at 37 °C in a phosphate buffer containing snake venom phosphodiesterase 1 and frozen at set time points up to 1 hour (Fig. 3 top). Unmodified control (ON1) was fully degraded within five minutes, along with 3′-LNA control (ON14), indicated by loss of all full-length bands. Triple addition of 5′/3′-LNA showed substantial resistance to enzymatic degradation; only the end nucleotides were excised (Fig. 3A, lane 11). Isolated CBM_1_-linkages were found to offer little resistance towards degradation (Fig. 3B, ON8). However, a combination of 5′/3′-LNA and CBM_1_-linkages (ON11) produced the best resistance, even reducing degradation of the oligonucleotide compared to 5′/3′-LNA (ON11 *vs*. ON15, additional data Fig. S4†). Initial degradation of the 3′-end in LNA/CBM-modified oligonucleotide ON11 leads to a degradation product with a single nucleotide removed. Due to the presence of the neutral carbamate modifications, the overall mass to charge ratio increases, making the oligonucleotide run slower on the gel (Fig. 3B and D, lane 25–29). In contrast, degradation from the 3′-end of the all phosphodiester LNA-modified oligonucleotide ON15 generates a shorter fragment with the expected faster migration (Fig. 3A and C, lane 10–14).

Triply modified ONs were also incubated at 37 °C in foetal bovine serum (FBS) for up to 24 hours and analysed by 20% denatured polyacrylamide gel electrophoresis (PAGE) (Fig. 3 bottom). Much like the snake venom assay, addition of DNA-CBM_1_-linkages or isolated LNA sugars was found to be less effective towards enzymatic resistance (Fig. 3C, ON14, Fig. 3D, ON8) only stable for up to 4–8 hours. When two consecutive LNAs were added (Fig. 3C, ON15), serum enzymes were unable to fully digest the ONs cleaving only terminal regions. Enzymatic resistance can be further enhanced by combining multiple LNA sugars with multiple CBM_1_ linkages (Fig. 3D, ON11).

The above results suggest that nuclease enzymes can digest oligonucleotides that contain single backbone/sugar modifications; hence there is a requirement for multiple modifications to achieve stability. Carbamates themselves are not expected to be especially labile to enzymatic hydrolysis in cells, but many nucleases have large footprints holding several DNA residues close to the catalytic site.^44^ As a result, unmodified phosphodiester linkages within this location could be susceptible to hydrolysis. Multiple LNA/CBM_1_ linkages (as in ON11) presents a region with very little unmodified DNA, such that few phosphodiesters will be within range of the nuclease catalytic site. This greatly inhibits strand cleavage. Overall the enzymatic cleavage experiments show that a combination of multiple 5′/3′-LNA-CBMs provides a high level of stability to enzymatic degradation, while maintaining affinity for RNA targets.^45^

## Conclusions

The combined effects of LNA sugars and DNA backbone analogues on DNA : DNA and DNA : RNA duplex stability are poorly understood. In this paper we have used simple and efficient automated solid-phase methods to synthesise oligonucleotides containing several carbamate-LNA and carbamate-DNA backbone combinations to shed light on this. With or without LNA, the CBM_1_ linkage possesses more favourable duplex stabilising properties than CBM_2_ and was therefore the main focus of this study. LNA either stabilises or destabilises duplexes containing carbamate backbones depending on the location of the LNA sugar relative to the CBM moiety. In DNA : DNA duplexes, and also when the modified DNA strand is paired to an RNA target (DNA : RNA hybrids) addition of LNA to the 3′-side of the CBM_1_ backbone partly compensates for reduced duplex stability resulting from the carbamate linkage, whereas in both DNA duplexes and DNA : RNA hybrids, addition of LNA to the 5′-side of the CBM_1_ backbone is strongly destabilising. The most interesting case is when a 5′/3′-LNA sandwich surrounds CBM_1_. This strongly destabilises DNA : DNA duplexes but the DNA : RNA hybrid is almost as stable as the unmodified duplex; for a 14-mer containing three such modified backbone linkages the DNA : RNA hybrid is a remarkable 20 °C more stable than the DNA duplex. This is significant because in general the biological target for antisense oligonucleotides is RNA, and differentiation between DNA and RNA targets is important. Another key factor to consider in a cellular context is stability to degradation. Nuclease digestion studies indicate that LNA-CBM_1_ modifications provide improved enzymatic resistance compared to LNA or CBM alone. Finally, LNA-CBM_1_ linkages cannot be degraded by phosphodiesterase enzymes into 5′-LNA mononucleotides; hence oligonucleotides containing this dual modification should not give rise to LNA fragments that could be incorporated into genomic DNA *in vivo*. Consequently they might have altered (potentially more favourable) toxicological profiles compared oligonucleotides which contain phosphodiester backbones or close analogues. Taking the above properties into account, LNA-carbamate oligonucleotides, and potentially other combinations of LNA with artificial DNA backbones, could be future candidates for use in diagnostic, therapeutic and *in vivo* imaging applications.

## Conflicts of interest

There are no conflicts to declare.

## Supplementary Material

Supplementary information
