# Supplementary material for: Synthesis and biophysical properties of carbamate-locked nucleic acid (LNA) oligonucleotides with potential antisense applications
Source: Org Biomol Chem. 2019 May 17;17(21):5341–8. doi: 10.1039/c9ob00691e (PMC6686644; doi:10.1039/c9ob00691e)
Supplement: Supplementary file 1 [file OB-017-C9OB00691E-s001.pdf]

Electronic Supplementary Material (ESI) for Organic & Biomolecular Chemistry.
This journal is © The Royal Society of Chemistry 2019

Synthesis and Biophysical Properties of Carbamate-Locked Nucleic Acid (LNA) Oligonucleotides for Antisense Applications

Cameron Thorpe[a], Sven Epple[a], Benjamin Woods[a], Afaf El-Sagheer[ab], Tom Brown[a]

[a] *Department of Chemistry, University of Oxford, 12 Mansfield Road, Oxford, OX1 3TA, UK.*
[b] *Department of Science and Mathematics, Faculty of Petroleum and Mining Engineering, Suez University, Suez 43721, Egypt.*

tom.brown@chem.ox.ac.uk

Contents

General Synthetic Procedures 2
Synthesis of CBM_1 dinucleotide phosphoramidites 3
Synthesis of CBM_2 dinucleotide phosphoramidites 11
Synthesis and purification of oligonucleotides 14
Synthesis of DNA oligonucleotides 14
Synthesis of RNA oligonucleotides 15
Removal of 2′-*O*-TBDMS protecting groups on RNA oligonucleotides 15
Purification of oligonucleotides 15
Biophysical studies and assays 16
Ultraviolet melting studies 16
Additional UV-data 16
Circular dichroism studies 17
Snake venom assay 18
Foetal bovine serum (FBS) assay 18
Gel quantification 19
HPLC spectra 19
NMR spectra 27
References 46

General Synthetic Procedures

All reagents were purchased from Sigma-Aldrich, Acros Organics, Fluka and Fisher Scientific, FluoroChem, Jena Bioscience or Alfa Aesar and used without further purification. Dry solvents (pyridine, Et_3N, CH_2Cl_2, THF, MeCN) were obtained using an MBraun SPS Bench Top solvent purification system (SPS). All air/moisture sensitive reactions were carried out under inert atmosphere (argon) in oven-dried glassware. Solvents used for phosphitylation reactions and purification were also degassed by saturating the solvents with argon for 20 min. Reactions were monitored by thin layer chromatography (TLC) using Merck Kieselgel 60 F24 silica gel plates (0.22 mm thickness, aluminium backed). The compounds were visualized by UV irradiation at 254/265 nm and by staining in *p*-anisaldehyde or ninhydrin solution followed by gentle heating. Silica column chromatography was performed using Merck Geduran® 60 Å (40–62 µm) or using a Biotage Isolera™ Prime auto column (10-100 g SNAP ultra-cartridges). ^{1}H NMR (400 MHz), ^{13}C NMR (101 MHz) and ^{31}P NMR (162 MHz) were recorded using a Bruker DPX 400 (AVIIIHD 400) or Bruker AVII 500 spectrometer (^{1}H-500 MHz, ^{13}C-126 MHz) and referenced to an appropriate deuterated solvent. Assignment was aided by DEPT-135, COSY, HSQC-DEPT and HMBC spectra. Data was then processed using MestreNova software. Low resolution mass spectrometry (LRMS) using electrospray ionisation positive (ESI+) and negative (ESI-) modes was recorded on a Waters ZMD quadrupole mass spectrometer in HPLC grade MeOH/MeCN. High resolution mass spectrometry (HRMS) using electrospray ionisation was recorded on a Bruker APEX III FT-ICR mass spectrometer. Oligonucleotide mass spectrometry was recorded on a UPLC-MS Waters XEVO G2-QTOF (ESI-) using an ACQUITY UPLC oligonucleotide BEH C18 column, 130 Å (1.7 µm, 2.1 mm × 50 mm). Data was then deconvoluted using MassLynx v4.1. A gradient of MeOH in Et_3N and hexafluoroisopropanol (HFIP) was used (buffer A, 8.6 mM Et_3N, 200 mM HFIP in 5% MeOH/H_2O (v/v); buffer B, 20% buffer A in MeOH). Buffer B was increased from 0–70% over 8 min, at a flow rate of 0.2 mL min^{-1}.

Synthesis of CBM_1 dinucleotide phosphoramidites

3′-*O*-Carbonylimidazole-5′-*O*-dimethoxytrityl-LNA-thymidine **(4)**

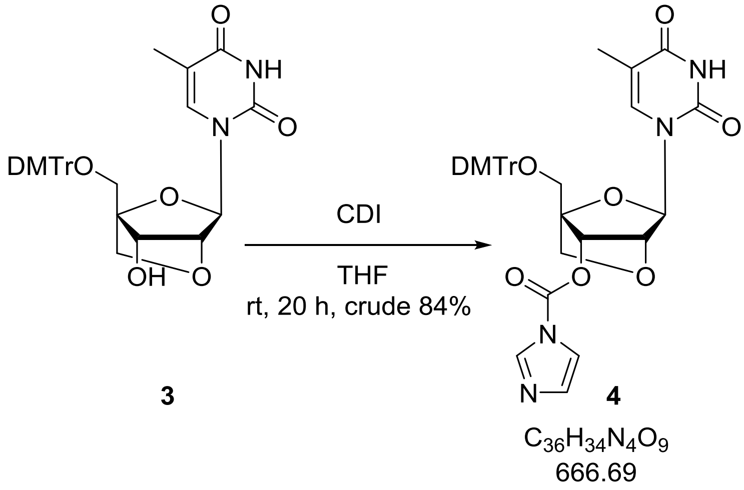

Compound **3** (1.11 g, 1.94 mmol) and 1,1′-carbonyldiimidazole (0.630 g, 3.88 mmol) were separately co-evaporated in dry THF (3 × 5.00 mL), and each re-dissolved in dry THF (20.0 mL). The solution of compound **3** was then added dropwise to a solution of CDI over 1 h and stirred for further 20 h at rt. The solvent was removed under reduced pressure and re-dissolved in EtOAc (100 mL), washed with 5% KH_2PO_4 (2 × 100 mL) and dried over Na_2SO_4. Removal of the solvent under reduced pressure gave intermediate **4** (1.08 g, 1.61 mmol, crude 84%) as a white foam which was used without further purification. R_f 0.33 (CH_2Cl_2:MeOH, 19:1); HRMS–ESI (*m/z*): $[M + H]^+$ calcd for $C_{36}H_{35}N_4O_9$, 667.2399; found, 667.2393.

5′-Azido-3′-benzyl-5′-deoxy-LNA-thymidine **(7)**[1]

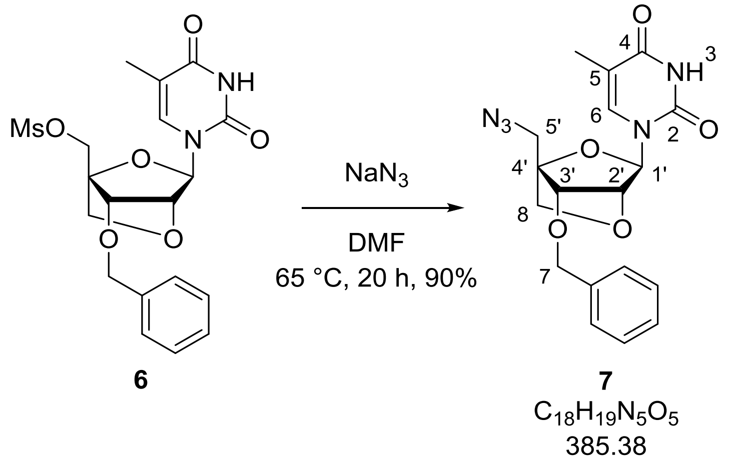

To a solution of compound **6** (1.00 g, 2.28 mmol) in DMF (7.00 mL) was added sodium azide (1.01 g, 15.5 mmol) and stirred at 65 °C for 20 h. The solvent was removed under reduced pressure, re-suspended in EtOAc (70.0 mL) and filtered over silica. Evaporation of the solvent gave an oil which was purified by silica column chromatography (Petroleum ether:EtOAc, 3:7 to 0:10) to give azide **7** (0.790 g, 2.05 mmol, 90%) as a white solid. R_f 0.60 (CH_2Cl_2:MeOH, 9:1); IR (cm^{-1}): 2102; ^1H NMR (400

MHz, $CDCl_3$) δ 8.88 (bs, 1H, H-3), 7.40 (d, *J* = 1.2 Hz, 1H, H-6), 7.39 – 7.27 (m, 5H, H-Ar), 5.64 (s, 1H H-1′), 4.66 (d, *J* = 11.4 Hz, 1H, H-8), 4.58 (s, 1H, H-2′), 4.54 (d, *J* = 11.4 Hz, 1H, H-8), 4.03 (d, *J* = 7.9 Hz, 1H, H-7), 3.87 (s, 1H, H-3′), 3.84 (d, *J* = 7.9 Hz, 1H, H-7), 3.75 (d, *J* = 13.7 Hz, 1H, H-5′), 3.67 (d, *J* = 13.7 Hz, 1H, H-5′), 1.94 (d, *J* = 1.2 Hz, 3H, CH_3); ^{13}C NMR (101 MHz, $CDCl_3$) δ 163.7 (C-4), 149.8 (C-2), 136.7 (C-Ar), 134.4 (C-6), 128.7 (C-Ar), 128.5 (C-Ar), 128.0 (C-Ar), 110.8 (C-5), 87.8 (C-1′), 86.9 (C-4′), 76.8 (C-2′), 76.4 (C-3′), 72.5 (C-7), 72.5 (C-8), 47.5 (C-5′), 12.9 (CH_3); HRMS–ESI (*m/z*): $[M + H]^+$ calcd for $C_{18}H_{20}N_5O_5$, 386.1459; found, 386.1460.

5′-Amino-5′-deoxy-LNA-thymidine **(8)**[1]

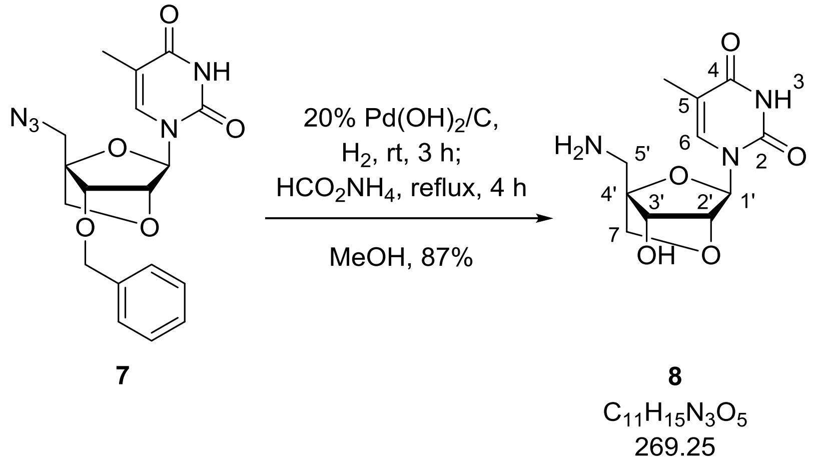

To a solution of azide **7** (1.50 g, 3.90 mmol) in MeOH (70.0 mL) was added 20% $Pd(OH)_2$/C (338 mg, 2.41 mmol) and stirred under H_2 atmosphere at rt for 3 h. After complete consumption of the starting material by TLC (CH_2Cl_2:MeOH, 17:1), ammonium formate (1.84 g, 29.2 mmol) was added and the reaction was refluxed under argon for 4 h. The reaction mixture was diluted with CH_2Cl_2 (30.0 mL) and MeOH (30.0 mL), filtered over Celite and concentrated under reduced pressure. The residue was purified by silica column chromatography (CH_2Cl_2:MeOH, 97:3 to 19:1 + 1% Et_3N) to give amine **8** (0.960 g, 3.58 mmol, 87%) as a white solid. R_f 0.25 (CH_2Cl_2:MeOH:Et_3N, 84:15:1); ^1H NMR (400 MHz, D_2O) δ 7.53 (d, *J* = 1.4 Hz, 1H, H-6), 5.66 (s, 1H, H-1′), 4.50 (s, 1H, H-2′), 4.20 (s, 1H, H-3′), 4.02 (q, *J* = 8.6 Hz, 2H, H-7), 3.37 (d, *J* = 14.5 Hz, 1H, H-5′), 3.29 (d, *J* = 14.3 Hz, 1H, H-5′), 1.88 (d, *J* = 1.2 Hz, 3H, CH_3); ^{13}C NMR (101 MHz, D_2O) δ 166.7 (C-4), 151.2 (C-2), 136.0 (C-6), 110.6 (C-5), 86.9 (C-4′), 86.9 (C-1′), 79.4 (C-2′), 71.6 (C-7), 70.4 (C-3′), 37.0 (C-5′), 11.6 (CH_3); HRMS–ESI (*m/z*): $[M + H]^+$ calcd for $C_{11}H_{16}N_3O_5$, 270.1085; found, 270.1085.

5′-*O*-(Dimethoxytrityl)-DNA-CBM$_1$-LNA-thymidine dimer (DNA-CBM$_1$-LNA) **(10)**

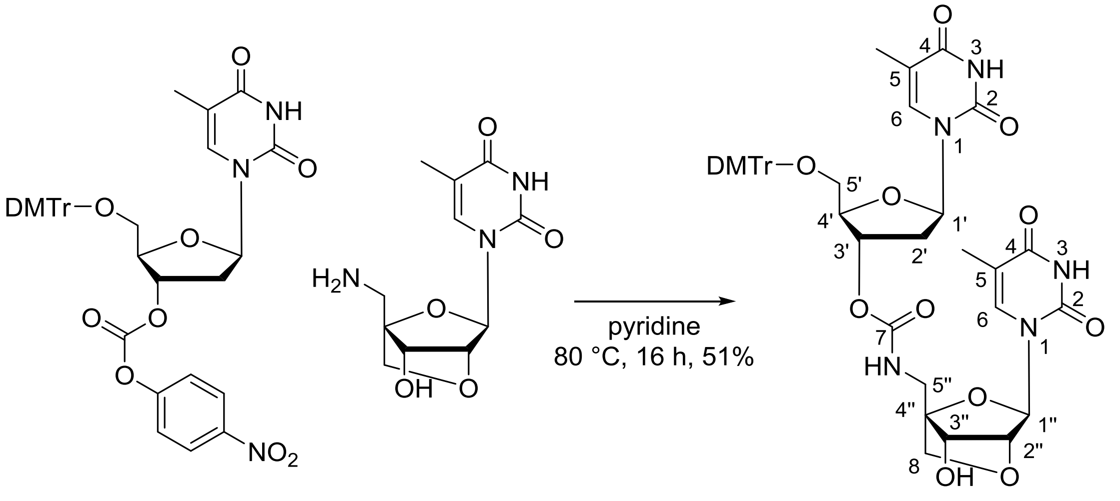

2 **8** **10**

$C_{43}H_{45}N_5O_{13}$
839.85

Amine **8** (282 mg, 1.05 mmol) and carbonate **2** (750 mg, 1.06 mmol) were separately co-evaporated with dry pyridine (3 × 5.00 mL) and each dissolved in dry pyridine (5.00 mL). The solution of carbonate **2** was added dropwise to amine **8** and stirred at 80 °C for 16 h. The reaction was concentrated under reduced pressure and purified by silica column chromatography (EtOAc:MeOH, 49:1 to 25:1 + 1% Et_3N). This gave a white solid which was re-dissolved in $CHCl_3$: ^{i}PrOH, [3/2, (v/v), 50 mL] and washed with sat. aq $NaHCO_3$ until the aq layer stayed colourless. The organic layer was dried over Na_2SO_4 and filtered over Celite. Evaporation of the solvents gave dimer **10** (452 mg, 0.540 mmol, 51%) as a white solid. R_f 0.38 (CH_2Cl_2:MeOH, 9:1); ^{1}H NMR (400 MHz, DMSO-d_6) δ 11.39 (s, 1H, H-3), 11.37 (s, 1H, H-3), 7.79 (t, *J* = 6.2 Hz, 1H, NH CBM), 7.52 (d, *J* = 1.0 Hz, 1H, H-6), 7.42 (d, *J* = 1.2 Hz, 1H, H-6), 7.40 – 7.19 (m, 9H, H-Ar), 6.89 (dq, *J* = 9.5, 2.5 Hz, 4H, H-Ar), 6.25 (dd, *J* = 8.6, 5.9 Hz, 1H, H-1′), 5.57 (d, *J* = 4.1 Hz, 1H, OH), 5.41 (s, 1H, H-1″), 5.27 (dd, *J* = 5.5, 3.3 Hz, 1H, H-3′), 4.12 (s, 1H, H-2″), 4.05 (q, *J* = 3.3 Hz, 1H, H-4′), 3.89 – 3.82 (m, 2H, H-8, H-3″), 3.73 (s, 6H, OCH_3), 3.62 (d, *J* = 8.0 Hz, 1H, H-8), 3.54 (t, *J* = 6.2 Hz, 2H, H-5″), 3.31 (d, *J* = 3.9 Hz, 1H, H-5′), 3.21 (dd, *J* = 10.4, 3.1 Hz, 1H, H-5′), 2.45 (q, *J* = 10.0 Hz, 1H, H-2′), 2.29 (dq, *J* = 10.0 , 4.7, 3.3 Hz, 1H, H-2′), 1.76 (d, *J* = 1.2 Hz, 3H, CH_3), 1.40 (d, *J* = 1.0 Hz, 3H, CH_3); ^{13}C NMR (101 MHz, DMSO-d_6) δ 164.3 (C-4), 164.1 (C-4), 158.7 (C-Ar), 158.7 (C-Ar), 156.3 (C-7), 150.9 (C-2), 150.4 (C-2), 145.1 (C-Ar), 135.9 (C-Ar), 135.8 (C-6), 135.6 (C-Ar), 135.1 (C-6), 130.2 (C-Ar), 128.4 (C-Ar), 128.1 (C-Ar), 127.3 (C-Ar), 113.7 (C-Ar), 110.4 (C-5), 109.0 (C-5), 87.6 (C-4″), 86.8 (C-1″), 86.5, 84.1 (C-1′), 83.8 (C-4′), 79.5 (C-2″), 75.1 (C-3′), 71.9 (C-8), 70.3 (C-3″), 64.2 (C-5″), 55.5 (OCH_3), 37.7 (C-5′), 37.2 (C-2′), 12.8 (CH_3), 12.1 (CH_3); HRMS–ESI (*m/z*): $[M + Na]^+$ calcd for $C_{43}H_{45}N_5O_{13}Na$, 862.2906; found, 862.2906.

5′-*O*-(Dimethoxytrityl)-LNA-CBM$_1$-DNA-thymidine dimer (LNA-CBM$_1$-DNA) **(11)**

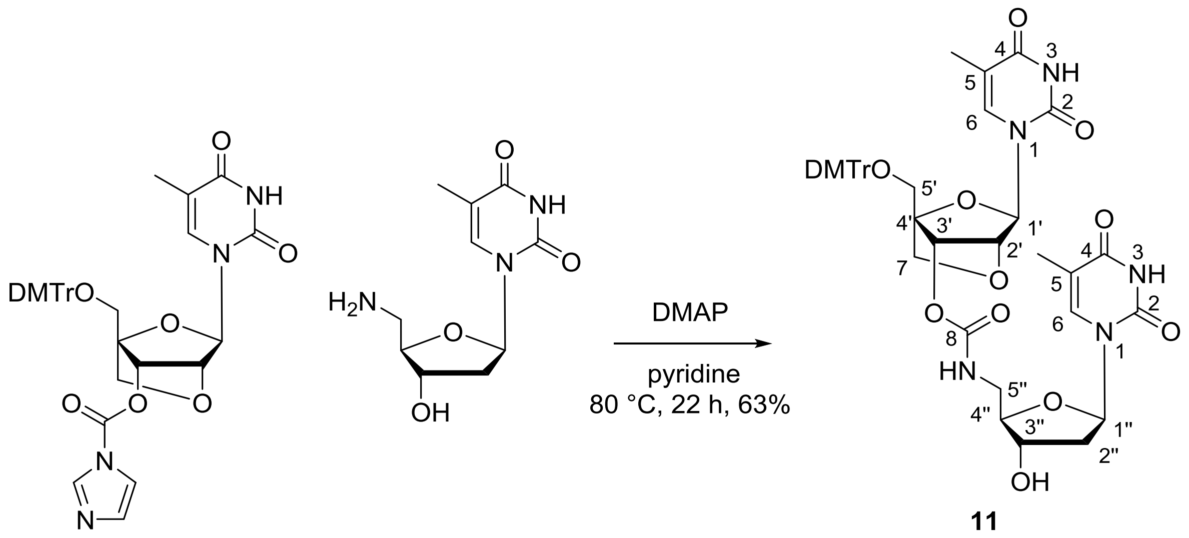

4 **5** **11**

$C_{43}H_{45}N_5O_{13}$
839.86

Intermediate **4** (542 mg, 0.813 mmol) and amine **5** (196 mg, 0.813 mmol) were separately co-evaporated in dry pyridine (2 × 10.0 mL) and each re-dissolved in dry pyridine (5.00 mL). To the solution of amine **5** was added intermediate **4** and stirred under argon at 80 °C for 20 h. A catalytic amount of DMAP (9.93 mg, 0.0813 mmol) was added and the reaction was stirred for a further 2 h at 80 °C. The solvent was removed under reduced pressure, re-dissolved in CH_2Cl_2 (70.0 mL) and extracted with 5% KH_2PO_4. The aq layer was back extracted with CH_2Cl_2 (2 × 70.0 mL) and dried over Na_2SO_4 and purified by silica column chromatography (EtOAc:MeOH, 99:0 to 90:9 + 1% Et_3N) to give dimer **11** (0.430 g, 0.510 mmol, 63%) as a white solid. R_f 0.16 (CH_2Cl_2:MeOH, 93:7); ^{1}H NMR (400 MHz, DMSO-d_6) δ 11.57 (s, 1H, H-3), 11.37 (s, 1H, H-3), 7.82 (t, *J* = 6.0 Hz, 1H, NH CBM), 7.62 (d, 1H, *J* = 1.2 Hz, H-6), 7.56 (d, 1H, *J* = 1.2 Hz, H-6), 7.50 – 7.44 (m, 2H, H-Ar), 7.43 – 7.30 (m, 7H, H-Ar), 6.97 (dd, *J* = 9.0, 2.1 Hz, 4H, H-Ar), 6.20 (dd, *J* = 7.7, 6.4 Hz, 1H, H-1″), 5.59 (s, 1H, H-1′), 5.38 (d, J = 4.0 Hz, 1H, OH), 5.18 (s, 1H, H-3′), 4.50 (s, 1H, H-2′), 4.21– 4.16 (m, 1H, H-3″), 3.88 (d, *J* = 8.2 Hz, 1H, H-7), 3.80 (s, 7H, H-4″, OCH_3), 3.45 (2 × d *J* = 11.5 Hz, 2H, H-5′), 3.34 – 3.22 (m, 2H, H-5″), 2.14 – 2.08 (m, 2H, H-2″), 1.82 (d, *J* = 1.2 Hz, 3H, CH_3), 1.65 (d, *J* = 1.2 Hz, 3H, CH_3); ^{13}C NMR (101 MHz, DMSO-d_6) δ 164.2 (C-4), 158.6 (C-Ar), 155.3 (C-8), 150.9 (C-2), 150.4 (C-2), 145.0 (C-Ar) , 136.5 (C-6), 135.6 (C-Ar), 135.3 (C-Ar), 134.2 (C-6), 130.2 (C-Ar), 130.1 (C-Ar), 128.5 (C-Ar), 128.0 (C-Ar), 127.3 (C-Ar), 113.8 (C-Ar), 110.1 (C-5), 109.4 (C-5), 86.9 (C-1″), 86.7 (C-4′), 86.4 (C_{quat}DMTr), 85.4 (C-4″), 84.6 (C-1′), 78.0 (C-2′), 72.2 (C-7), 71.6 (C-3″), 71.3 (C-3′), 55.5 (OCH_3), 43.6 (C-5″), 38.9 (C-2″), 12.9 (CH_3), 12.3 (CH_3); HRMS–ESI (*m/z*): $[M+Na]^+$ calcd for $C_{43}H_{45}N_5O_{13}Na$, 862.2906, found 862.2906.

5′-*O*-(Dimethoxytrityl)-LNA-CBM$_1$-LNA-thymidine dimer (LNA-CBM$_1$-LNA) **(12)**

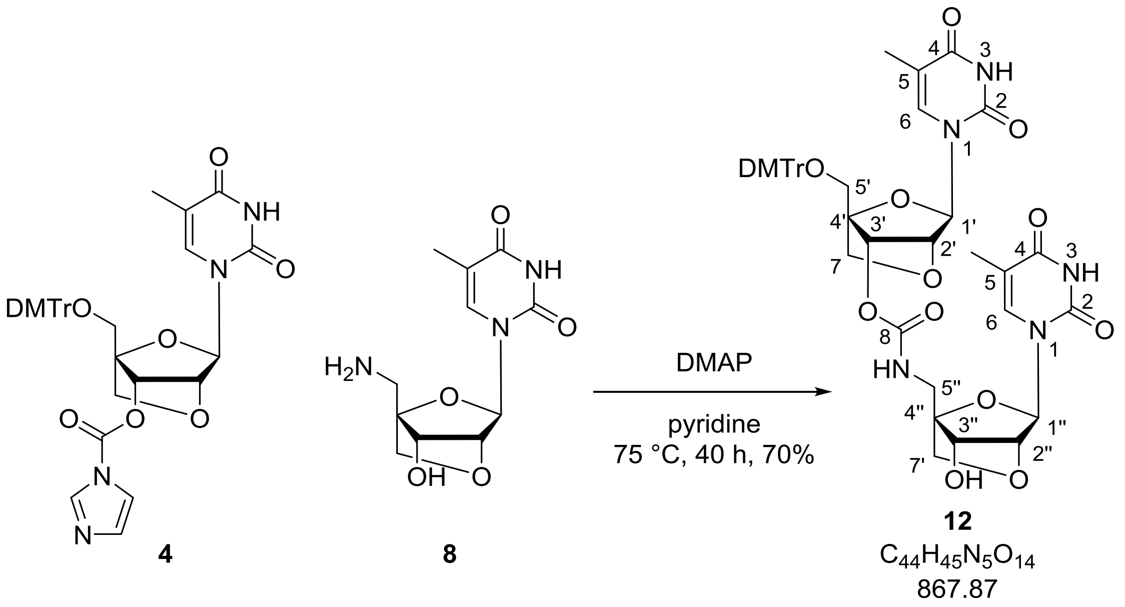

Amine **8** (202 mg, 0.750 mmol) and intermediate **4** (500 mg, 0.750 mmol) were separately co-evaporated with dry pyridine (3 × 5.00 mL) and each dissolved in dry pyridine (5.00 mL). Intermediate **4** was added dropwise to the solution of amine **8** and the reaction was stirred at 80 °C for 16 h. A catalytic amount of DMAP (9.00 mg, 0.08 mmol) was added and the mixture was stirred at 80 °C for another 24 h. The reaction was then cooled to rt and the solvents were evaporated under reduced pressure. The remaining solid was re-dissolved in CH_2Cl_2 (70.0 mL) and the organic phase was washed with a sat. aq $NaHCO_3$ (2 × 70.0 mL), brine (50.0 mL) and dried over $MgSO_4$. The crude was concentrated under reduced pressure and purified by silica column chromatography (EtOAc:MeOH, 100:0 to 96:4 + 1% Et_3N) to give dimer **12** (453 mg, 520 µmol, 70%) as a white solid. R_f 0.41 (EtOAc:MeOH:Et_3N, 25:4:1); ^{1}H NMR (400 MHz, DMSO-d_6) δ 11.50 (s, 1H, H-3), 11.39 (s, 1H, H-3), 7.91 (t, *J* = 6.1 Hz, 1H, NH CBM), 7.63 – 7.48 (m, 1H, H-6), 7.45 – 7.17 (m, 10H, H-Ar, H-6)), 6.96 – 6.81 (m, 4H, H-Ar), 5.62 (d, *J* = 4.1 Hz, 1H, OH), 5.53 (s, 1H, H-1′ or H-1′′), 5.39 (s, 1H, H-1′ or H-1′′), 5.18 (s, 1H, H-3′), 4.47 (s, 1H, H-2′), 4.11 (s, 1H, H-2′′), 3.91 – 3.76 (m, 5H, H-7, H-7′, H-3′′), 3.72 (d, *J* = 4.1 Hz, 6H, OCH_3), 3.65 – 3.47 (m, 2H, H-5′ or H-5′′), 3.47 – 3.38 (m, 2H, H-5′ or H-5′′), 1.72 (d, J = 1.1 Hz, 3H, CH_3), 1.55 (d, J = 1.1, 3H, CH_3); ^{13}C NMR (101 MHz, DMSO-d_6) δ 163.8 (C-4), 163.7 (C-4), 158.2 (C-Ar), 158.2 (C-Ar), 155.0 (C-8), 150.0 (C-2), 149.9 (C-2), 144.5 (C-Ar), 135.0 (C-Ar), 134.9 (C-Ar), 134.8 (C-6), 133.8 (C-6), 129.7 (C-Ar), 128.0 (C-Ar), 127.6 (C-Ar), 126.9 (C-Ar), 113.3 (C-Ar), 109.0 (C-5), 108.5 (C-5), 87.1 (C_{quat}DMTr), 86.4 (C-1′ or C-1′′), 86.3 (C-1′ or C-1′′), 85.9 (C-4′, C-4′′), 79.0 (C-2′′), 77.5 (C-2′), 71.7(C-7, C-7′) 71.4 (C-5′), 70.9 (C-3′), 70.2 (C-3′′), 55.1 (OCH_3), 55.0 (OCH_3), 12.4 (CH_3), 12.2 (CH_3); HRMS–ESI (m/z): $[M + Na]^+$ calcd for $C_{44}H_{45}N_5O_{14}Na$, 890.2855; found, 890.2857.

5′-*O*-(Dimethoxytrityl)-DNA-CBM$_1$-LNA-thymidine phosphoramidite dimer (DNA-CBM$_1$-LNA) **(14)**

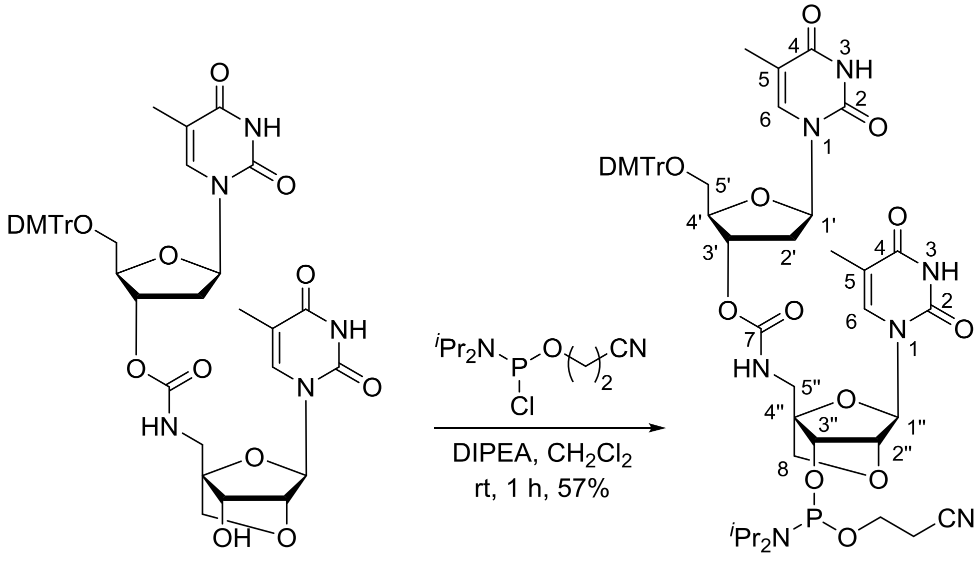

10

14
$C_{52}H_{62}N_7O_{14}P$
1040.08

To a solution of dimer **10** (480 mg, 0.570 mmol) in dry, degassed CH_2Cl_2 (5.00 mL), was added degassed DIPEA (250 µL, 1.44 mmol) followed by chloro(diisopropylamino)-*β*-cyanoethoxyphosphine (180 µL, 0.810 mmol) dropwise over 10 min. After 1 h at rt, a second portion of chloro(diisopropylamino)-*β*-cyanoethoxyphosphine (120 µL, 0.540 mmol) and DIPEA (120 µL, 0.690 mmol) was added and stirred for another 1 h. The reaction mixture was diluted with dry CH_2Cl_2 (10.0 mL) and washed with sat. aq KCl (10.0 mL). The organic layer was dried over Na_2SO_4 and the solvents were evaporated under reduced pressure. The solid was re-dissolved in dry, degassed CH_2Cl_2 (2.50 mL) and precipitated by addition of degassed hexane (250 mL). The suspension was cooled to 4 °C and the supernatant was decanted. The remaining slurry was dried under reduced pressure to give a white solid. Purification by column chromatography (Hexane: EtOAc, 1:10 + 1% pyridine) gave building block **14** (338 mg, 0.320 mmol, 57%) as a white solid. R_f 0.51 (hexane:EtOAc:pyridine, 10:100:1); ^{31}P NMR (162 MHz, $CDCl_3$) δ 149.5, 149.1; HRMS–ESI (*m/z*): $[M + H]^+$ calcd for $C_{52}H_{63}N_7O_{14}P$, 1040.4165, found, 1040.4183.

5′-*O*-(Dimethoxytrityl)-LNA-CBM$_1$-DNA-thymidine phosphoramidite dimer (LNA-CBM$_1$-DNA) **(15)**

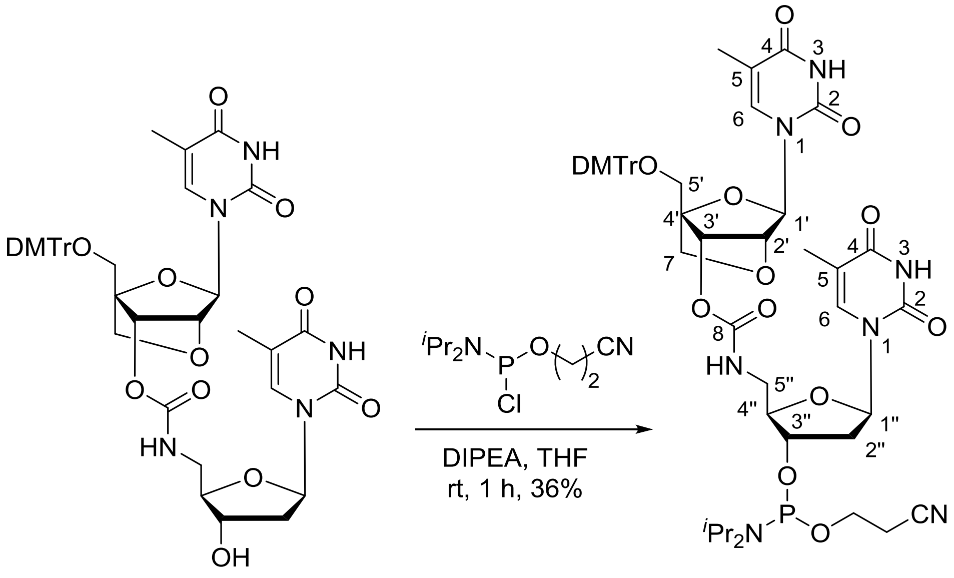

11

15
$C_{52}H_{62}N_7O_{14}P$
1040.08

Carbamate dimer **11** (303 mg, 0.360 mmol) was dissolved in degassed, anhydrous THF (5.00 mL). Degassed DIPEA (199 µL, 1.14 mmol) and chloro(diisopropylamino)-*β*-cyanoethoxyphosphine (128 µL, 0.571 mmol) were added and the solution was stirred at rt under argon for 1 h. The solvents were removed under reduced pressure and the residue was re-dissolved in CH_2Cl_2 (5.00 mL). This was washed with sat. aq KCl (10.0 mL), dried over Na_2SO_4 and concentrated under reduced pressure, to form a glassy white solid. The solid was then re-dissolved in dry CH_2Cl_2 (1.00 mL) and stirred vigorously. Upon addition of dry degassed hexane (30.0 mL), the product precipitated as a white solid. The precipitation was repeated five times, and the solid was purified with silica column chromatography (EtOAc:Hexane, 85:14 +1% Et_3N) using degassed dry solvents to give phosphoramidite **15** (136 mg, 0.130 mmol, 36%) as a white solid. R_f 0.73 (EtOAc:MeOH:Et_3N, 95:4:1); ^{31}P NMR (162 MHz, DMSO-d_6) 146.9, 146.9; HRMS–ESI (*m/z*): $[M + H]^+$ calcd for $C_{52}H_{63}N_7O_{14}P$, 1040.4174, found 1040.4165.

5′-*O*-(Dimethoxytrityl)-LNA-CBM$_1$-LNA-thymidine phosphoramidite dimer (LNA-CBM$_1$-LNA) **(16)**

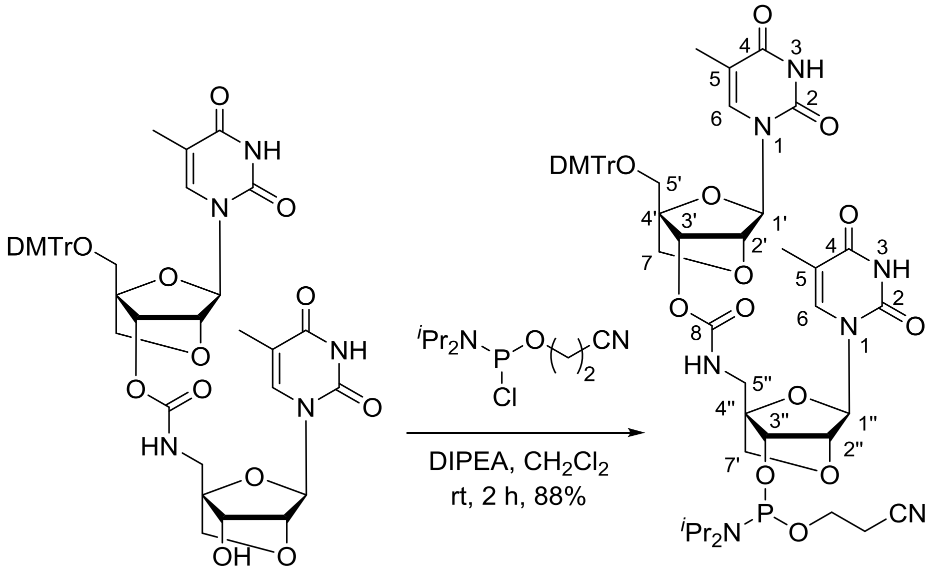

12

16
$C_{53}H_{62}N_7O_{15}P$
1068.09

To a solution of dimer **12** (334 mg, 0.380 mmol) in dry degassed CH_2Cl_2 (3.50 mL) was added degassed DIPEA (165 µL, 950 µmol). Chloro(diisopropylamino)-*β*-cyanoethoxyphosphine (120 µL, 540 µmol) was added dropwise over 10 min and the reaction was stirred at rt for 1 h. After 1 h, a second portion of chloro(diisopropylamino)-*β*-cyanoethoxyphosphine (100 µL, 450 µmol) was added and stirred for another 1 h. The reaction mixture was diluted with dry degassed CH_2Cl_2 (10.0 mL) and washed with sat. aq KCl (10.0 mL) under an argon atmosphere. The organic layer was dried over Na_2SO_4 and the solvents were evaporated under reduced pressure. The solid was re-dissolved in dry degassed CH_2Cl_2 (2.50 mL) and precipitated by addition of hexane (150 mL). The supernatant was decanted and the solid dried under reduced pressure. The precipitation was repeated followed by silica column chromatography (Hexane:EtOAc, 3:20 + 1% pyridine) under argon atmosphere giving phosphoramidite **16** (356 mg, 0.330 mmol, 88%) as a white solid. R_f 0.57 (Hexane:EtOAc:pyridine, 9:90:1); ^{31}P NMR (162 MHz, DMSO-d_6) δ 148.1, 147.5; HRMS–ESI (*m/z*): $[M + H]^+$ calcd for $C_{53}H_{63}N_7O_{15}P$, 1068.4114, found, 1068.4109.

Synthesis of CBM_2 dinucleotide phosphoramidites

3′-*O*-(Benzyl)-5′-*O*-(4-nitrophenyl formate)-LNA-thymidine **(21)**

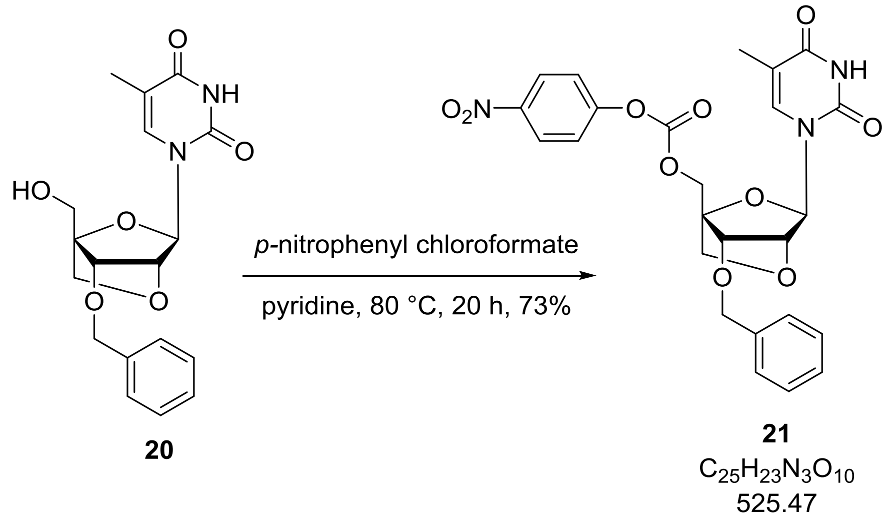

20

21
$C_{25}H_{23}N_3O_{10}$
525.47

To a solution of alcohol[2] **20** (1.24 g, 3.40 mmol) in anhydrous pyridine (20.0 mL) was added *p*-nitrophenyl chloroformate (830 mg, 4.10 mmol) at rt and then heated to 80 °C. After 20 h, a second portion of *p*-nitrophenyl chloroformate (89.0 mg, 0.340 mmol) was added and stirred at 80 °C for another 3 h. The reaction was concentrated under reduced pressure and purified by silica column chromatography (CH_2Cl_2:EtOAc, 50:50) to give carbonate **21** (1.30 g, 2.40 mmol, crude 73%) as a white foam. R_f 0.68 (CH_2Cl_2:MeOH, 19:1); HRMS–ESI (*m/z*): $[M + Na]^+$ calcd for $C_{25}H_{23}N_3O_{10}Na$, 548.1276, found 548.1266.

5′-*O*-(Dimethoxytrityl)-3′-*O*-(*tert*-butyldimethylsilyl)-DNA-CBM_2-DNA-thymidine dimer (DNA-CBM_2-DNA) **(22)**

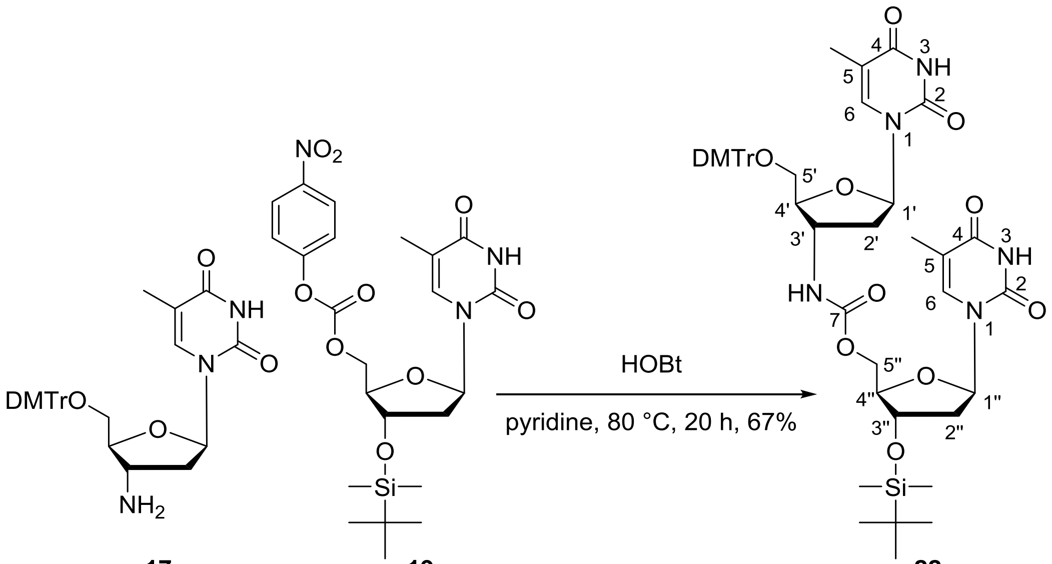

17 **19** **22**
$C_{48}H_{59}N_5O_{12}Si$
926.11

To a solution of carbonate **19** (570 mg, 1.09 mmol) and HOBt (140 mg, 1.18 mmol) in dry pyridine (35.0 mL) was added amine **17** (580 mg, 1.07 mmol) and stirred at 80 °C for 20 h. The reaction was

then concentrated under reduced pressure and the residue was diluted with EtOAc (100 mL). The solution was washed with H_2O (2 × 100 mL), brine (100mL) and dried over Na_2SO_4. Purification by silica column chromatography (EtOAc:Petroleum ether, 1:9 to 10:0) gave dimer **22** (660 mg, 0.710 mmol, 67%) as a white foam. R_f 0.46 (EtOAc); ^{1}H NMR (400 MHz, $CDCl_3$) δ 9.30 (2 × s, 2H, H-3), 7.54 (d, *J* = 1.4 Hz, 1H, H-6), 7.37 – 7.30 (m, 2H, H-Ar), 7.26 – 7.07 (m, 10H, H-Ar, H-6), 6.80 – 6.72 (m, 4H, H-Ar), 6.41 (d, *J* = 7.5 Hz, 1H, NHCBM), 6.33 (t, *J* = 6.8 Hz, 1H, H-1′ or H-1″), 6.00 (t, *J* = 6.5 Hz, 1H, H-1′ or H-1″), 4.45 (br s, 1H, H-3″), 4.31 (d, *J* = 5.8 Hz, 1H, H-3′), 4.19 (m, 2H, H-5″), 3.94 (m, 2H, H-4′, H-4″), 3.71 (d, *J* = 5.9 Hz, 6H, OCH_3), 3.38 (dd, *J* = 10.7, 2.7 Hz, 1H, H-5′), 3.32 (dd, *J* = 10.5, 2.7 Hz, 1H, H-5′), 2.33 – 2.16 (m, 4H, H-2′, H-2″), 1.78 (app s, 3H, CH_3), 1.33 (app s, 3H, CH_3), 0.81 (s, 9H, ^{t}Bu-Si), 0.00 (2 × s, 6H, Si-CH_3); ^{13}C NMR (101 MHz, $CDCl_3$) δ 164.1 (C-4), 158.7-158.6 (C-Ar, C-7), 150.8 (C-2), 147.4 (C-Ar), 144.3 (C-Ar), 136.7 (C-Ar), 135.9 (C-6), 130.1 (C-Ar), 129.1 (C-Ar), 128.2-127.0 (7 × C-Ar, C-6), 113.1 (C-Ar), 110.9 (C-5), 87.0 (C-1′ or C-1″), 84.5 (C_{quat}DMTr, C-4′, C-4″, [C-1′ or C-1″]), 71.6 (C-3′), 64.2 (C-5″), 63.7 (C-5′), 55.3 (OCH_3), 52.4 (C-3″) 40.2 (C-2′ or C-2″), 38.4 (C-2′ or C-2″), 25.7 (C-Si-C($\underline{C}H_3$), 17.9 (C-Si-$\underline{C}(CH_3)$), 12.5 (CH_3), 11.7 (CH_3), –4.7 (C-Si$\underline{C}H_3$), –4.9 (C-Si$\underline{C}H_3$); HRMS–ESI (*m/z*): $[M + Na]^+$ calcd for $C_{48}H_{59}N_5O_{12}SiNa$, 948.3822 found 948.3830.

3″-*O*-(Benzyl)-5′-*O*-(dimethoxytrityl)-DNA-CBM$_2$-LNA-thymidine dimer (DNA-CBM$_2$-LNA) **(25)**

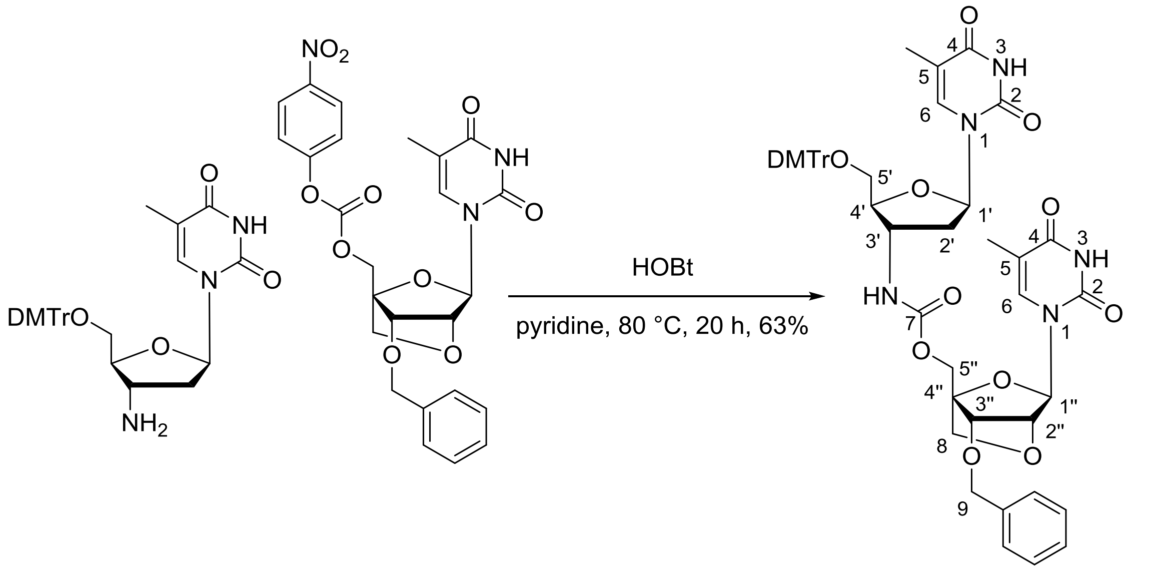

To a solution of carbonate **21** (0.960 g, 1.90 mmol) and HOBt (25.0 mg, 0.190 mmol) in dry pyridine (60.0 mL) was added amine **17** (1.03 g, 1.90 mmol) and stirred at 80 °C for 20 h. The reaction was concentrated under reduced pressure and purified by silica column chromatography (CH_2Cl_2:MeOH, 99:0 to 19:1 + 1% pyridine) to give dimer **25** (1.12 g, 1.20 mmol, 63%) as a white solid. R_f 0.68 (CH_2Cl_2:MeOH, 19:1); ^{1}H NMR (400 MHz, DMSO-*d*$_6$) δ 11.39 (2 × s, 2H, H-3), 7.86 (d, *J* = 8.0 Hz, 1H, NH CBM), 7.61 – 7.56 (m, 1H, H-6), 7.43 – 7.15 (m, 15H, H-6, H-Ar), 6.91 – 6.82 (m, 4H, H-Ar), 6.17 (t, *J* = 6.3 Hz, 1H, H-1′), 5.53 (s, 1H, H-1″), 4.68 –4.46 (m, 5H, H-2″, H-8, H-9), 4.35 (app p, *J* = 7.4 Hz, 1H, H-3′), 3.97 (s, 1H, H-3″), 3.93–3.90 (m, 2H, H-4′, H-5″), 3.75 (d, 1H, H-5″), 3.71 (2 × s, 6H, OCH_3), 3.27-3.17 (m, 2H, H-5′), 2.43–2.34 (m, 1H, H-2′), 2.26–2.17 (m, 1H, H-2′), 1.74 (d, *J* = 1.1 Hz, 3H, CH_3), 1.49 (d, *J* = 1.2 Hz, 3H, CH_3); ^{13}C NMR (101 MHz, DMSO-*d*$_6$) δ 164.3 (C-4), 164.2 (C-4), 158.6 (C-Ar), 155.7 (C-7), 150.8 (C-2), 150.4 (C-2), 145.2 (C-Ar), 138.2 (C-Ar), 136.3 (C-6), 135.8 (C-Ar), 135.7 (C-Ar), 134.8

(C-6), 130.2 (C-Ar), 128.7 (C-Ar), 128.3 (C-Ar), 128.1 (C-Ar), 127.9 (C-Ar), 127.2 (C-Ar), 113.6 (C-Ar), 110.0 (C-5), 109.2 (C-5), 87.1 (C-1″), 86.2–86.0 (C-4″, C_{quat}DMTr), 84.0 (C-1′), 83.1 (C-4′), 77.2–77.1 (C-2″, C-3″), 72.1 (C-5″), 71.6 (C-8), 63.5 (C-5′), 60.1 (C-9), 55.5–55.4 (OCH_3), 51.1 (C-3′), 37.5 (C-2′), 12.9 (CH_3), 12.3 (CH_3); HRMS–ESI (*m/z*): $[M + Na]^+$ calcd for $C_{50}H_{51}N_5O_{13}Na$, 952.3376 found 952.3379.

5′-*O*-(Dimethoxytrityl)-DNA-CBM$_2$-LNA-thymidine dimer (DNA-CBM$_2$-LNA) **(26)**

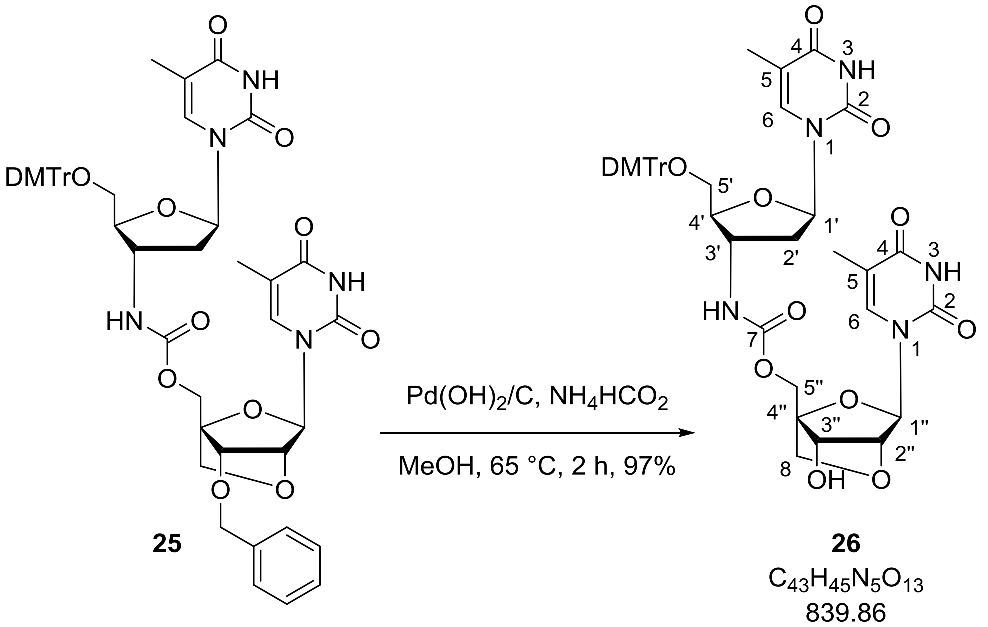

25

26

$C_{43}H_{45}N_5O_{13}$

839.86

To a suspension of dimer **25** (560 mg, 0.602 mmol) and 20% $Pd(OH_2)/C$ (50.0 mg, 360 µmol) in MeOH (50 mL) was added ammonium formate (266 mg, 4.22 mmol) and stirred for 2 h at 65 °C. The reaction was filtered through Celite and concentrated under reduced pressure. The residue was re-dissolved in CH_2Cl_2 (100 mL) and washed with H_2O (3 × 100 mL). The organic phase was dried over Na_2SO_4, filtered and concentrated under reduced pressure to give **26** (490 mg, 0.58 mmol, 97%) as a white solid. R_f 0.12 (CH_2Cl_2:MeOH, 19:1); ^1H NMR (400 MHz, DMSO-d_6) δ 11.36 (s, 2H, H-3), 7.83 (d, *J* = 8.0 Hz, 1H, NH CBM), 7.63 – 7.53 (m, 1H, H-6), 7.45 – 7.35 (m, 3H, H-6, H-Ar), 7.35 – 7.15 (m, 7H, H-Ar), 6.93 – 6.78 (m, 4H, H-Ar), 6.16 (br t, *J* = 6.3 Hz, 1H, H-1'), 5.86 (s, 1H, OH), 5.46 (br s, 1H, H-1''), 4.53 (d, *J* = 12.7 Hz, 1H, H-8), 4.43 (d, *J* = 12.7 Hz, 1H, H-8), 4.35 (t, *J* = 7.4 Hz, 1H, H-3'), 4.17 (s, 1H, H-2''), 3.97 – 3.86 (m, 3H, H-3'', H-5'', H-4')), 3.72 (d, *J* = 1.6 Hz, 6H, OCH_3), 3.69 (d, *J* = 8.1 Hz, 1H, H-5''), 3.29 – 3.14 (m, 2H, H-5'), 2.38 (m, 1H, H-2'), 2.21 (m, 1H, H-2'), 1.75 (d, *J* = 1.1 Hz, 3H, CH_3), 1.49 (d, *J* = 1.1 Hz, 3H, CH_3); ^{13}C NMR (101 MHz, DMSO-d_6) δ 164.3 (C-4), 164.2 (C-4), 158.6 (C-Ar), 158.6 (C-Ar), 155.8 (C-7), 150.8 (C-2), 150.4 (C-2), 145.2 (C-Ar), 136.3 (C-6), 135.8-134.9 (C-6, 3 × C-Ar), 128.3 (C-Ar), 128.1, (C-Ar) 127.2 (C-Ar), 113.6 (C-Ar), 110.0 (C-5), 109.2 (C-5), 86.9 (C_{quat}DMTr), 86.8 (C-1″), 86.2 (C-4″), 84.0 (C-1′), 83.0 (C-4′), 79.6 (C-2″), 71.4 (C-5″), 70.2 (C-3″), 63.5 (C-5′), 60.4 (C-8), 55.4 (OCH_3), 51.0 (C-3′), 37.5 (C-2′), 12.7 (CH_3), 12.3 (CH_3); HRMS–ESI (*m/z*): $[M + Na]^+$ calcd for $C_{43}H_{45}N_5O_{13}Na$, 862.2906 found 862.2909.

5′-*O*-(Dimethoxytrityl)-DNA-CBM$_2$-LNA thymidine phosphoramidite dimer (DNA-CBM$_2$-LNA) **(27)**

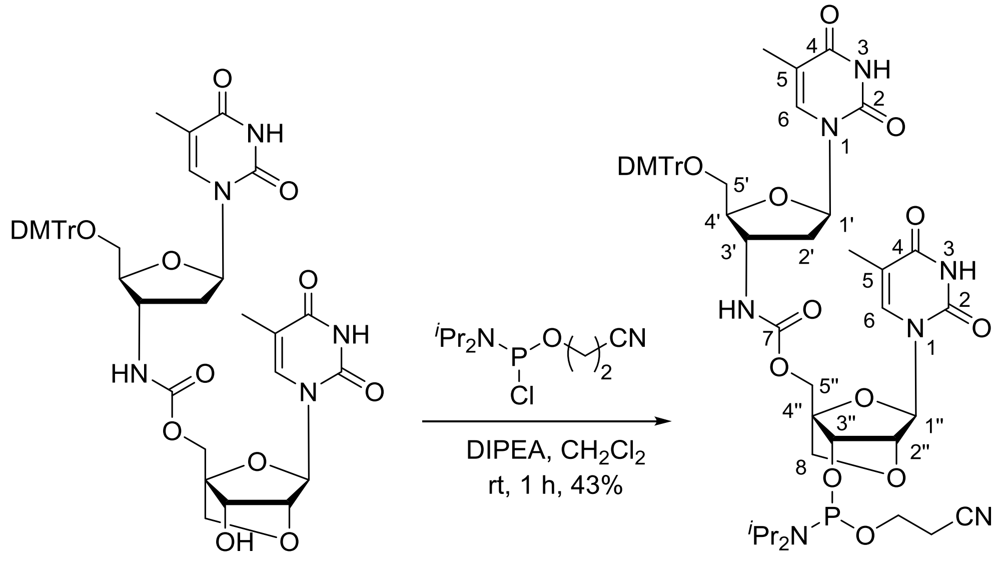

26

27
$C_{52}H_{62}N_7O_{14}P$
1040.08

To a solution of dimer **26** (490 mg, 580 µmol) in dry degassed CH_2Cl_2 (5.00 mL) was added dry degassed DIPEA (254 µL, 1.50 mmol) followed by chloro(diisopropylamino)-*β*-cyanoethoxyphosphine (196 µL, 880 µmol) and stirred at rt under argon for 1 h. The reaction was diluted with degassed CH_2Cl_2 (5.00 mL), washed with sat. aq KCl (5.00 mL) and dried over Na_2SO_4. The organic layer was concentrated under reduced pressure and purified by silica column chromatography (degassed EtOAc) to give phosphoramidite **27** (260 mg, 250 µmol 43%). R_f 0.56 (EtOAc); ^{31}P NMR (162 MHz) 149.1, 147.8; HRMS–ESI (*m/z*): [M + Na]$^+$ calcd for $C_{52}H_{62}N_7O_{14}PNa$ 1062.3985 found 1062.3975

Synthesis and purification of oligonucleotides

Synthesis of DNA oligonucleotides

Standard phosphoramidites, solid supports and reagents were purchased from Link Technologies and Applied Biosystems. LNA phosphoramidites were obtained from Exiqon. Automated solid phase synthesis of oligonucleotides (trityl off) was performed on an Applied Biosystems 394 synthesiser. Synthesis was performed on 1.0 µmole scale involving cycles of acid catalysed detritylation, coupling, capping, and iodine oxidation. Standard DNA phosphoramidites were coupled for 60 s while extended coupling time of 10 min was used for LNA phosphoramidites. Coupling efficiencies and overall synthesis yields were determined by the inbuilt automated trityl cation conductivity monitoring facility and were ≥98.0% in all cases. The oligonucleotides were then cleaved from the solid support and protecting groups from the nucleobase and backbone were removed by exposure to conc. aq ammonium hydroxide for 60 min at room temperature, followed by heating in a sealed tube for 5 h at 55 °C.

Synthesis of RNA oligonucleotides

2′-*O*-TBDMS protected RNA phosphoramidite monomers with *t*-butylphenoxyacetyl protection of the A, G and C nucleobases were used to assemble RNA oligonucleotides. Benzylthiotetrazole (BTT) was used as the coupling agent, *t*-butylphenoxyacetic anhydride as the capping agent and 0.1 M iodine as the oxidizing agent (Sigma-Aldrich). Coupling time of 10 min was used and coupling efficiencies of >97% were obtained. Cleavage of oligonucleotides from the solid support and protecting groups from the nucleobase and backbone were removed by exposure to concentrated aqueous ammonia/ethanol (3/1 v/v) for 2 h at room temperature followed by heating in a sealed tube for 2 h at 55°C.

Removal of 2′-*O*-TBDMS protecting groups on RNA oligonucleotides

Oligonucleotides were concentrated under reduced pressure until formation of a white turbidity and freeze dried overnight. The residue was then re-dissolved in DMSO (300 µL) and triethylamine trihydrofluoride (300 µL) was added followed by incubation at 65 °C for 2.5 h. Sodium acetate (3 M, 50 µL) and butanol (3 mL) were added and the samples were stored at –80 °C for a minimum of 1 h. After thawing the suspension was pelleted under centrifuge (13,000 rpm, 4 °C, 10 min) and the supernatant decanted. The precipitate was then washed with ethanol (2 × 750 µL) then dried under vacuum.

Purification of oligonucleotides

Oligonucleotides were purified using high performace liquid chromatography (HPLC) performed on a Gilson 118 UV detector, with 306 pumps, 805 manometric module, and 811c dynamic mixer, equipped with a Phenomenex Luna 10 µm C8 column (100 Å pore; 10 × 250 mm). A gradient of acetonitrile in 0.1 M TEAB buffer (from 10:90 to 40:60 acetonitrile:TEAB) was used to elute the ONs at rt over 20 mins at a flow rate of 4 mL/min. Elution was monitored by UV absorbance at 285–300 nm. Collected fractions were freeze-dried overnight and re-suspended in 1 mL deionised water.

Table S1. List of modified oligonucleotides with calculated and found mass spectrometry data, x = CBM_1, y = CBM_2, ^{L} = LNA.

ON Code	Sequence / 5′-3′	Calculated /Da	Found /Da
ON2	GCTTGCT**x**TCGTTCC	4160	4160
ON3	GCTTGCT^L**x**TCGTTCC	4188	4187
ON4	GCTTGCT**x**T^{L}CGTTCC	4188	4188
ON5	GCTTGCT^L**x**T^{L}CGTTCC	4216	4215
ON6	GCTTGCT**y**TCGTTCC	4160	4158
ON7	GCTTGCT**y**T^{L}CGTTCC	4188	4188
ON8	GCT**x**TGCT**x**TCGT**x**TCC	4186	4186
ON9	GCT^L**x**TGCT^L**x**TCGT^L**x**TCC	4170	4167
ON10	GCT**x**T^{L}GCT**x**T^{L}CGT**x**T^{L}CC	4170	4169
ON11	GCT^L**x**T^{L}GCT^L**x**T^{L}CGT^L**x**T^{L}CC	4254	4255
ON12	GCT**y**TGCT**y**TCGT**y**TCC	4086	4085
ON13	GCT**y**T^{L}GCT**y**T^{L}CGT**y**T^{L}CC	4170	4170
ON14	GCTT^{L}GCTT^{L}CGTT^{L}CC	4281	4280
ON15	GCT^{L}T^{L}GCT^{L}T^{L}CGT^{L}T^{L}CC	4365	4365

Biophysical studies and assays

Ultraviolet melting studies

UV DNA melting curves were recorded in a Cary 4000 Scan UV-Visible Spectrophotometer using 3 µM of each oligonucleotide in a 10 mM phosphate buffer containing 200 mM NaCl at pH 7.0. Samples were annealed by heating to 85 °C (10 °C/min) and then slowly cooling to 20 °C (1 °C/min). As these six successive cycles (heating and cooling) were performed at a gradient of 1 °C/min, the change in UV absorbance at 260 nm was recorded. The melting temperature was calculated from the first derivative of the melting curve using in built software.

Additional UV-data

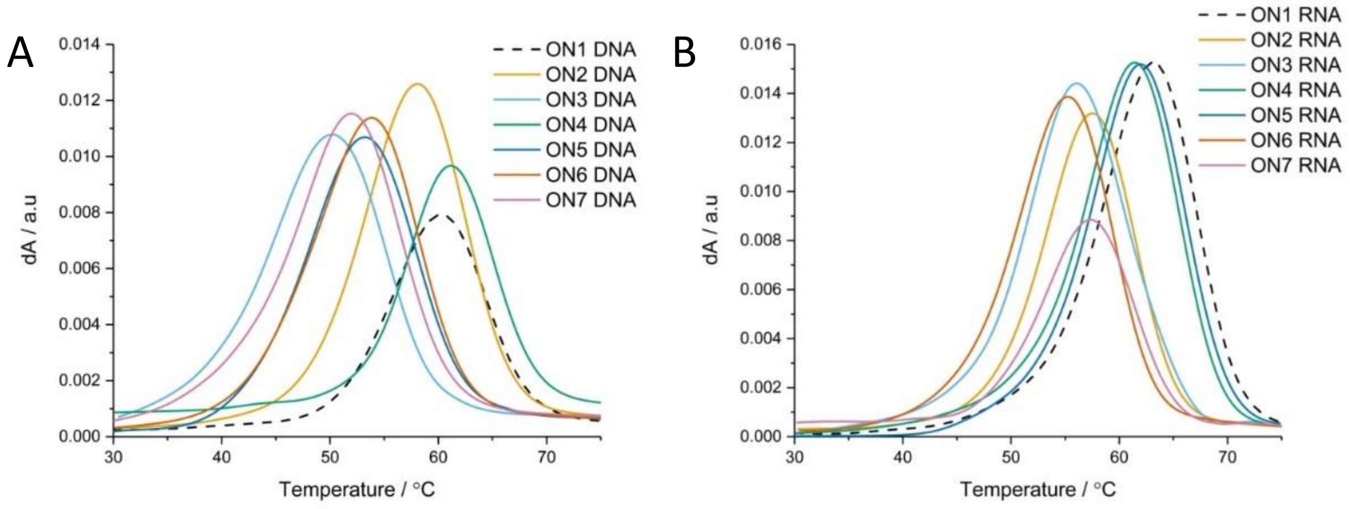

Figure S1. First derivatives of UV-melting curves containing 3 µM oligonucleotide with single modified carbamate backbones in 10 mM phosphate, 200 mM NaCl, pH 7.0 buffer. Samples were taken as an average of six ramps from 20 to 85 °C. (A) Modifications in DNA duplex single addition, (B) modifications in DNA:RNA hybrid single addition.

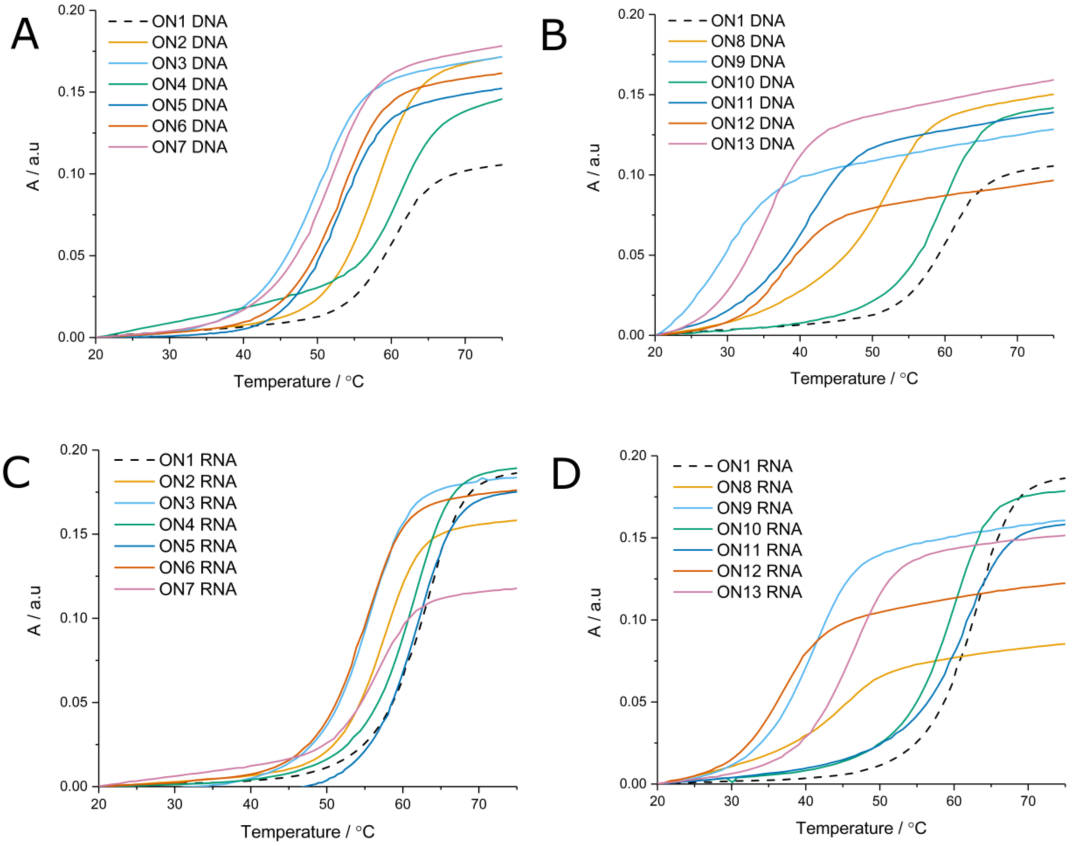

Figure S2. UV-melting curves of CBM-LNA modified oligonucleotides containing 3 μM oligonucleotide in 10 mM phosphate, 200 mM NaCl, pH 7.0 buffer. Samples were taken as an average of six ramps from 20 to 85 °C. (A) single incorporation of backbone modifications in DNA duplex, (B) triple incorporation of backbone modifications in DNA duplex, (C) single incorporation of backbone modifications in DNA:RNA hybrid, (D) triple incorporation of backbone modifications in DNA:RNA hybrid.

Circular dichroism studies

A solution of 3 μM sample oligonucleotide and complementary sequence was added to 10 mM phosphate buffer with 200 mM NaCl (pH 7.0). Sample were incubated for 2 mins at 85 °C and cooled to rt. Circular dichroisms was performed on a Chirscan Plus spectrometer using a quartz cuvette (L = 10 mm). Scans were taken at 20 °C from 200–340 nm with 0.5 a step and 1.0 s time point intervals. The average of four scan were taken and smoothed to 20 points using a third order polynomial (OriginPro 2017). The spectra were then baseline corrected to the θ-value at 340 nm.

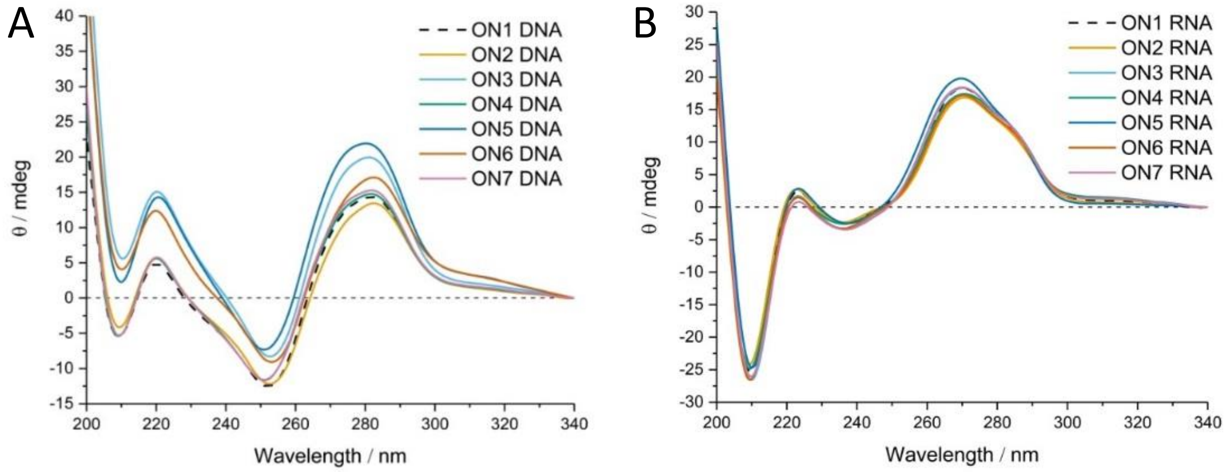

Figure S3. Circular dichroism of 3 µM sample oligonucleotides in 10 mM phosphate, 200 nM NaCl pH = 7.0 buffer. Spectra were averaged from four scans and smoothed to 20 points using a third order polynomial. (A) Single incorporation of CBM-LNA modifications against DNA target, (B) Single incorporation of CBM-LNA modifications against RNA target.

Snake venom assay

5 nmol of oligonucleotide was dissolved in 100 µL of buffer (50 mM Tris-HCl, 10 mM $MgCl_2$, pH 9.0). 20 µL of sample was removed (t = 0 control) and 2 µL of snake venom phosphodiesterase 1 from *C. adamanteus* (Sigma-Aldrich, cat P3243, 0.45 units, dissolved in 7 mL H_2O) was added to the remaining volume. The reaction was incubated at 37 °C and 20 µL aliquots were removed at set times, mixed with formamide (v/v) and stored at -80 °C. Samples were analysed by 20% denatured polyacrylamide gel electrophoresis (400 V) and viewed under short wave UV-light.

Foetal bovine serum (FBS) assay

5 nmol of oligonucleotide was dissolved in 50 µL of Dulbecco's PBS and 50 µL of FBS (Gibco, standard sterile filtered). This was then vortexed and 20 µL was removed (t = 0 control), mixed with formamide (v/v) and frozen at –80 °C. The remaining solution was incubated at 37 °C and 20 µL aliquots were removed at set times, mixed with formamide (v/v) and stored at –80 °C. Samples were analysed by 20% denatured polyacrylamide gel electrophoresis (400 V) and viewed under short wave UV-light.

Gel quantification

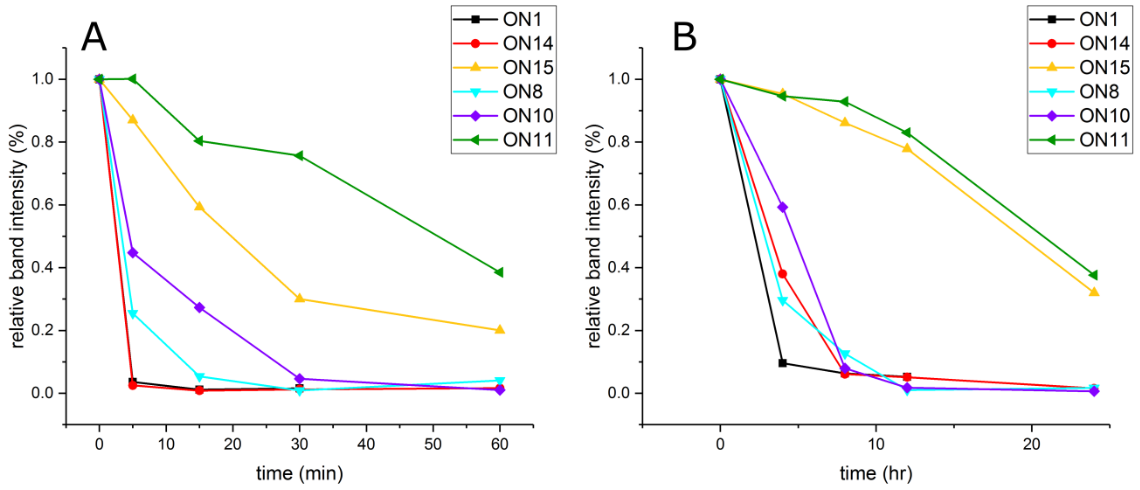

Figure S4. Enzymatic stability of ONs shown in Figure 3 in (A) snake venom and (B) foetal bovine serum. Data points were acquired from PAGE gels (Figure 3) by creating a plot profile for each lane and successive integration of the area under the curve from band corresponding to the highest molecular weight ONs using ImageJ. The intensities are presented as percentage in relation to *t*=0 controls.

HPLC spectra

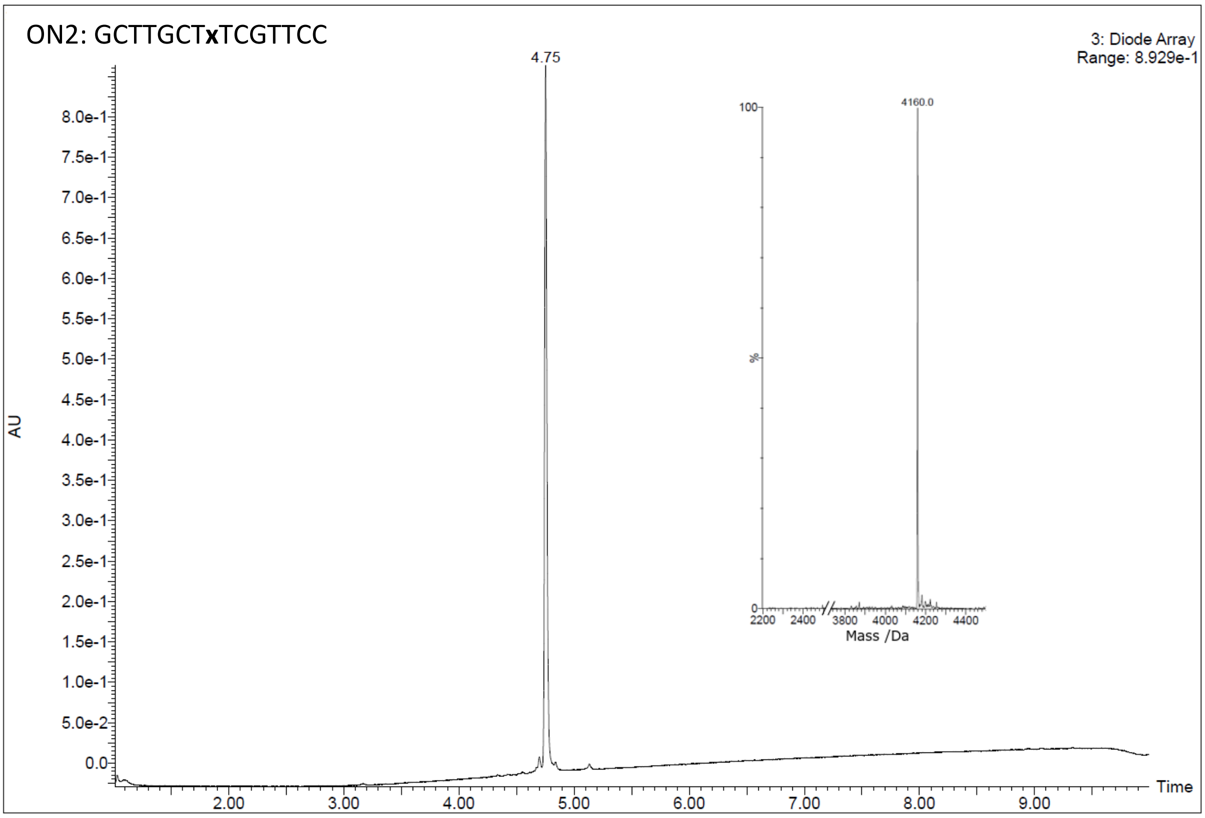

ON3: GCTTGCT^{L}**x**TCGTTCC

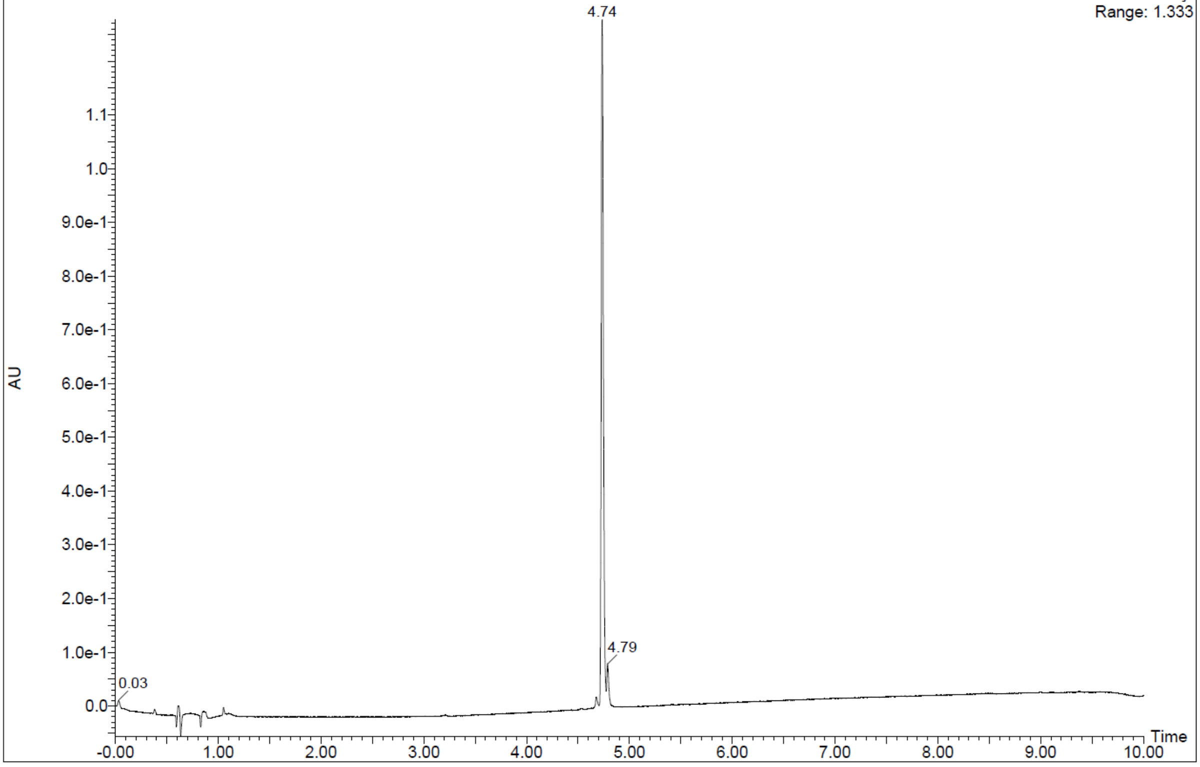

ON4: GCTTGCT**x**T^{L}CGTTCC

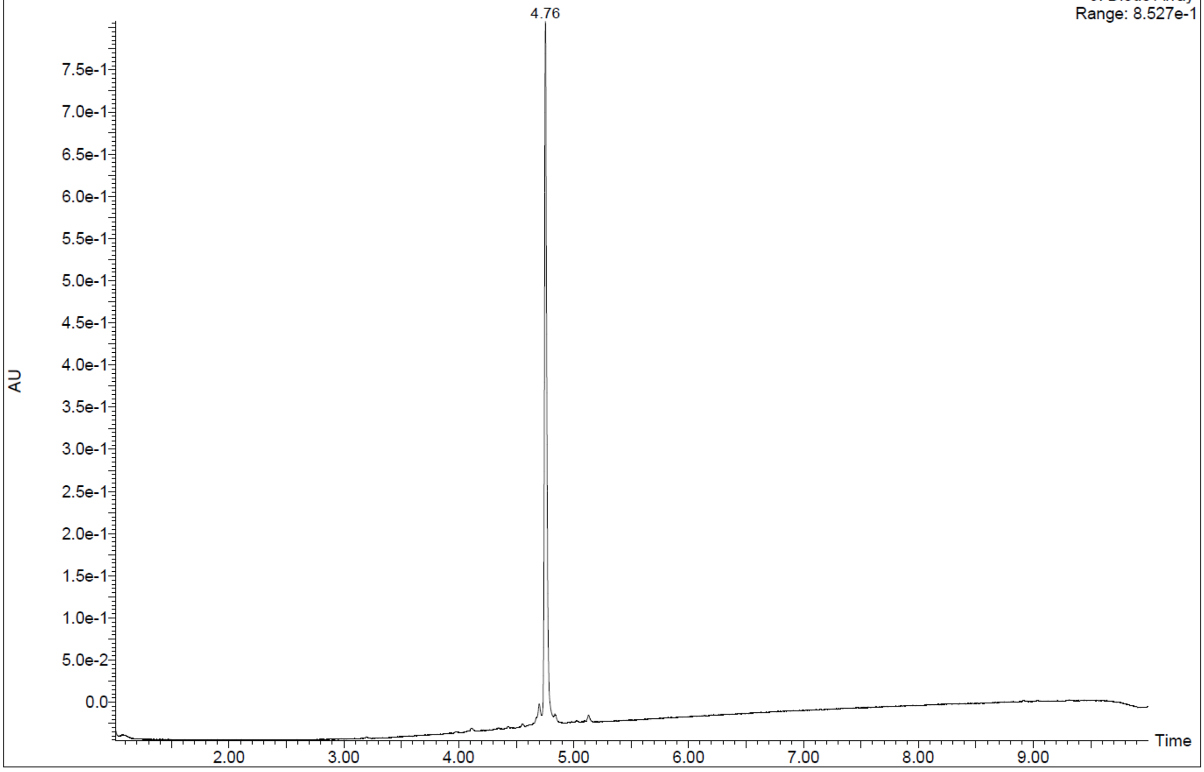

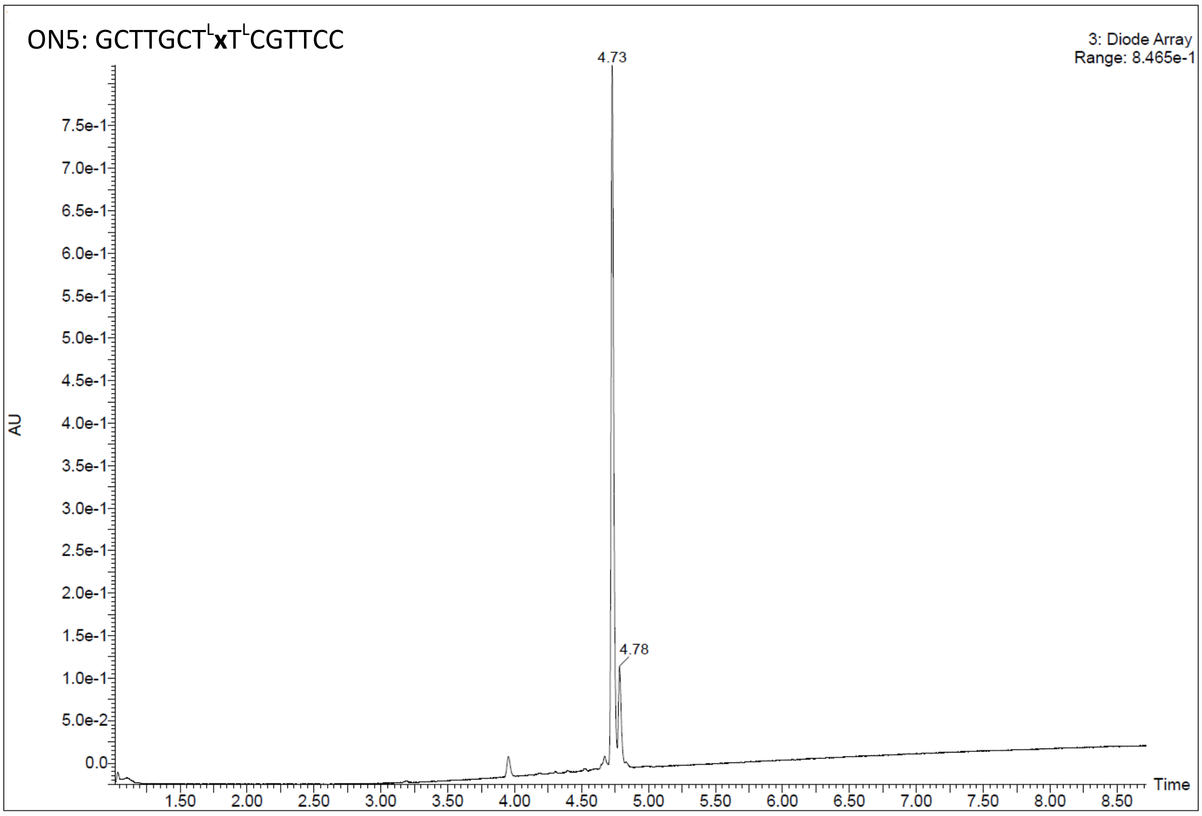
ON5: GCTTGCT^L**x**T^LCGTTCC
3: Diode Array
Range: 8.465e-1
4.73
4.78
AU
Time
7.5e-1
7.0e-1
6.5e-1
6.0e-1
5.5e-1
5.0e-1
4.5e-1
4.0e-1
3.5e-1
3.0e-1
2.5e-1
2.0e-1
1.5e-1
1.0e-1
5.0e-2
0.0
1.50
2.00
2.50
3.00
3.50
4.00
4.50
5.00
5.50
6.00
6.50
7.00
7.50
8.00
8.50

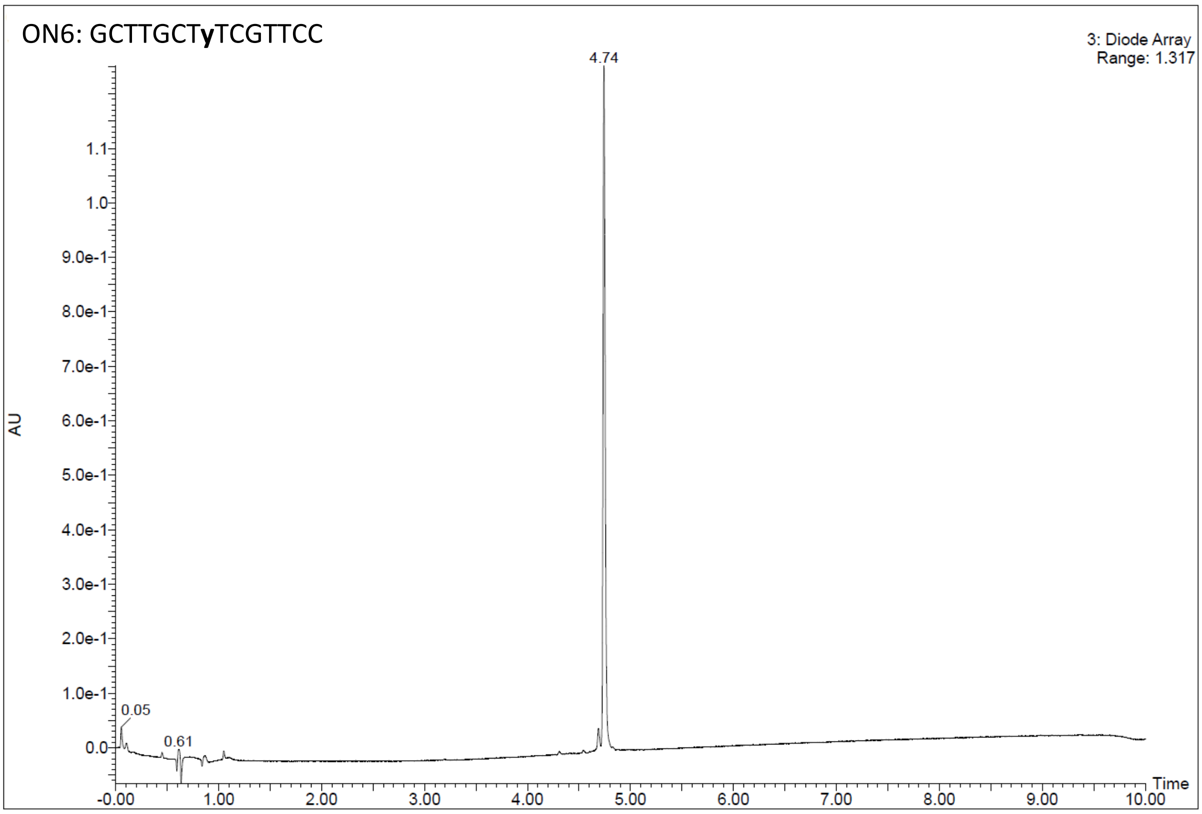
ON6: GCTTGCT**y**TCGTTCC
3: Diode Array
Range: 1.317
4.74
0.05
0.61
AU
Time
1.1
1.0
9.0e-1
8.0e-1
7.0e-1
6.0e-1
5.0e-1
4.0e-1
3.0e-1
2.0e-1
1.0e-1
0.0
-0.00
1.00
2.00
3.00
4.00
5.00
6.00
7.00
8.00
9.00
10.00

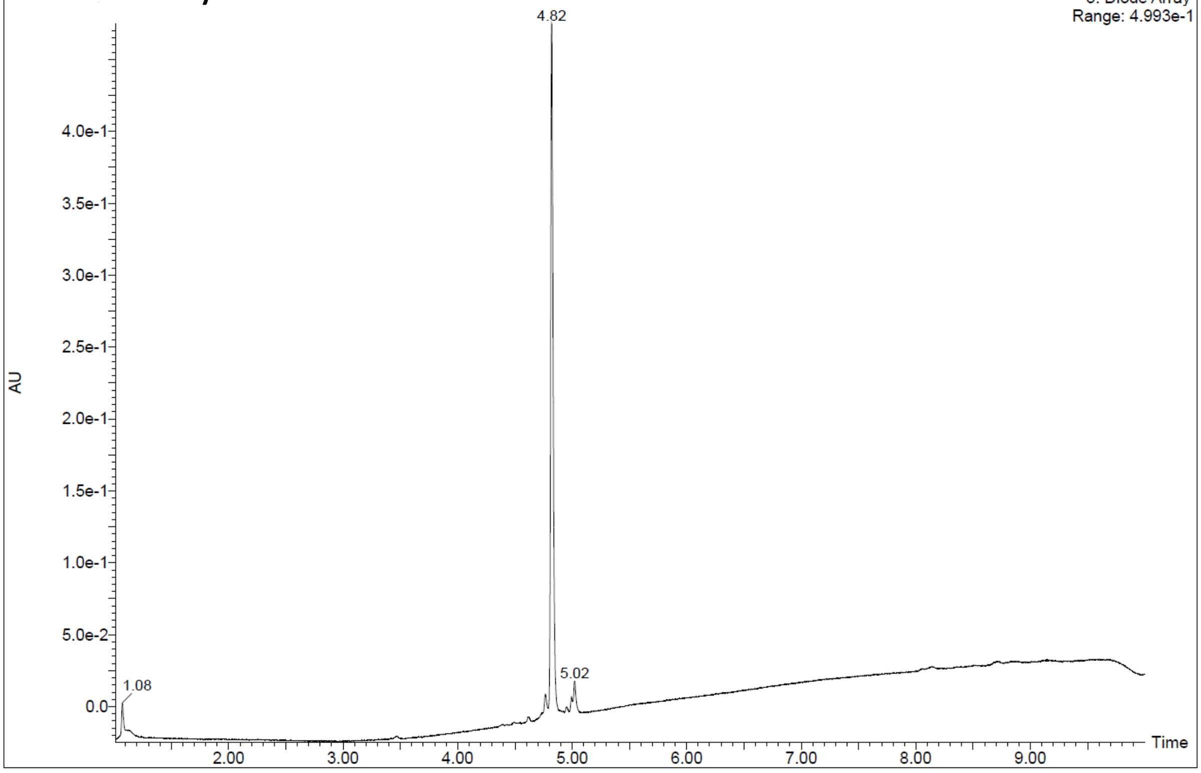

ON7: GCTTGCT**y**T[L]CGTTCC

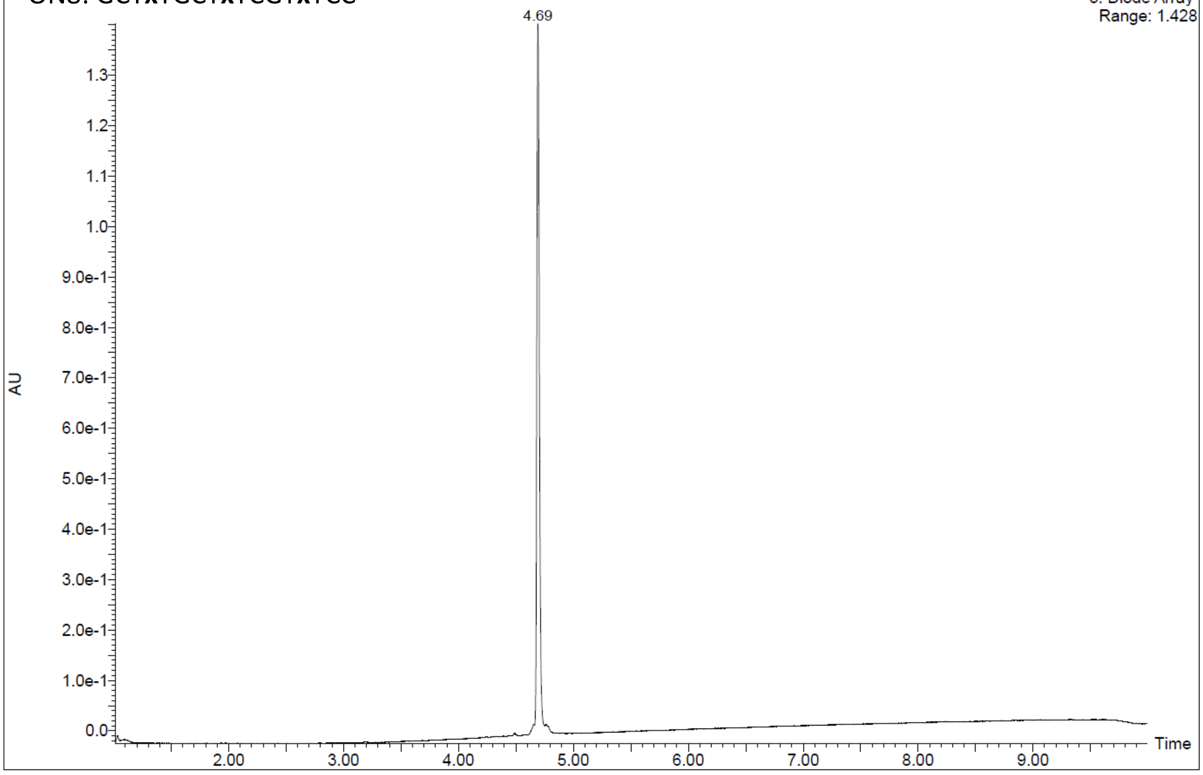

ON8: GCT**x**TGCT**x**TCGT**x**TCC

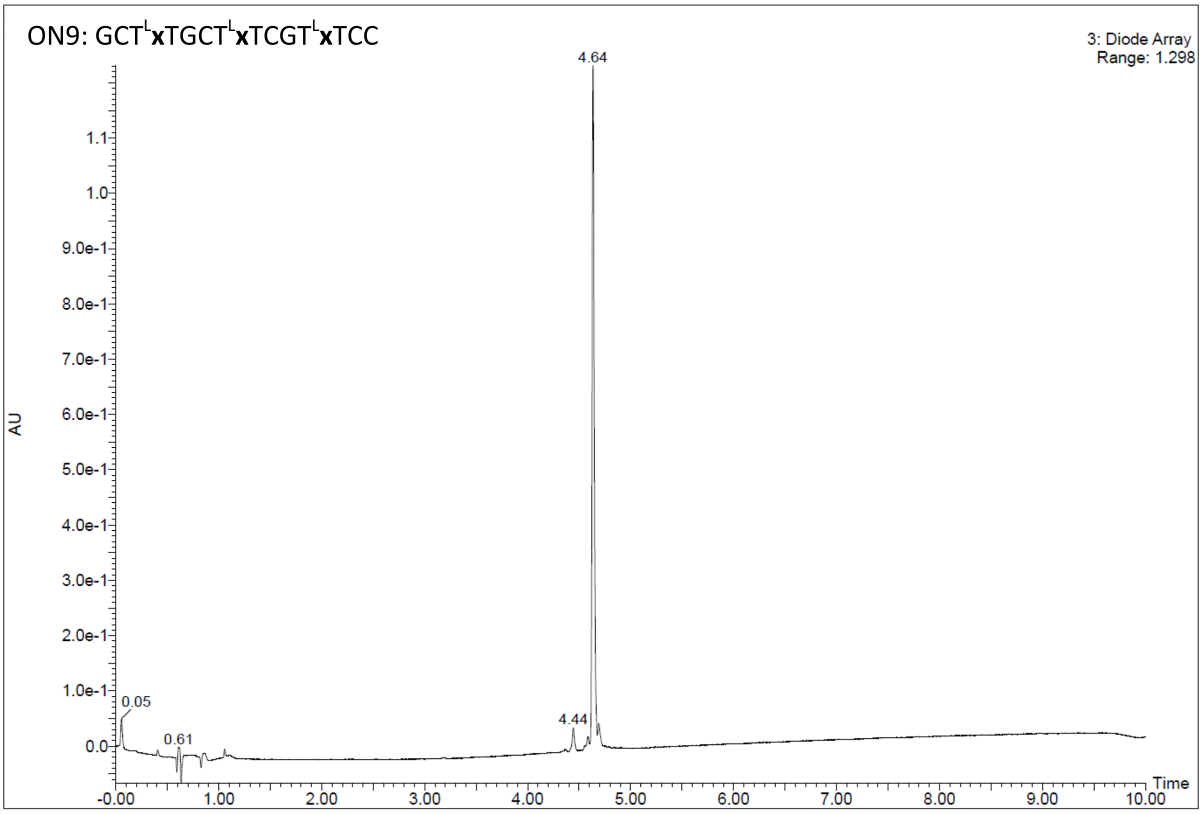
ON9: GCT^LxTGCT^LxTCGT^LxTCC
3: Diode Array
Range: 1.298
4.64
4.44
0.05
0.61
AU
Time

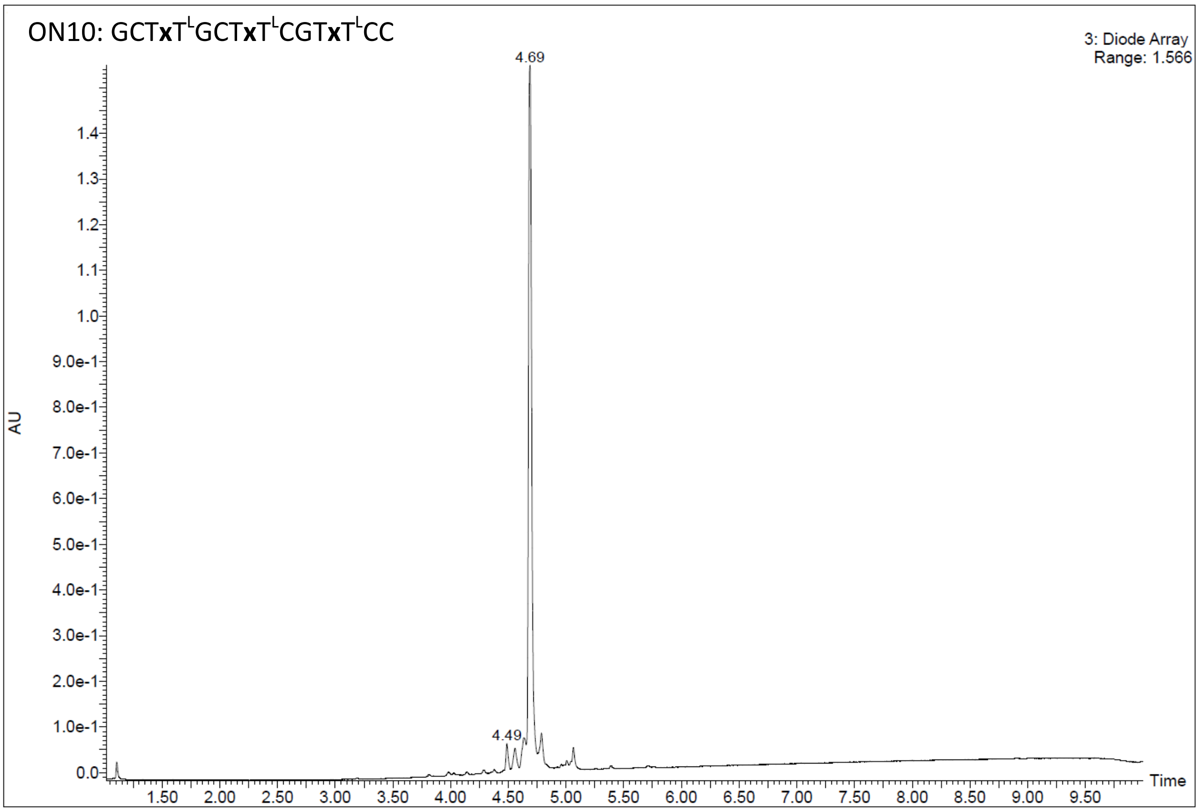
ON10: GCTxT^LGCTxT^LCGTxT^LCC
3: Diode Array
Range: 1.566
4.69
4.49
AU
Time

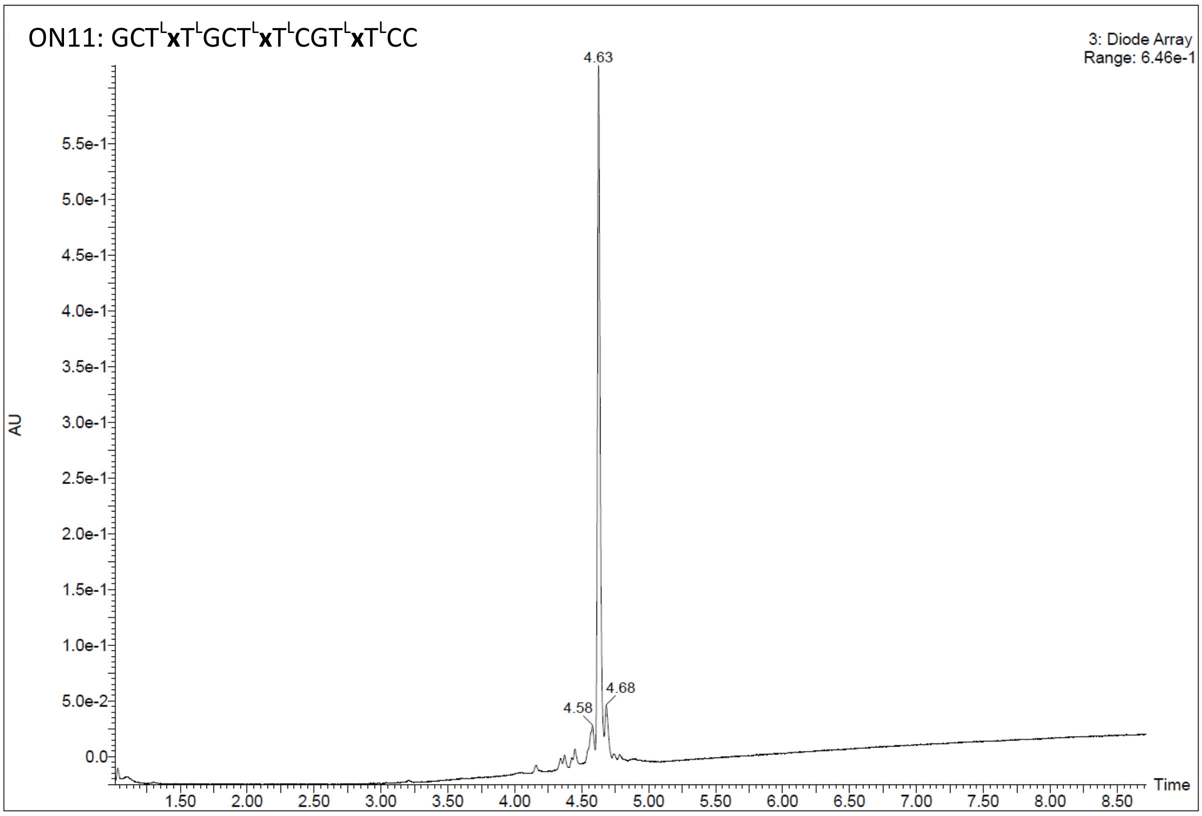
ON11: GCT^L**x**T^LGCT^L**x**T^LCGT^L**x**T^LCC
3: Diode Array
Range: 6.46e-1
4.63
4.58
4.68
AU
Time

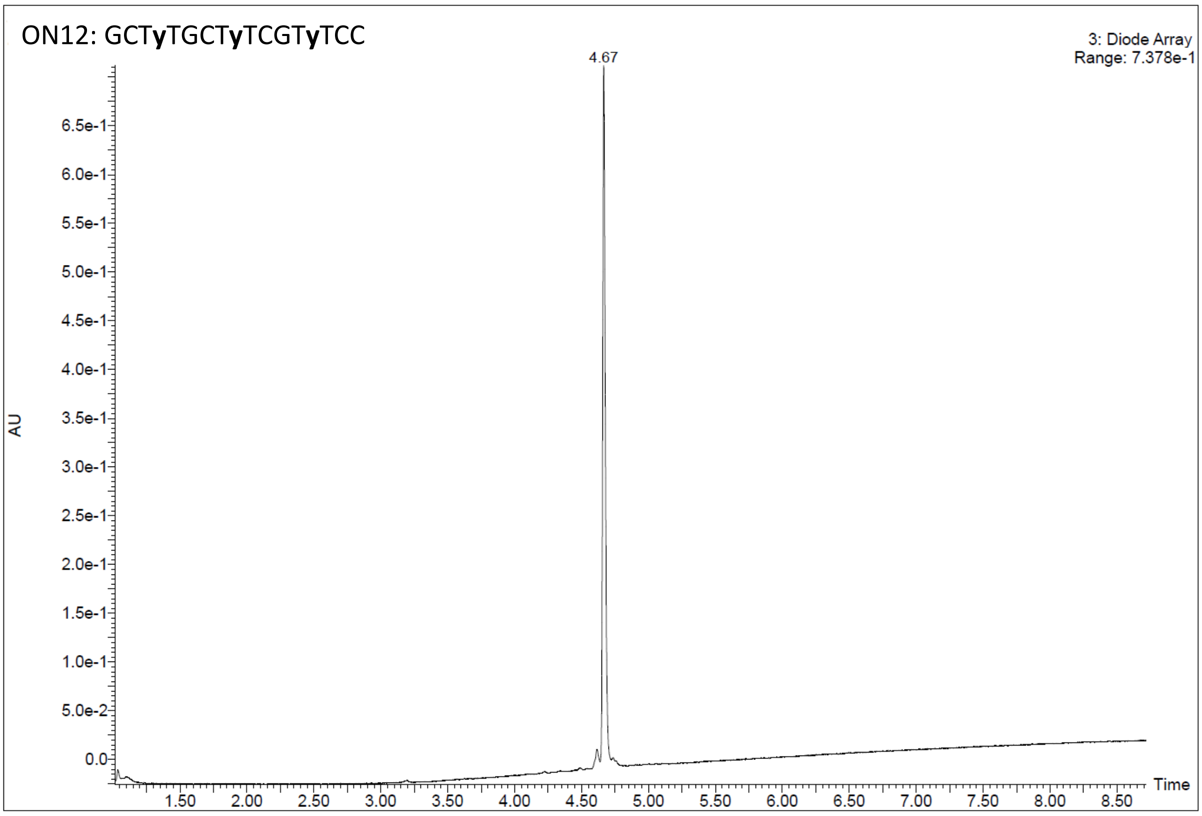
ON12: GCT**y**TGCT**y**TCGT**y**TCC
3: Diode Array
Range: 7.378e-1
4.67
AU
Time

ON13: GCT**y**T^{L}GCT**y**T^{L}CGT**y**T^{L}CC

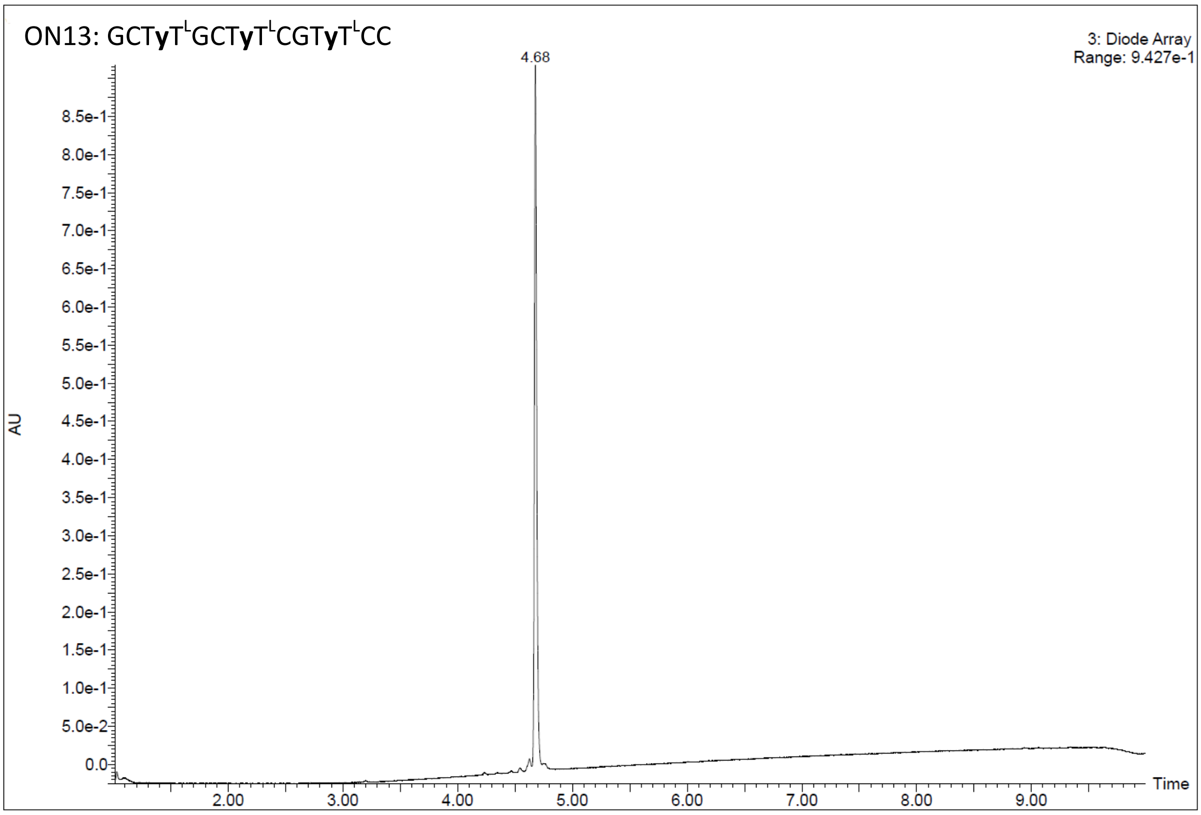

ON14: GCTT^{L}GCTT^{L}CGTT^{L}CC

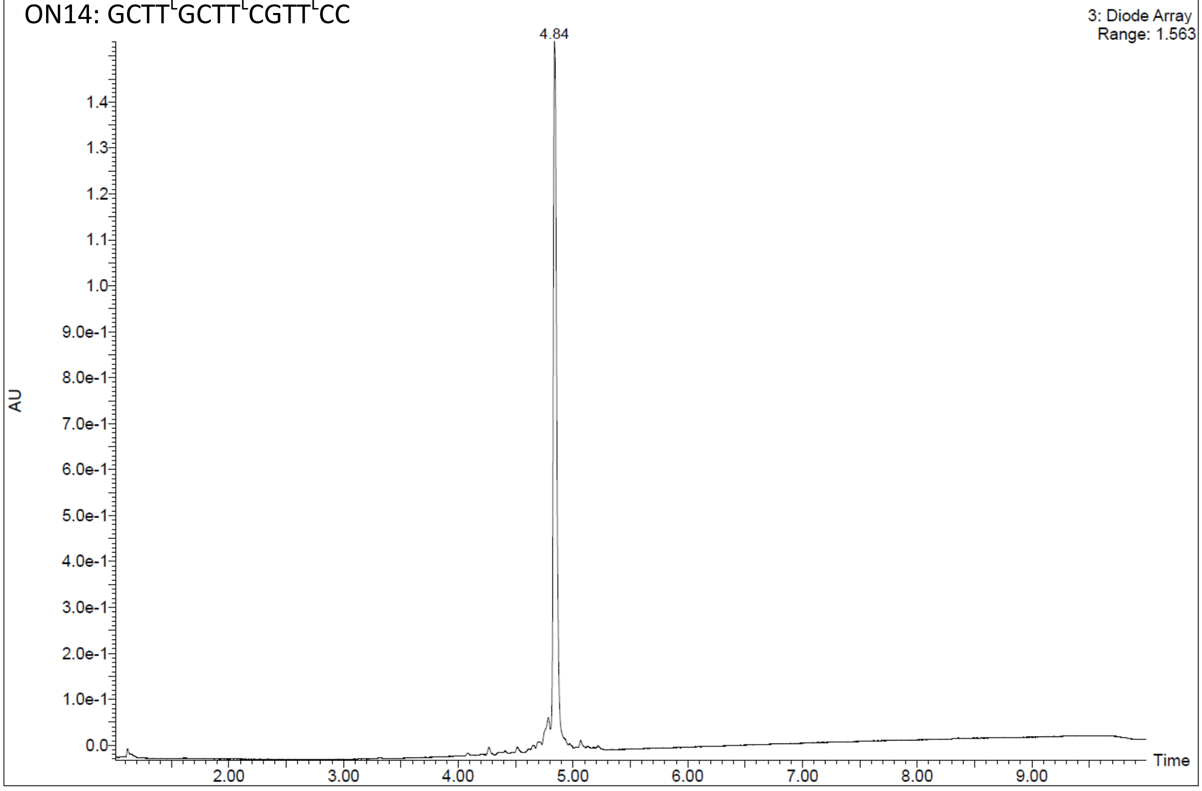

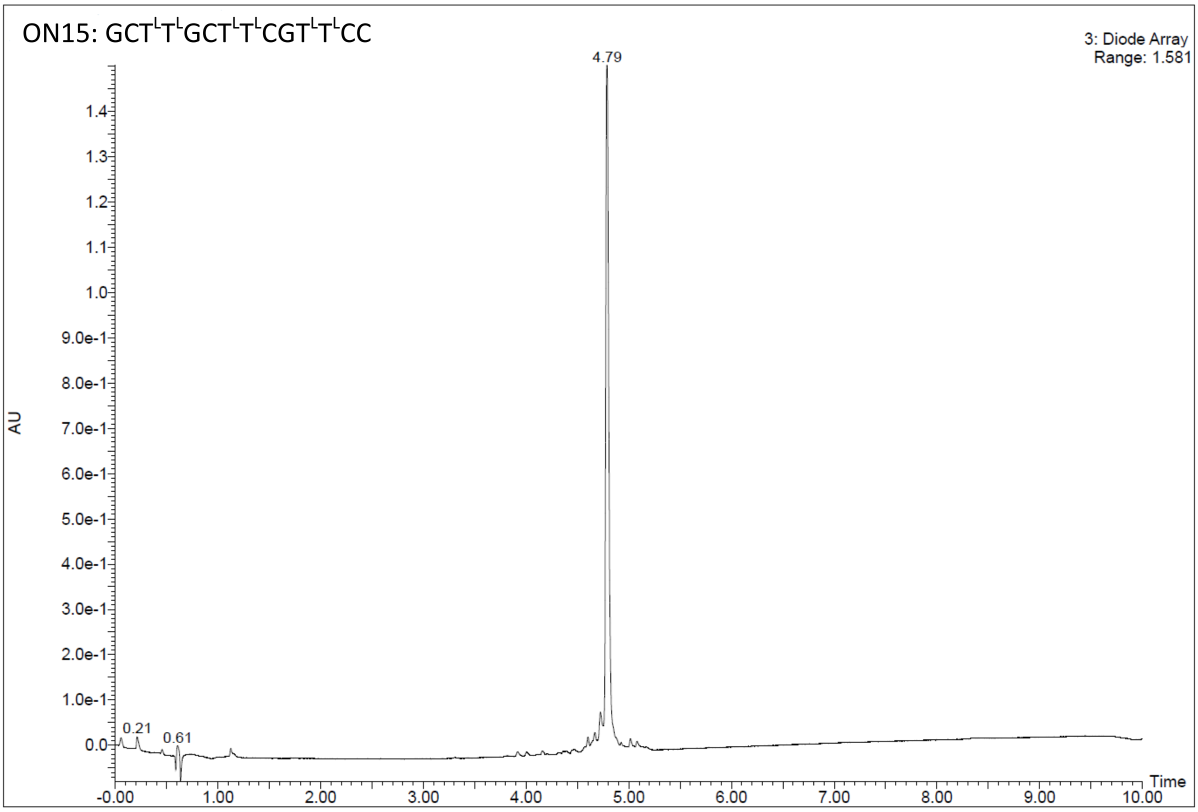
ON15: GCT^L^T^L^GCT^L^T^L^CGT^L^T^L^CC
3: Diode Array
Range: 1.581
4.79
0.21
0.61
AU
Time
1.4
1.3
1.2
1.1
1.0
9.0e-1
8.0e-1
7.0e-1
6.0e-1
5.0e-1
4.0e-1
3.0e-1
2.0e-1
1.0e-1
0.0
-0.00
1.00
2.00
3.00
4.00
5.00
6.00
7.00
8.00
9.00
10.00

NMR spectra

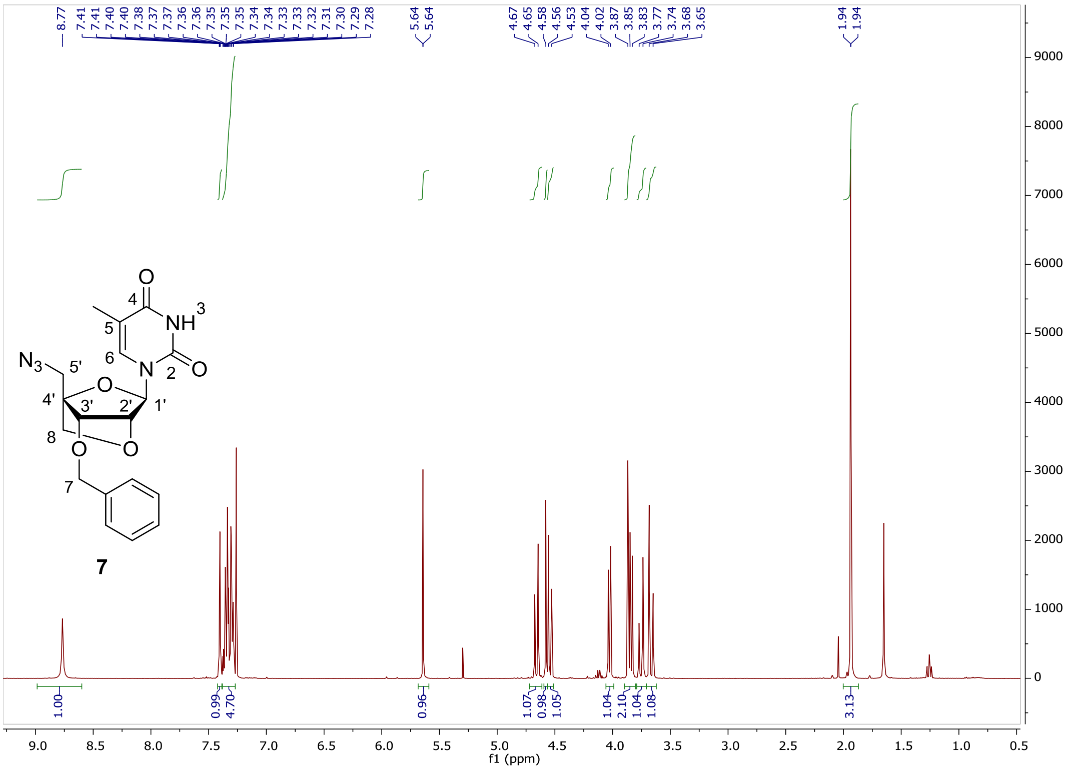

8.77
7.41
7.40
7.40
7.38
7.37
7.37
7.36
7.36
7.35
7.35
7.35
7.34
7.34
7.33
7.33
7.32
7.31
7.30
7.29
7.28
5.64
5.64
4.67
4.65
4.58
4.56
4.53
4.04
4.02
3.87
3.85
3.83
3.77
3.74
3.68
3.65
1.94
1.94
1.00
0.99
4.70
0.96
1.07
0.98
1.05
1.04
2.10
1.04
1.08
3.13
f1 (ppm)
7

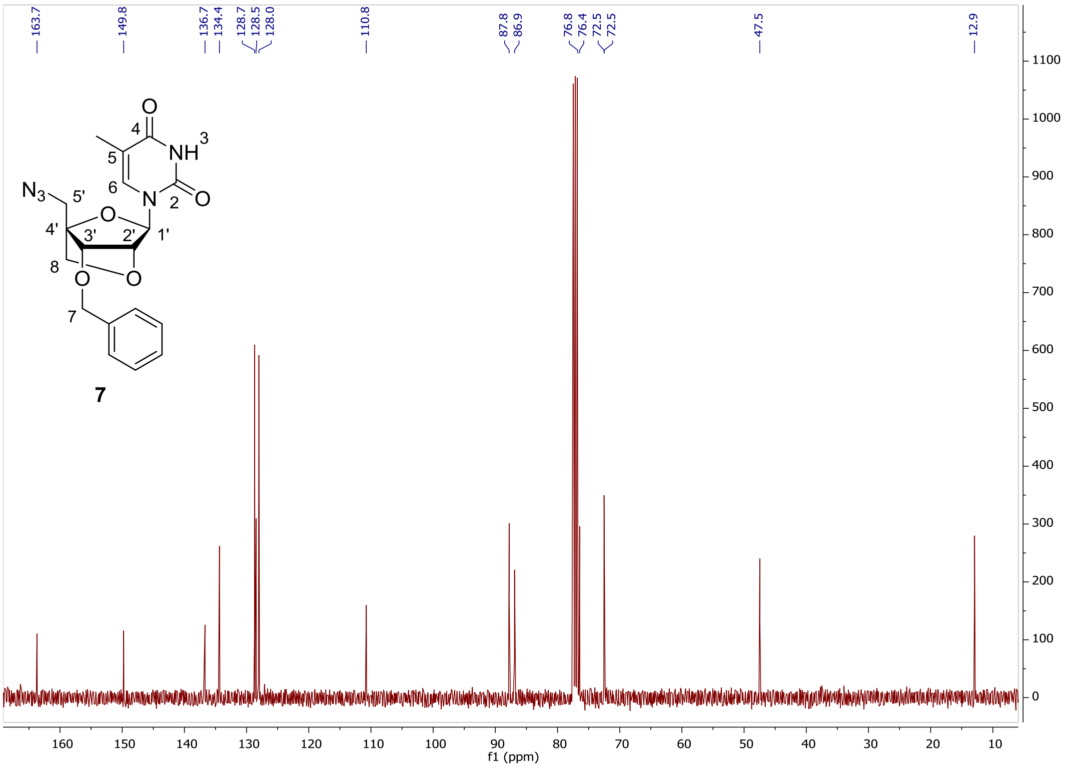

163.7
149.8
136.7
134.4
128.7
128.5
128.0
110.8
87.8
86.9
76.8
76.4
72.5
72.5
47.5
12.9
f1 (ppm)
7

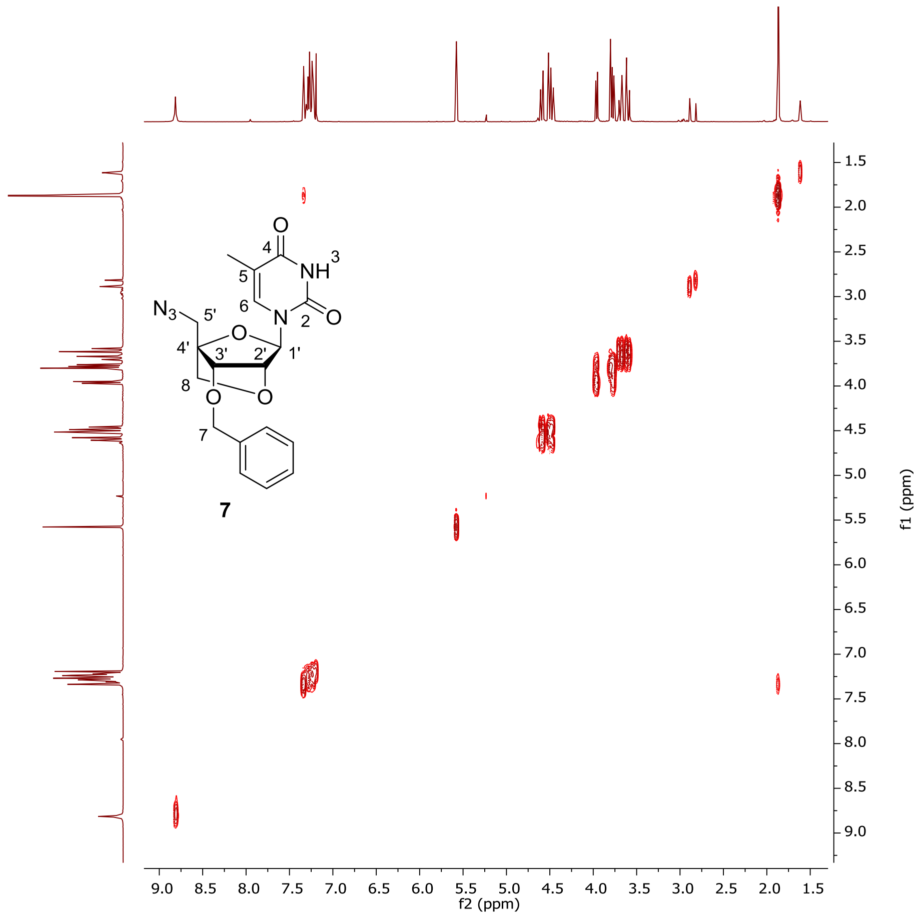

N3
O
NH
5'
4'
3'
2'
1'
8
7
7
f2 (ppm)
f1 (ppm)

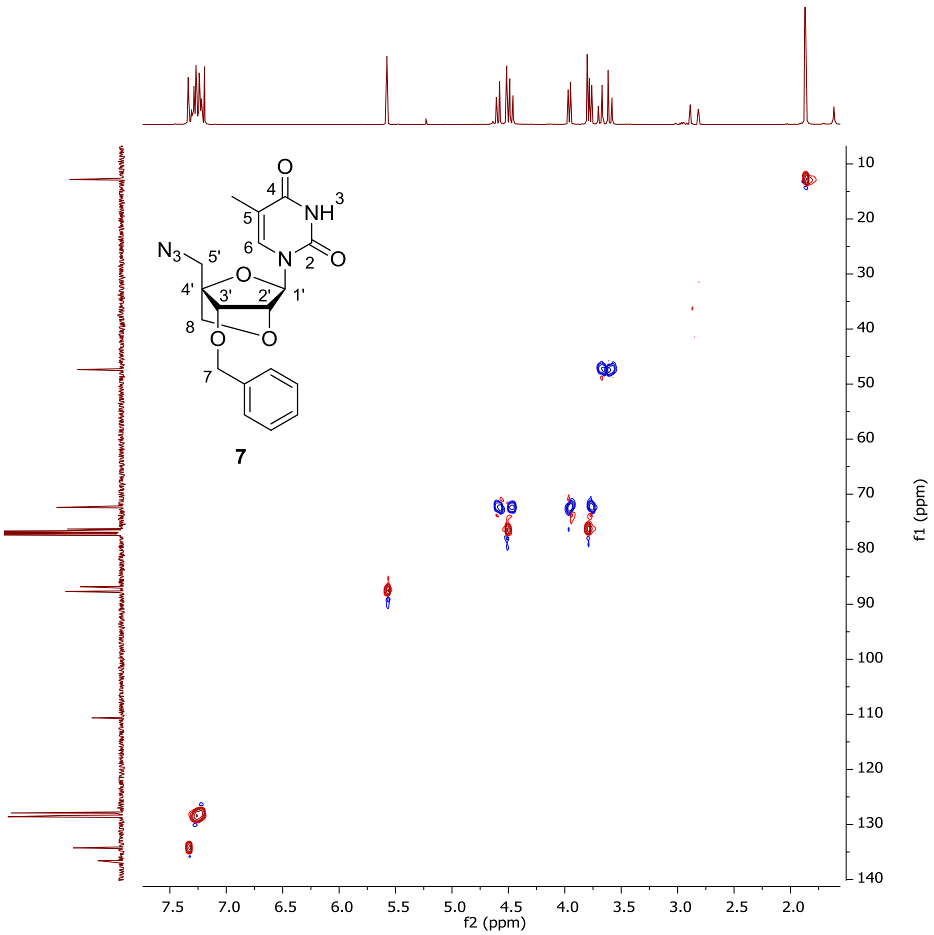

N3
O
NH
5'
4'
3'
2'
1'
8
7
7
f2 (ppm)
f1 (ppm)

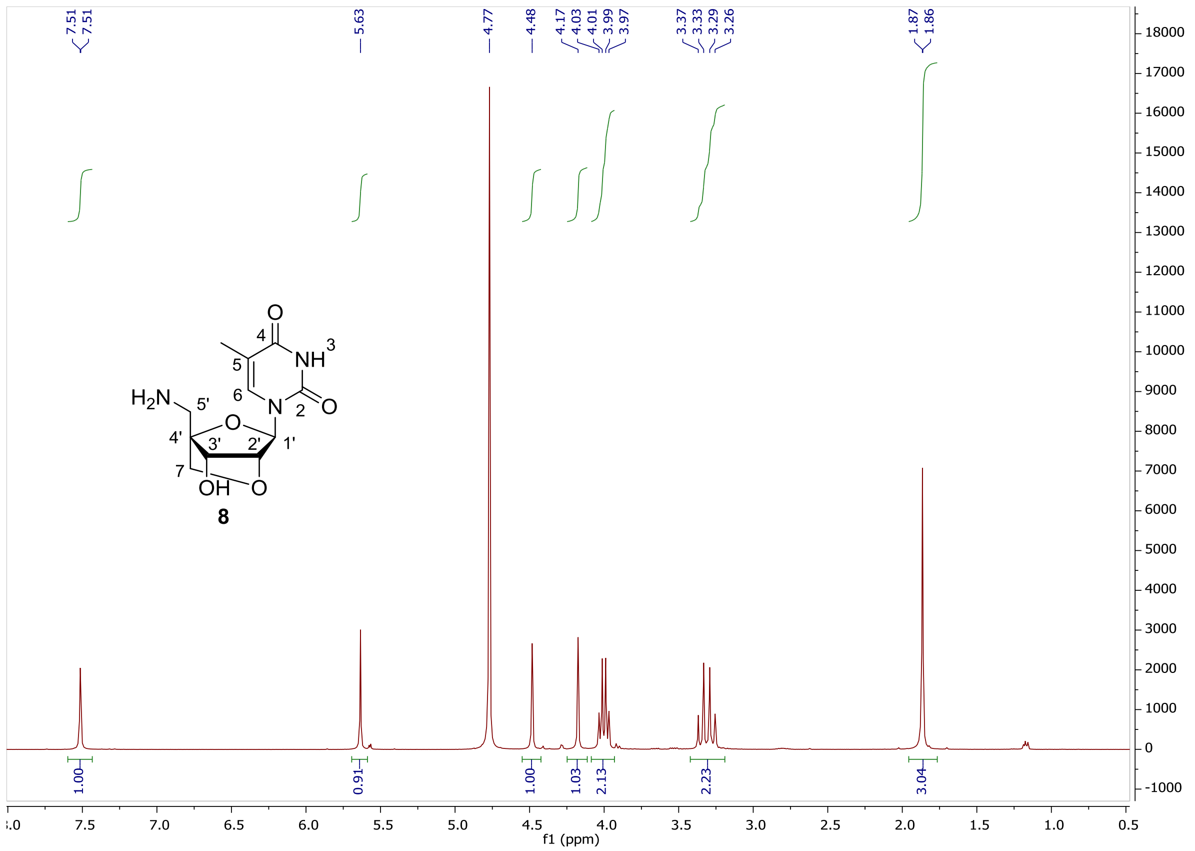
7.51
7.51
5.63
4.77
4.48
4.17
4.03
4.01
3.99
3.97
3.37
3.33
3.29
3.26
1.87
1.86
1.00
0.91
1.00
1.03
2.13
2.23
3.04
f1 (ppm)
8

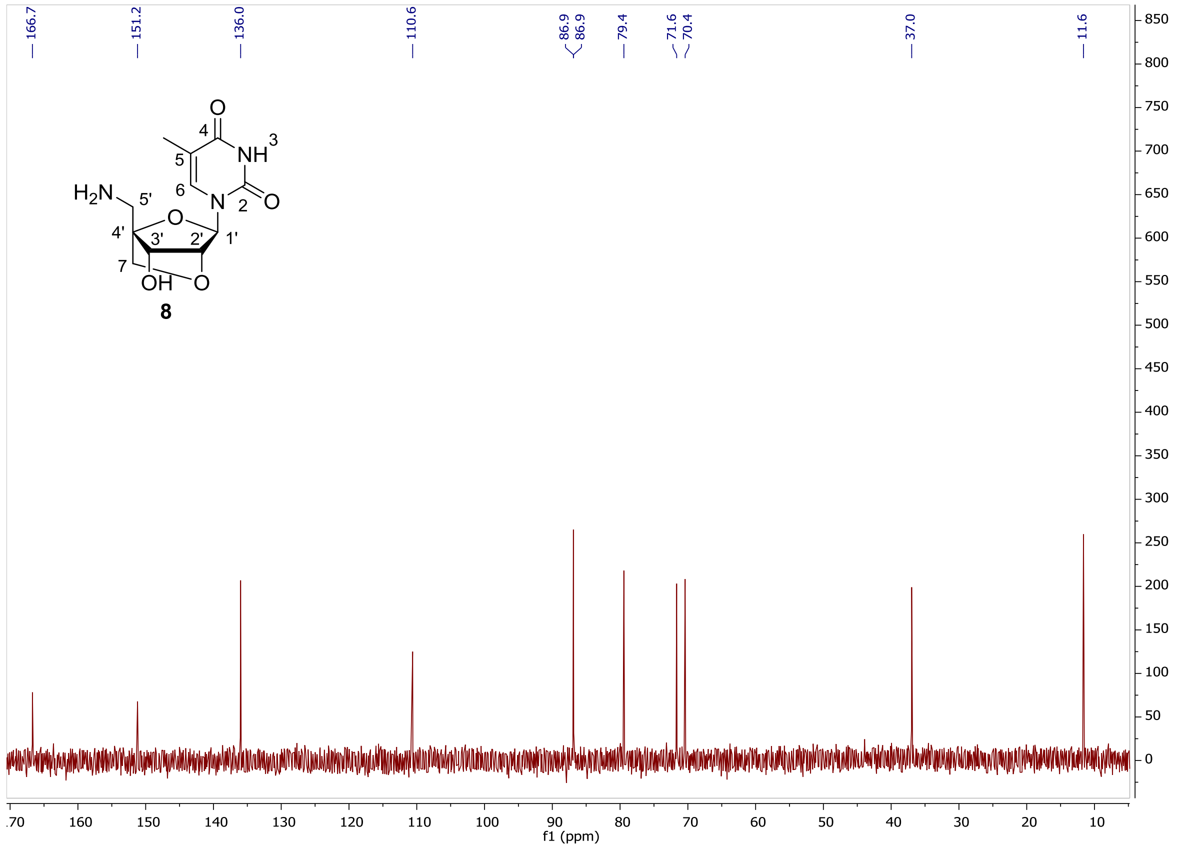
166.7
151.2
136.0
110.6
86.9
86.9
79.4
71.6
70.4
37.0
11.6
f1 (ppm)
8

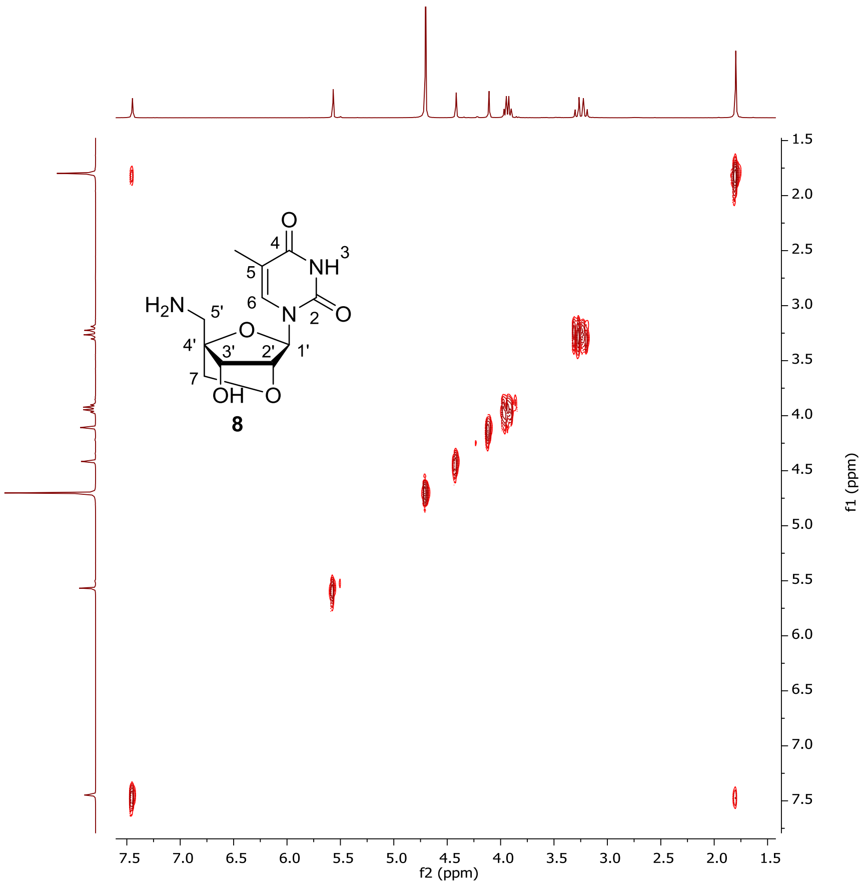
H2N
5'
4'
3'
2'
1'
7
OH
O
N
NH
6
5
4
3
2
8
f2 (ppm)
f1 (ppm)

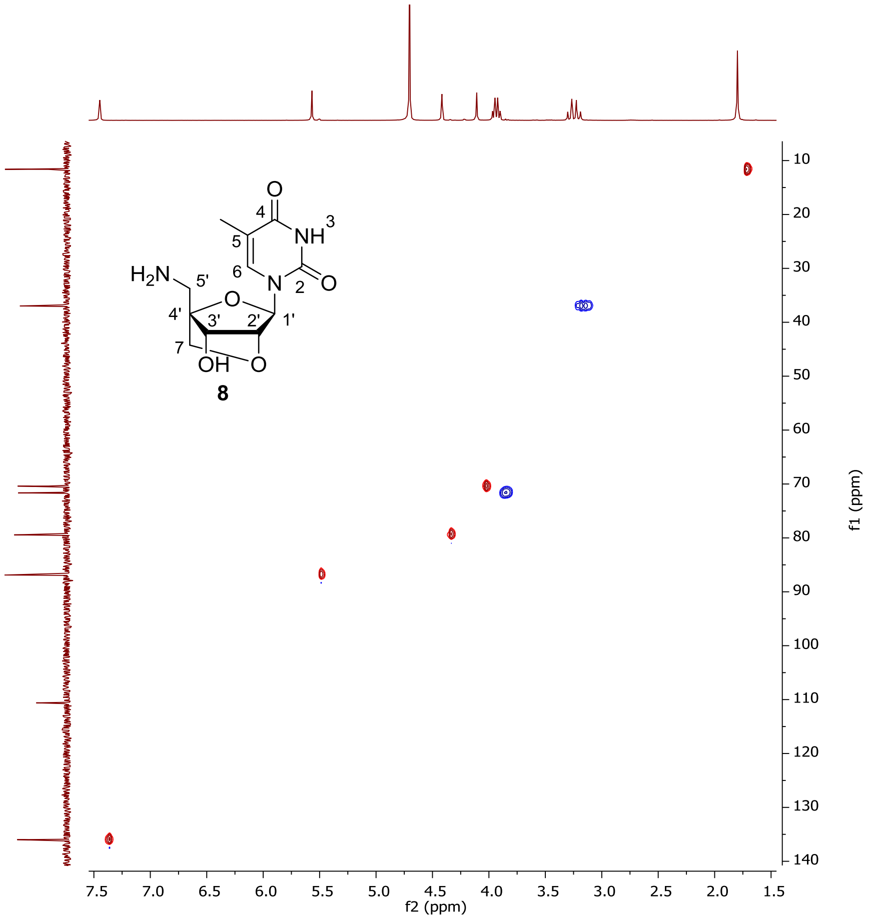
H2N
5'
4'
3'
2'
1'
7
OH
O
N
NH
6
5
4
3
2
8
f2 (ppm)
f1 (ppm)

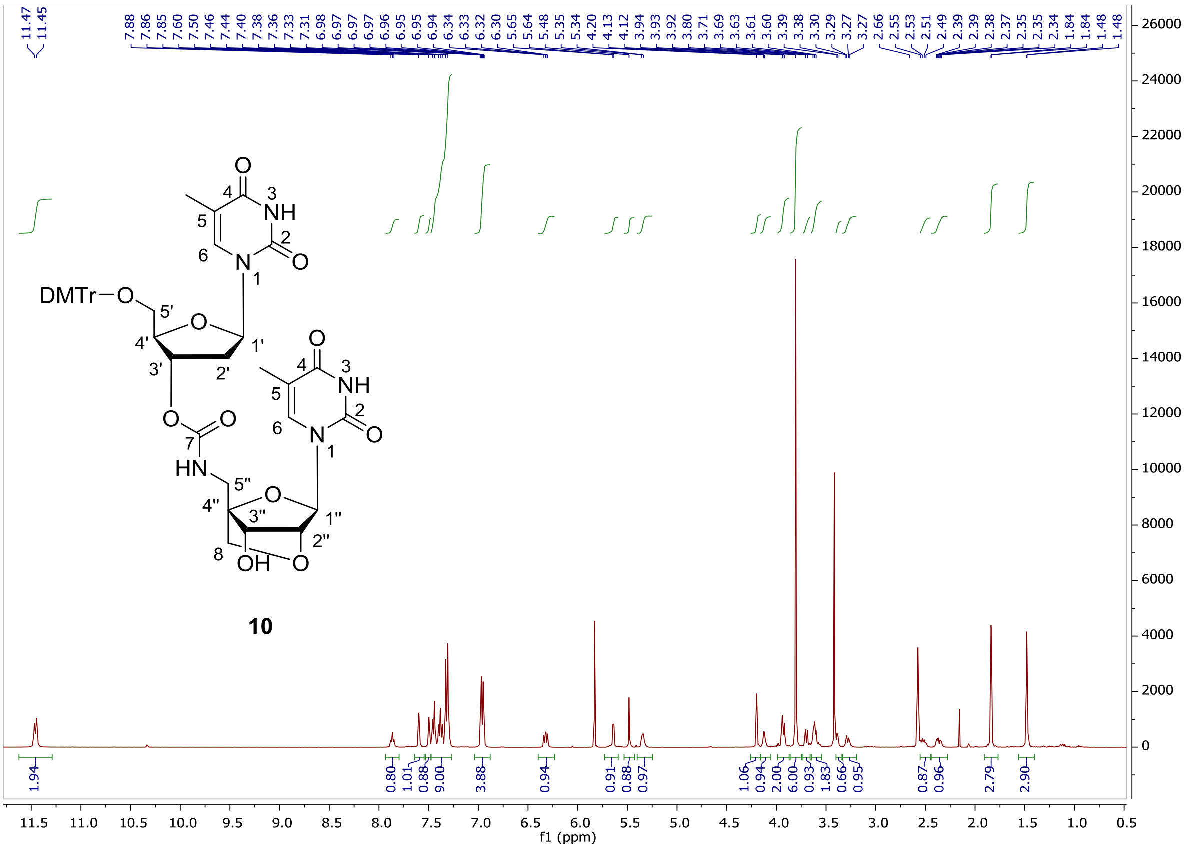
11.47
11.45
7.88
7.86
7.85
7.60
7.50
7.46
7.44
7.40
7.38
7.36
7.33
7.31
6.98
6.97
6.97
6.97
6.96
6.95
6.95
6.94
6.34
6.33
6.32
6.30
5.65
5.64
5.48
5.35
5.34
4.20
4.13
4.12
3.94
3.93
3.92
3.80
3.71
3.69
3.63
3.61
3.60
3.39
3.38
3.30
3.29
3.27
3.27
2.66
2.55
2.53
2.51
2.49
2.39
2.39
2.38
2.37
2.35
2.34
1.84
1.84
1.48
1.48
DMTr
O
NH
N
HN
OH
10
1.94
0.80
1.01
0.88
9.00
3.88
0.94
0.91
0.88
0.97
1.06
0.94
2.00
6.00
0.93
1.83
0.66
0.95
0.87
0.96
2.79
2.90
f1 (ppm)

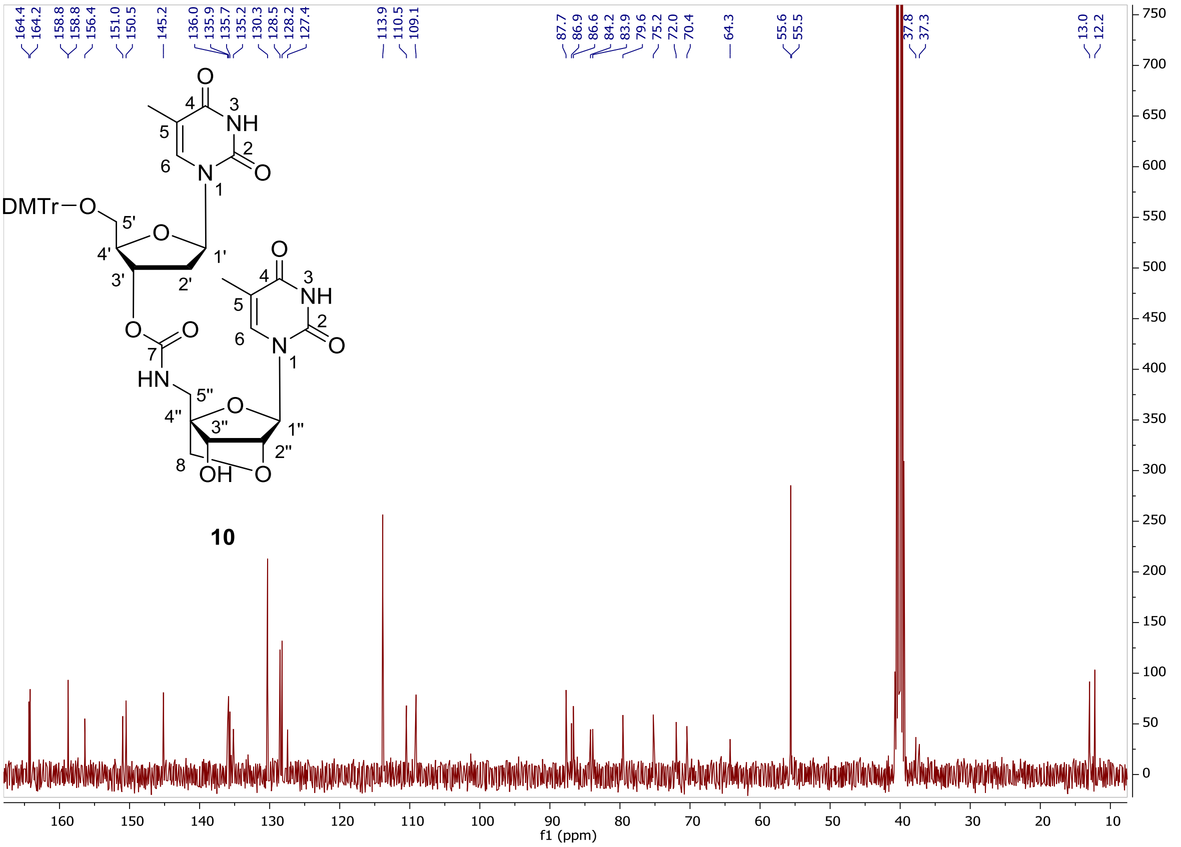
164.4
164.2
158.8
158.8
156.4
151.0
150.5
145.2
136.0
135.9
135.7
135.2
130.3
128.5
128.2
127.4
113.9
110.5
109.1
87.7
86.9
86.6
84.2
83.9
79.6
75.2
72.0
70.4
64.3
55.6
55.5
37.8
37.3
13.0
12.2
DMTr
O
NH
N
HN
OH
10
f1 (ppm)

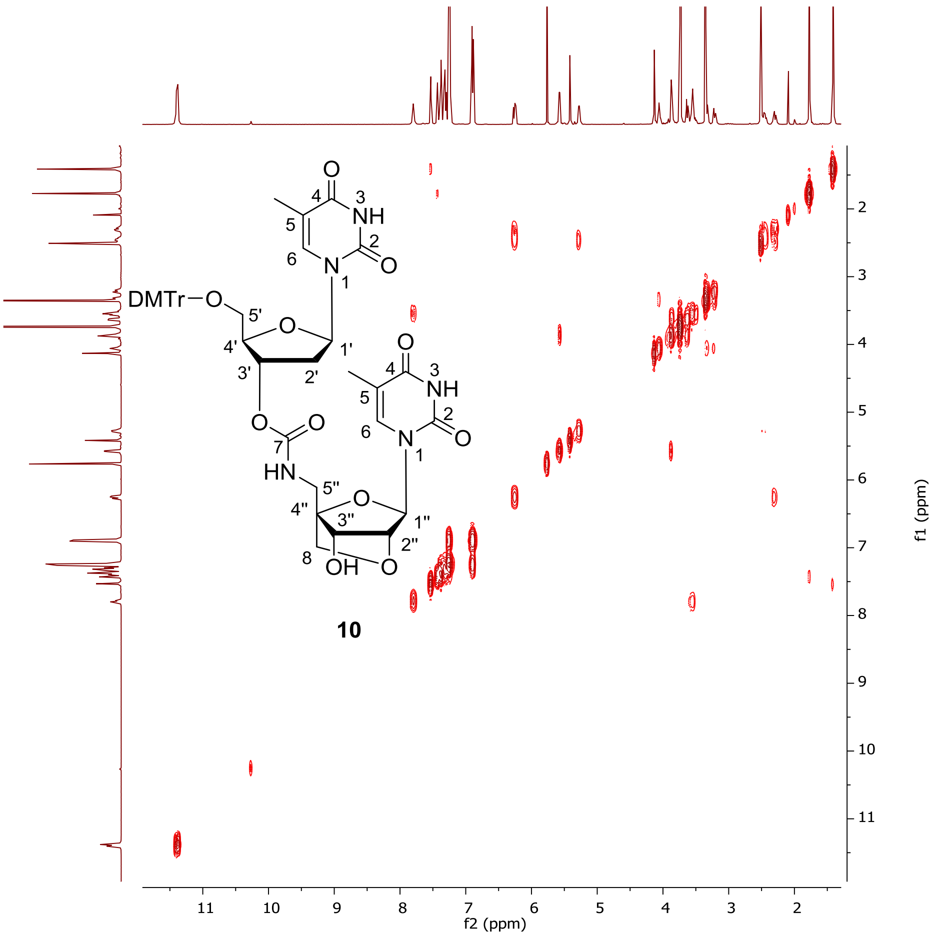
DMTr—O
10
f2 (ppm)
f1 (ppm)

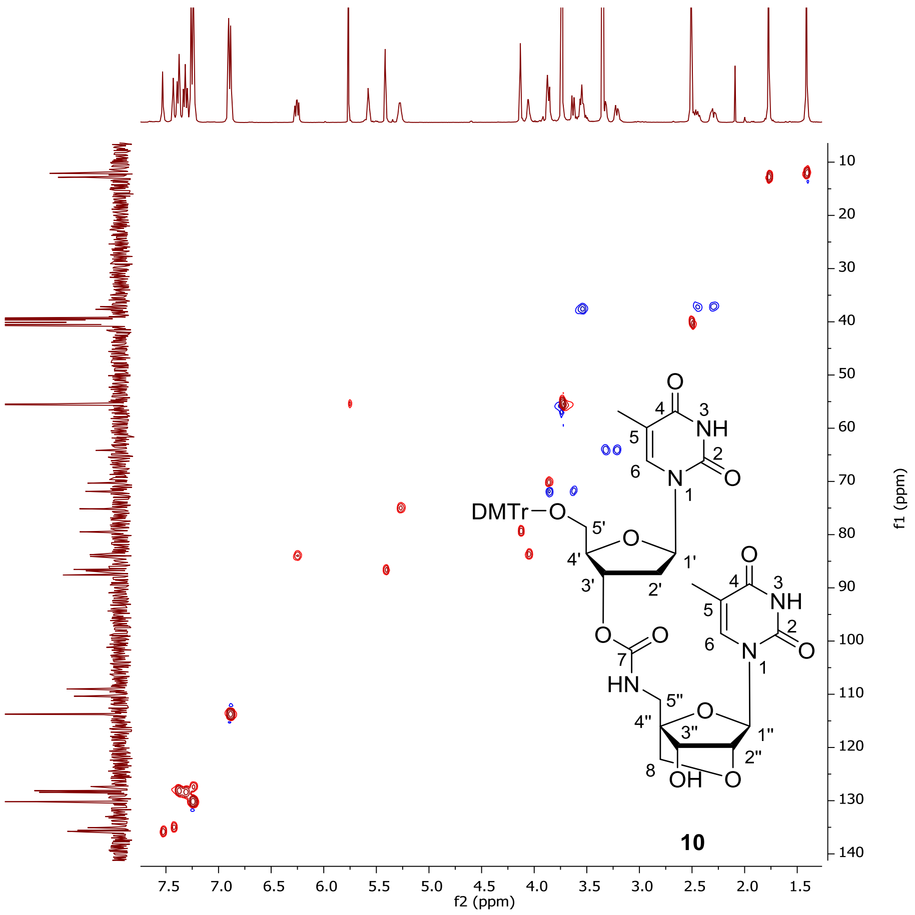
DMTr—O
10
f2 (ppm)
f1 (ppm)

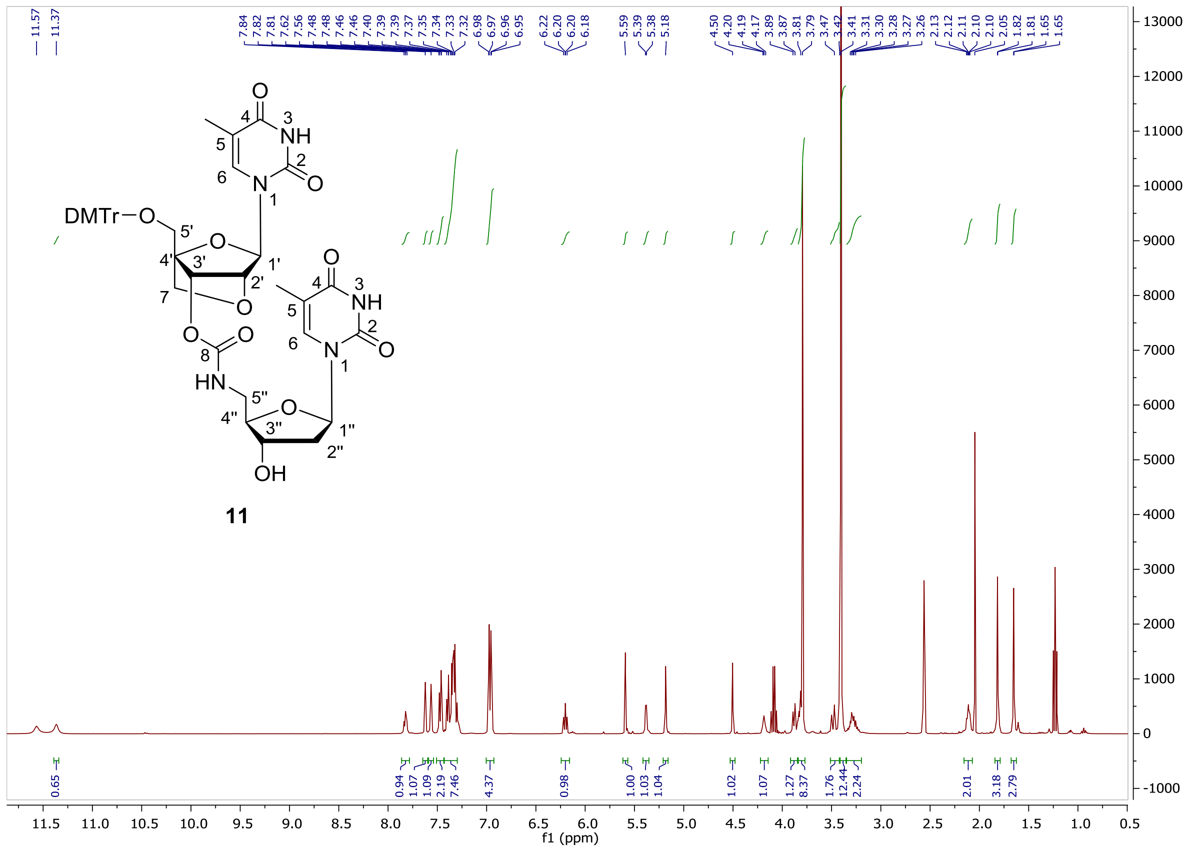

DMTr−O
11
f1 (ppm)

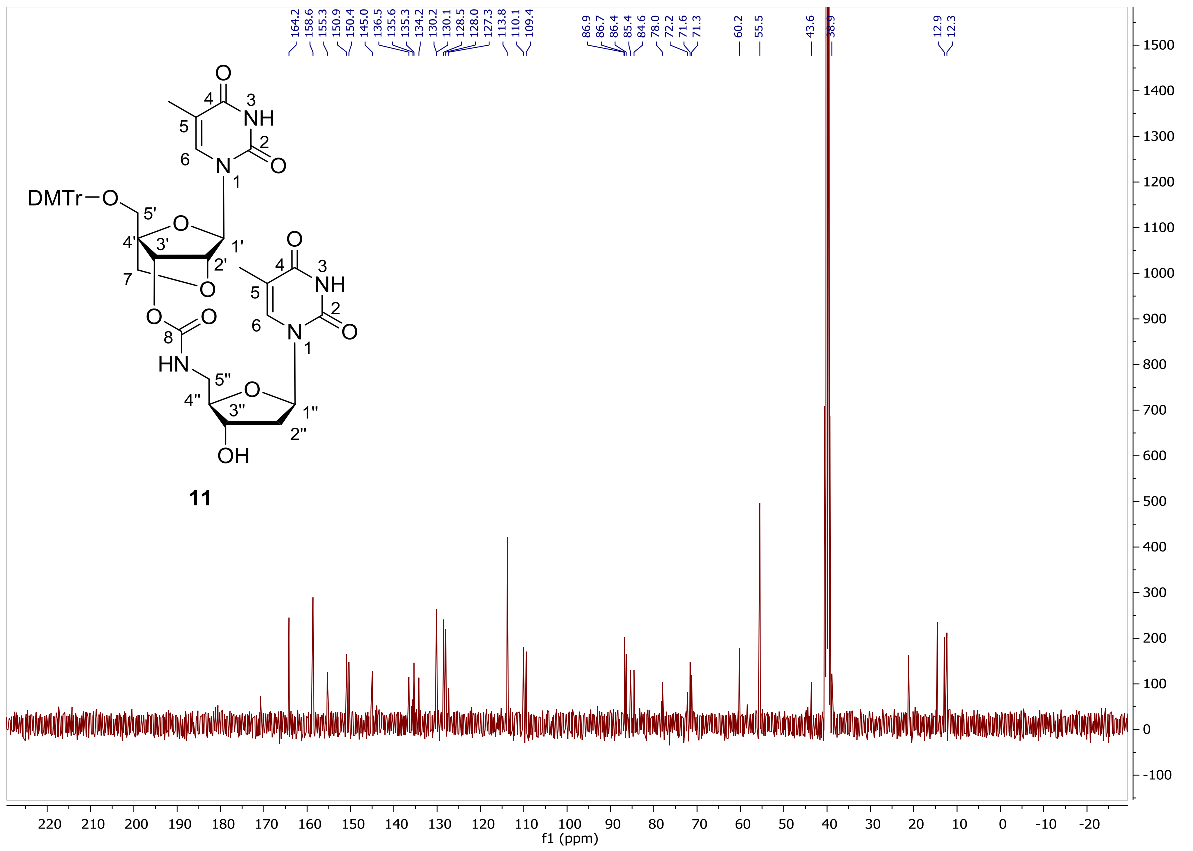

DMTr−O
11
f1 (ppm)

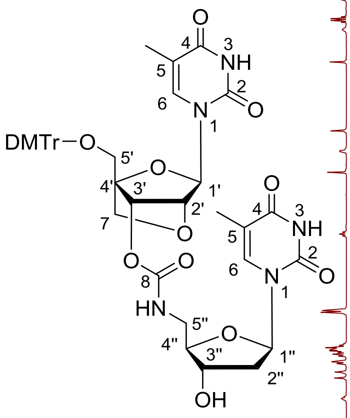

11

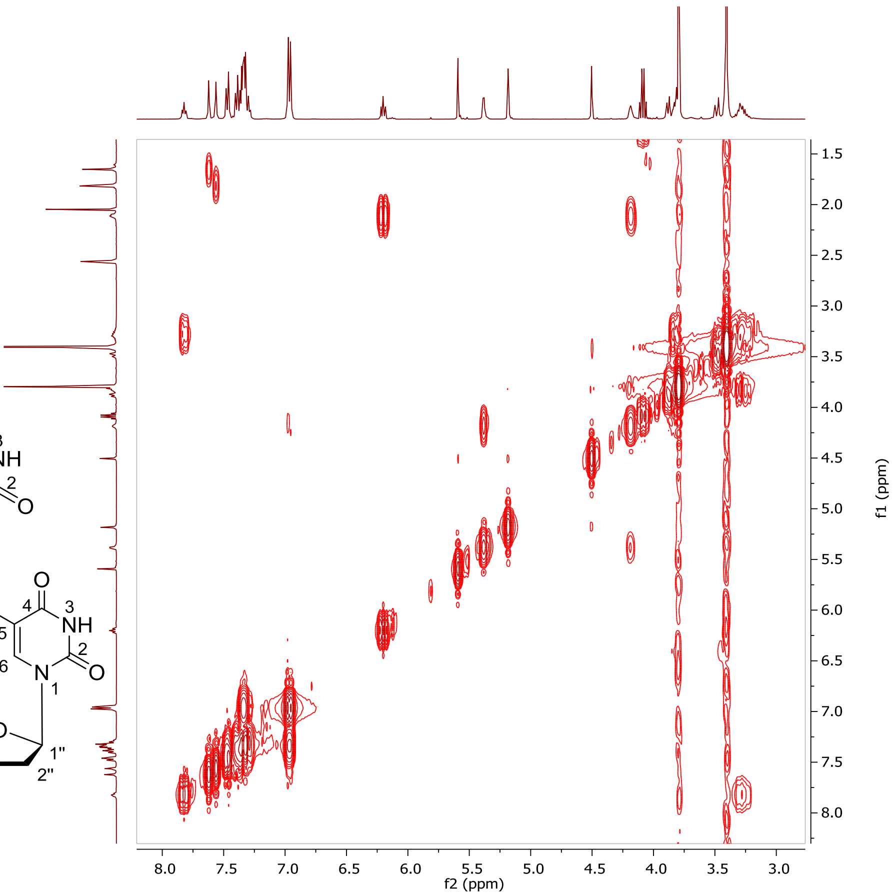

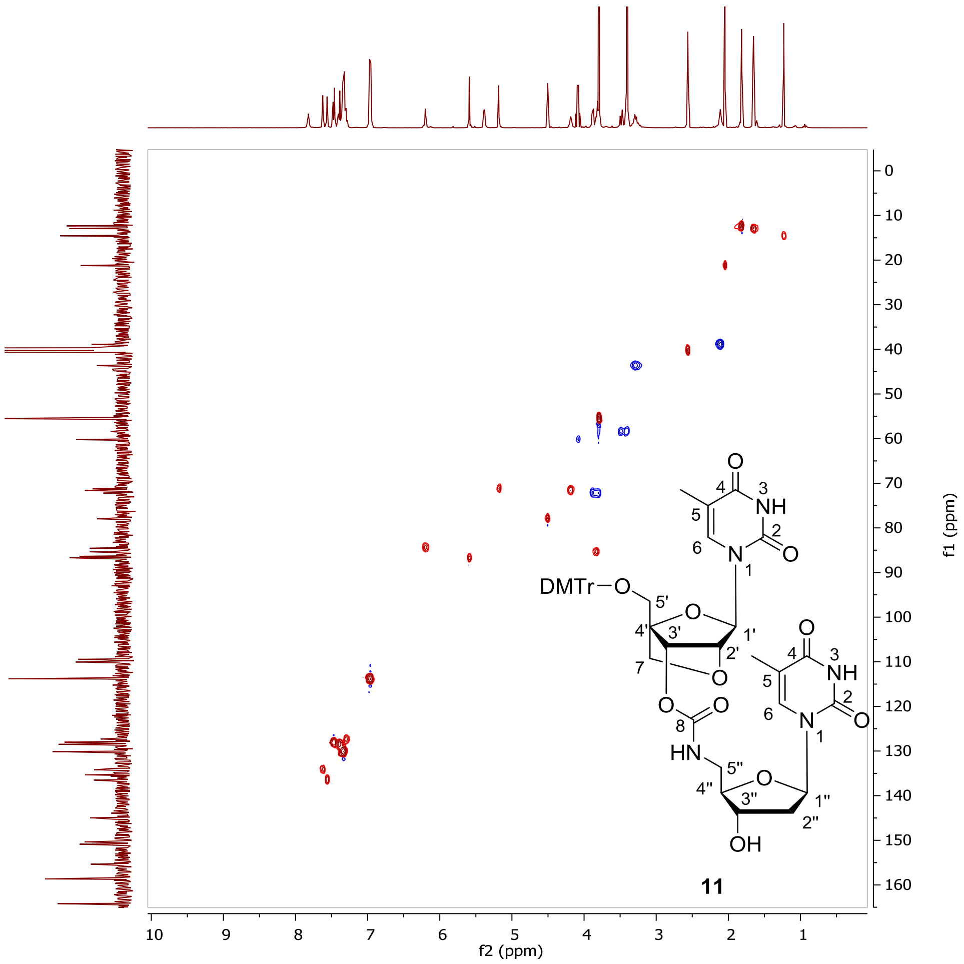
f2 (ppm)
f1 (ppm)
DMTr—O
11

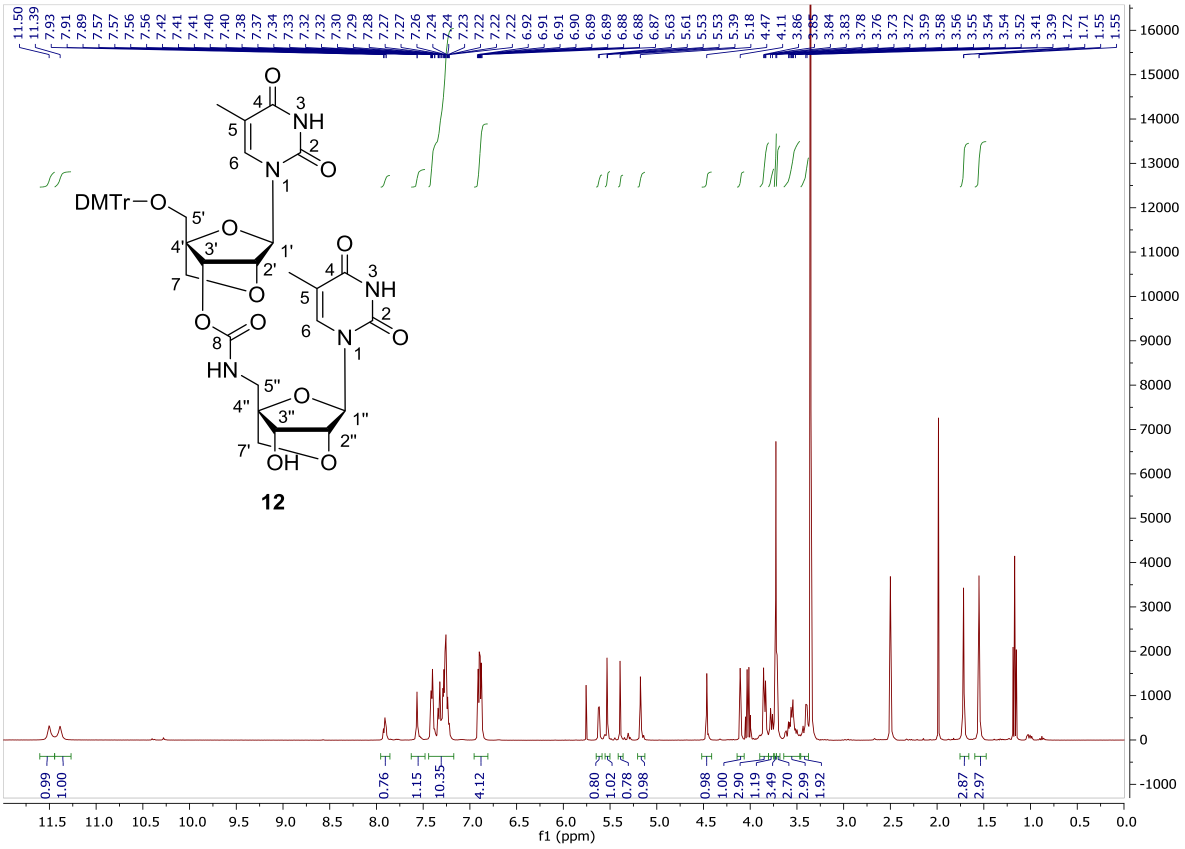
f1 (ppm)
DMTr—O
12

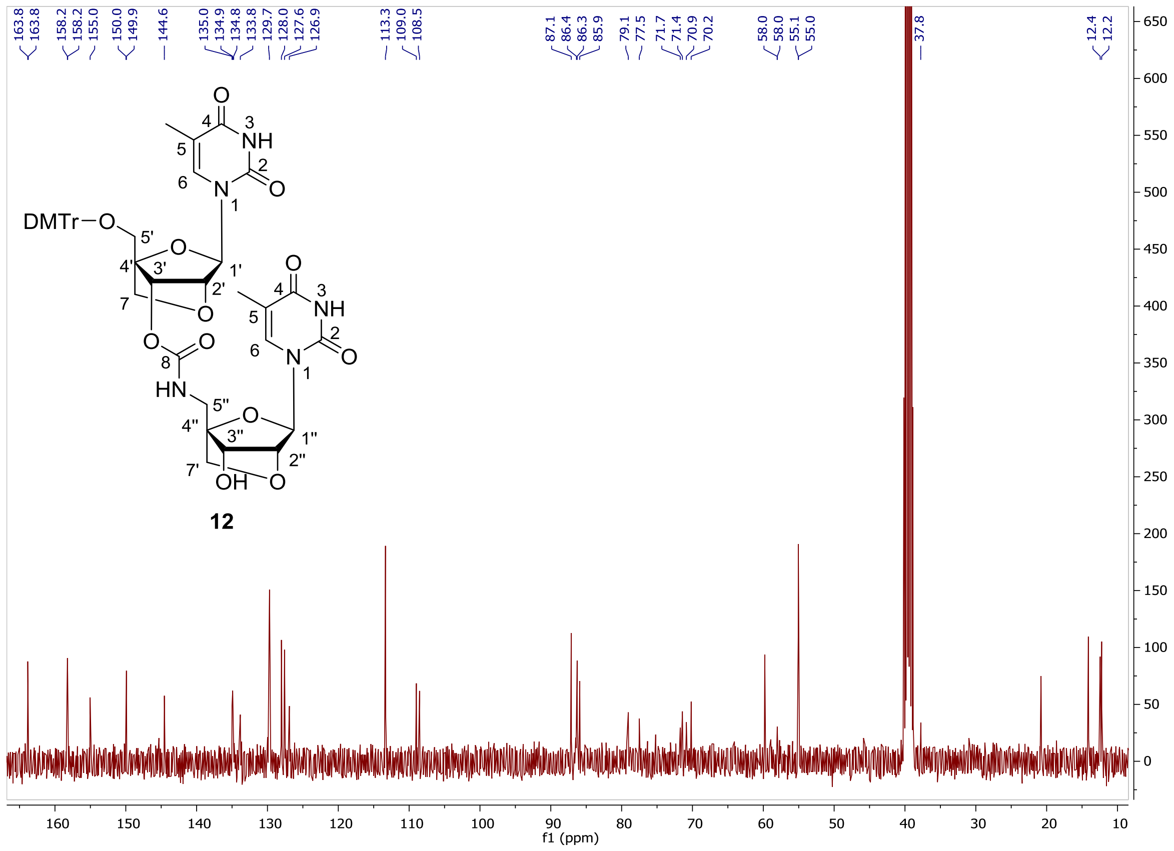
163.8
163.8
158.2
158.2
155.0
150.0
149.9
144.6
135.0
134.9
134.8
133.8
129.7
128.0
127.6
126.9
113.3
109.0
108.5
87.1
86.4
86.3
85.9
79.1
77.5
71.7
71.4
70.9
70.2
58.0
58.0
55.1
55.0
37.8
12.4
12.2
DMTr
12
f1 (ppm)

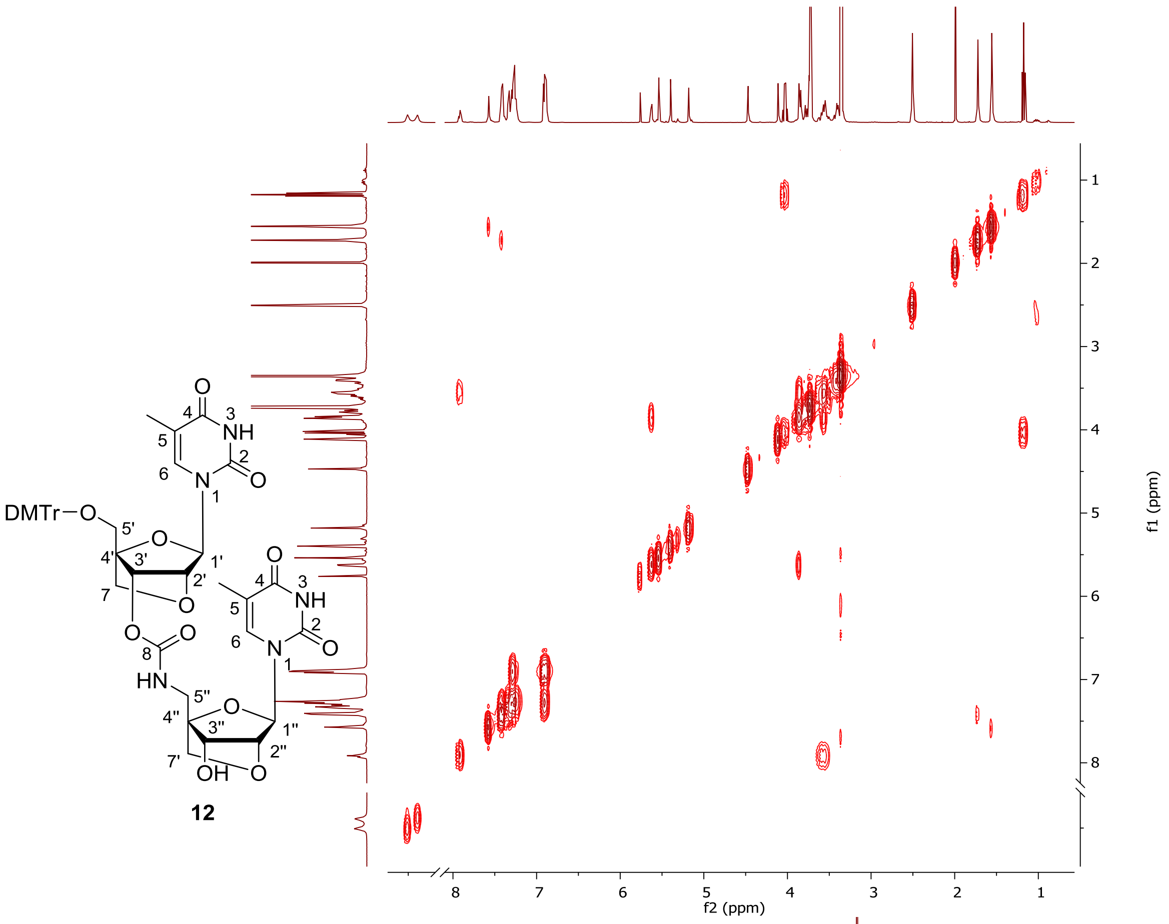
DMTr–O
12
f2 (ppm)
f1 (ppm)

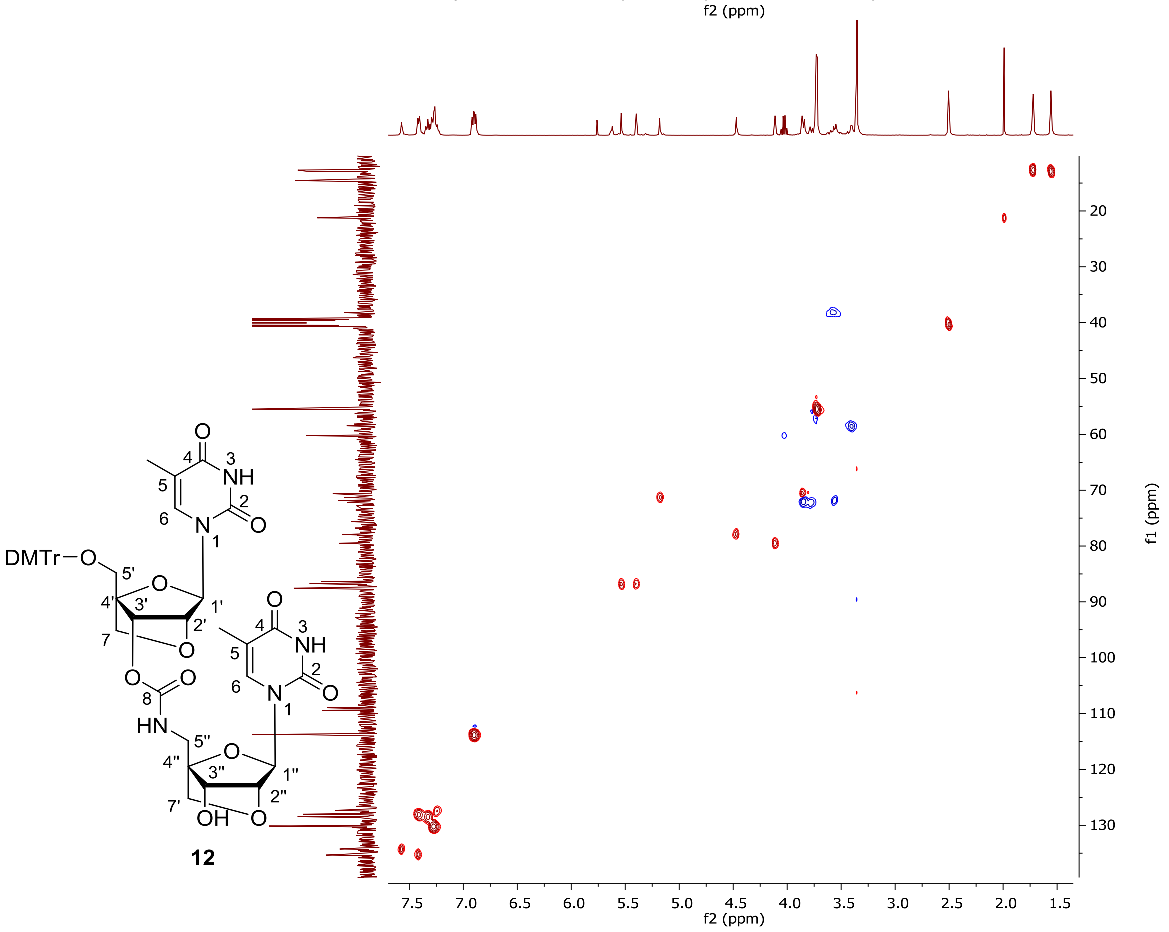
DMTr–O
12
f2 (ppm)
f1 (ppm)

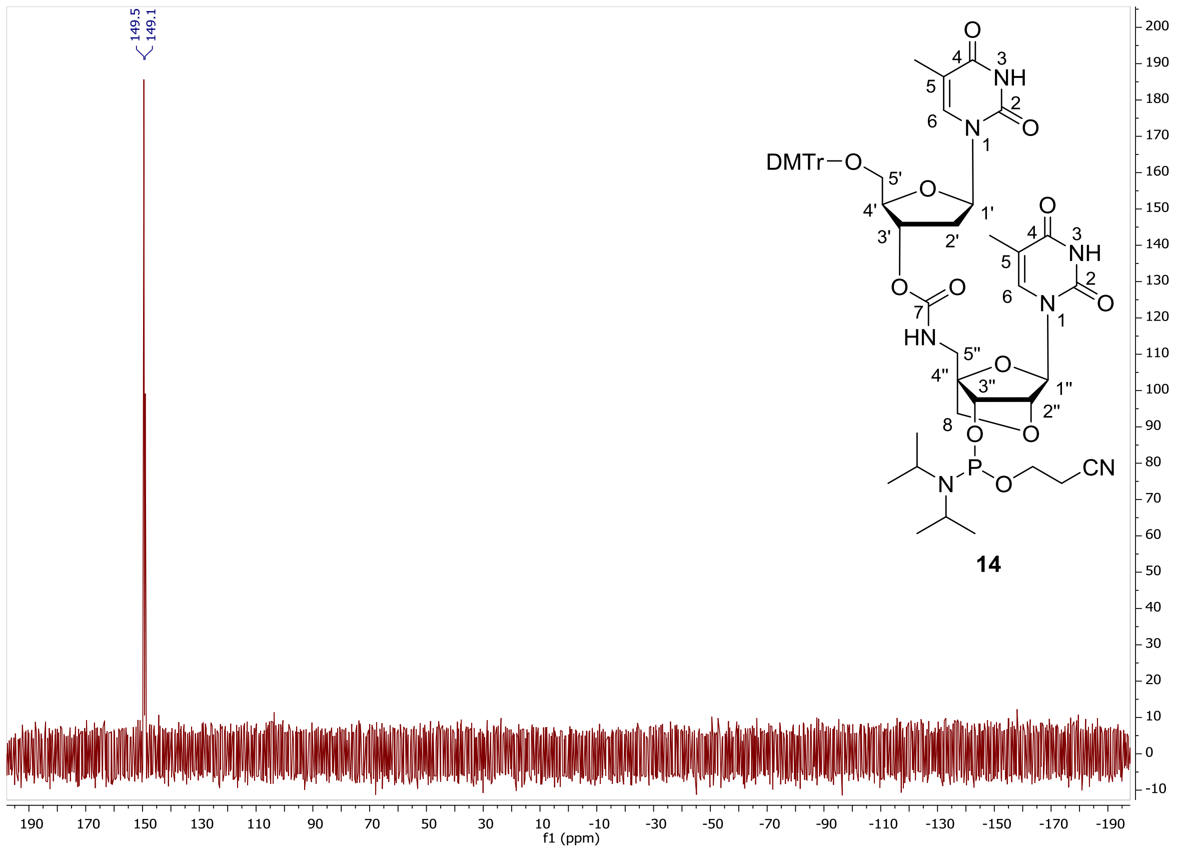
149.5
149.1
DMTr
14
f1 (ppm)

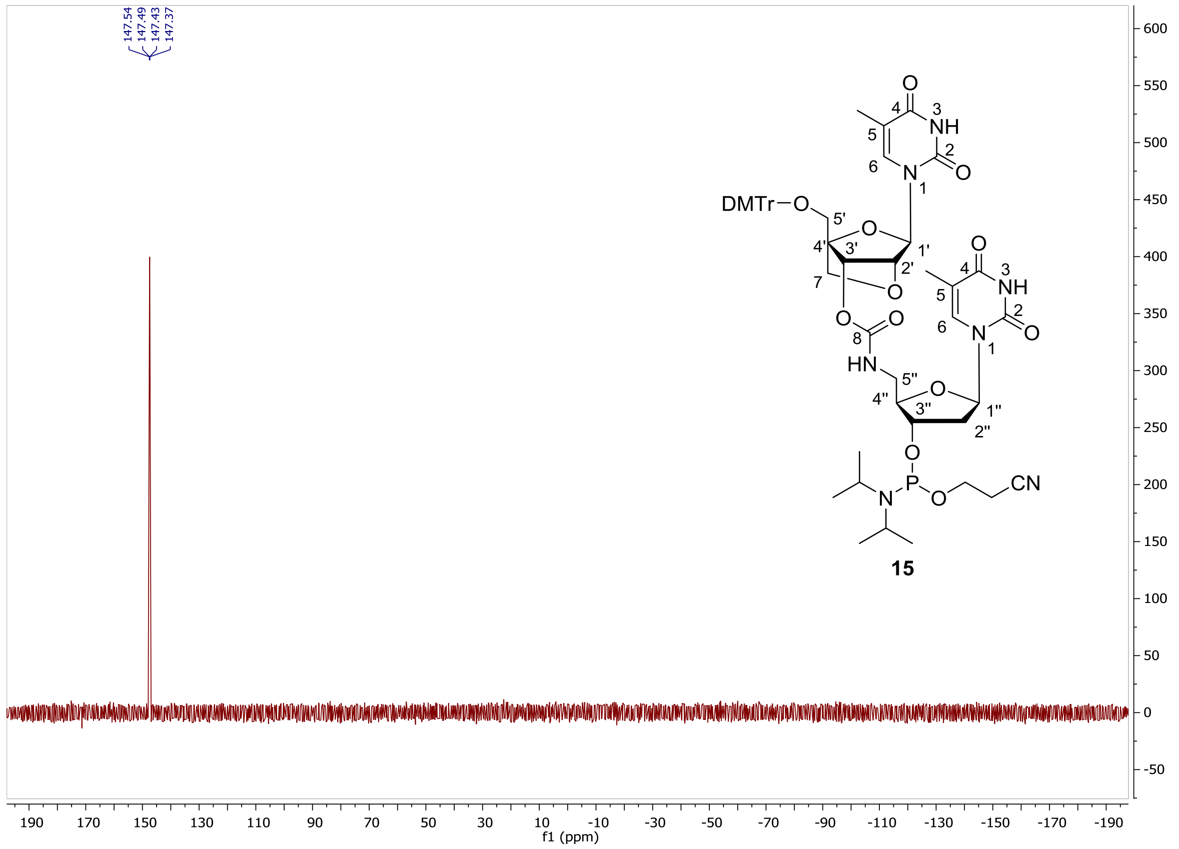
147.54
147.49
147.43
147.37
DMTr
15
f1 (ppm)

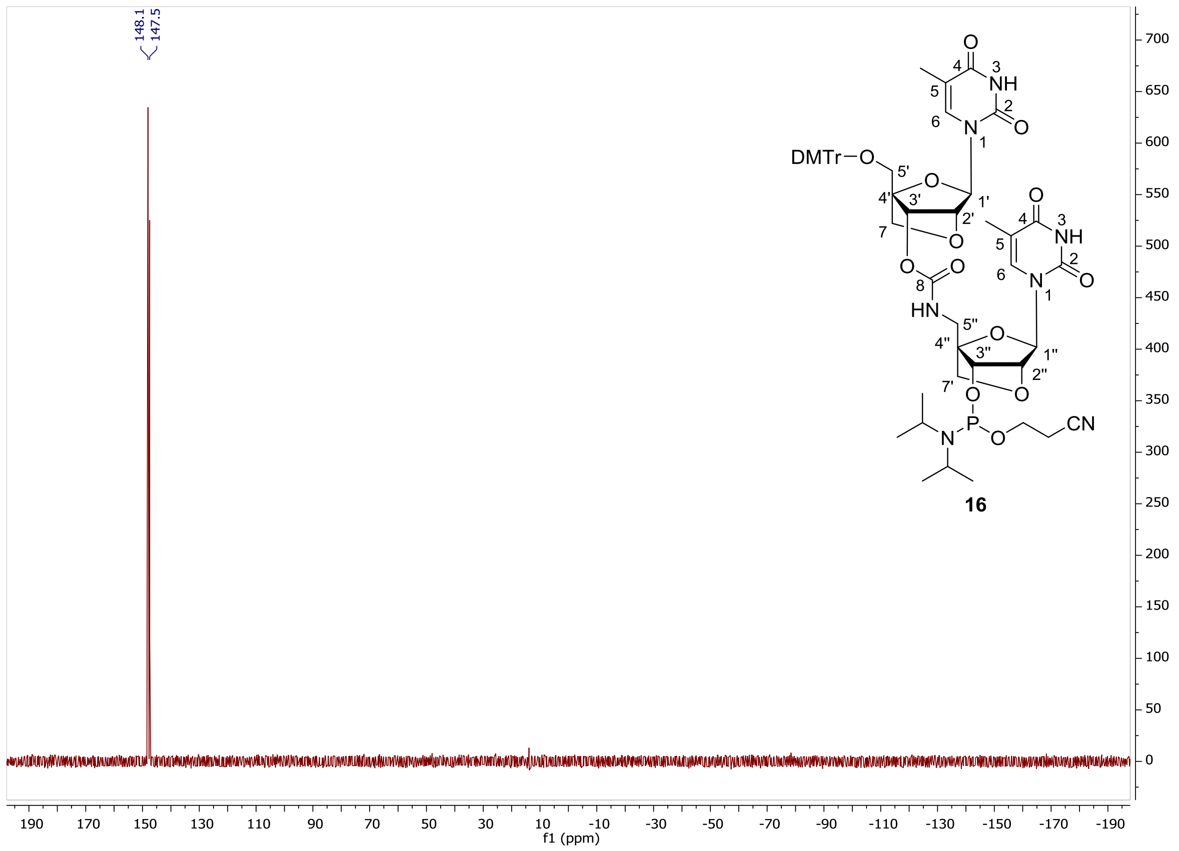
148.1
147.5
DMTr
16
f1 (ppm)

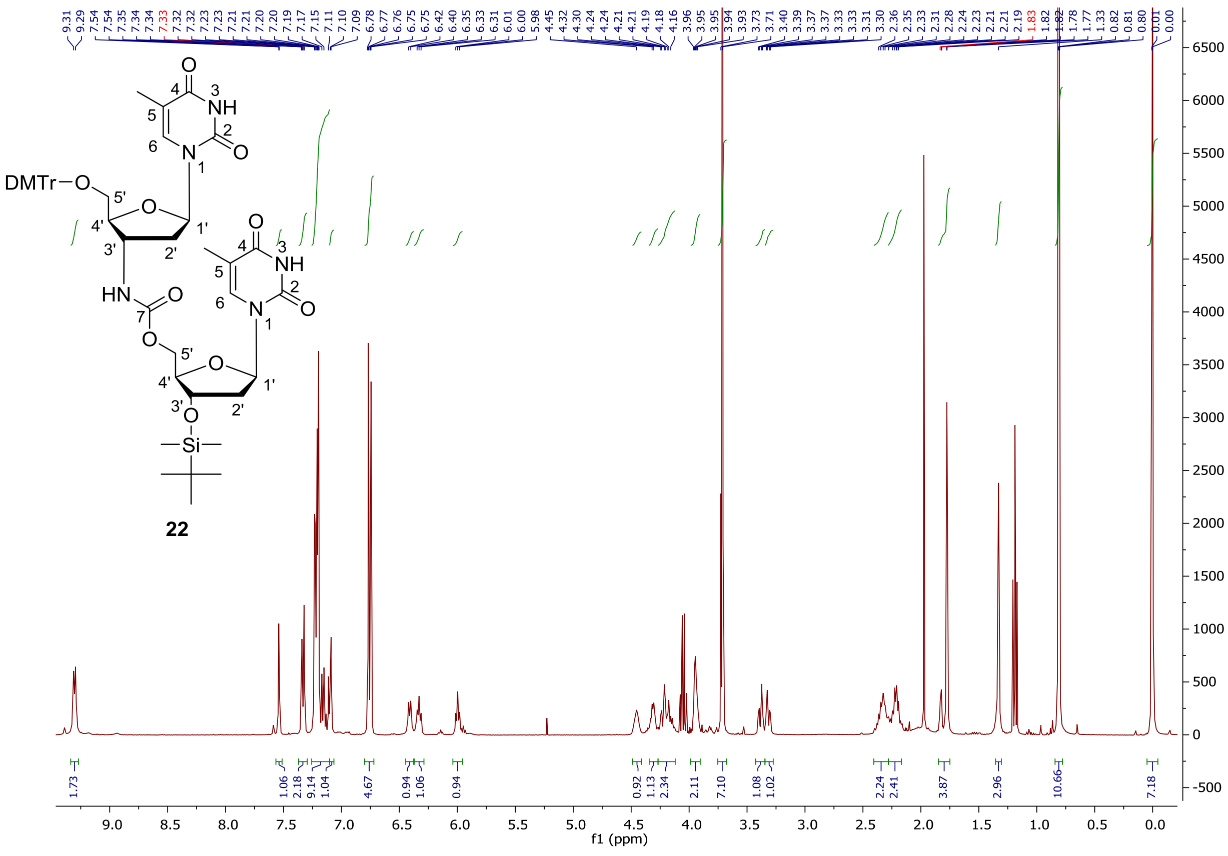
DMTr
22
f1 (ppm)

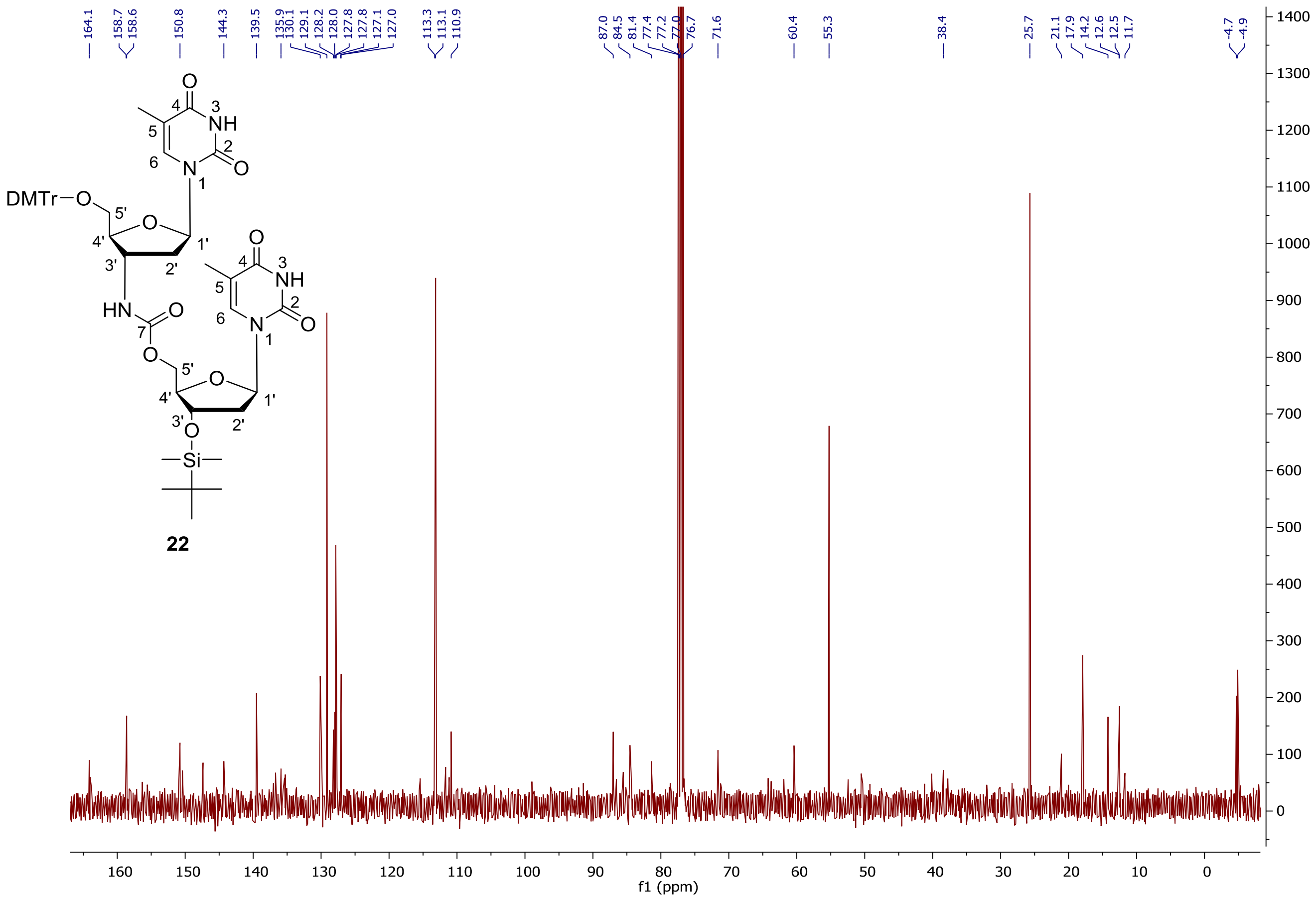

164.1
158.7
158.6
150.8
144.3
139.5
135.9
130.1
129.1
128.2
128.0
127.8
127.8
127.1
127.0
113.3
113.1
110.9
87.0
84.5
81.4
77.4
77.2
77.0
76.7
71.6
60.4
55.3
38.4
25.7
21.1
17.9
14.2
12.6
12.5
11.7
-4.7
-4.9
DMTr
22
f1 (ppm)

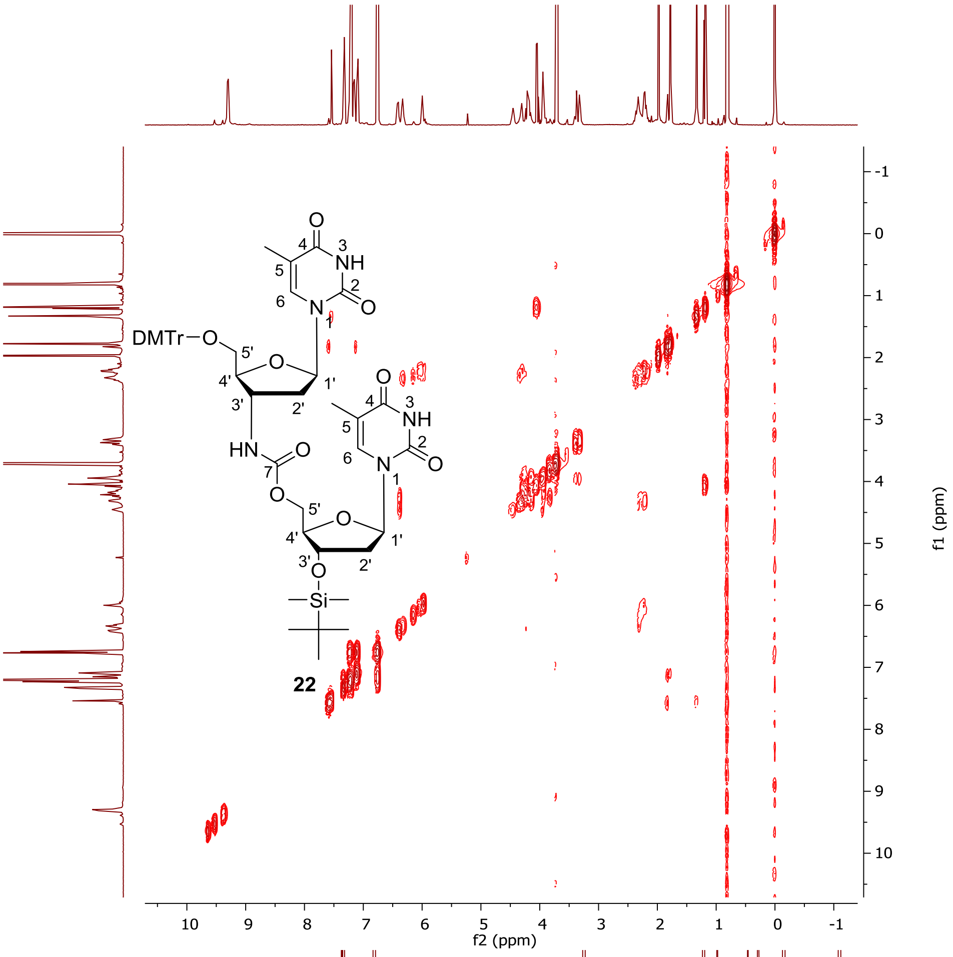
DMTr–O
22
f2 (ppm)
f1 (ppm)

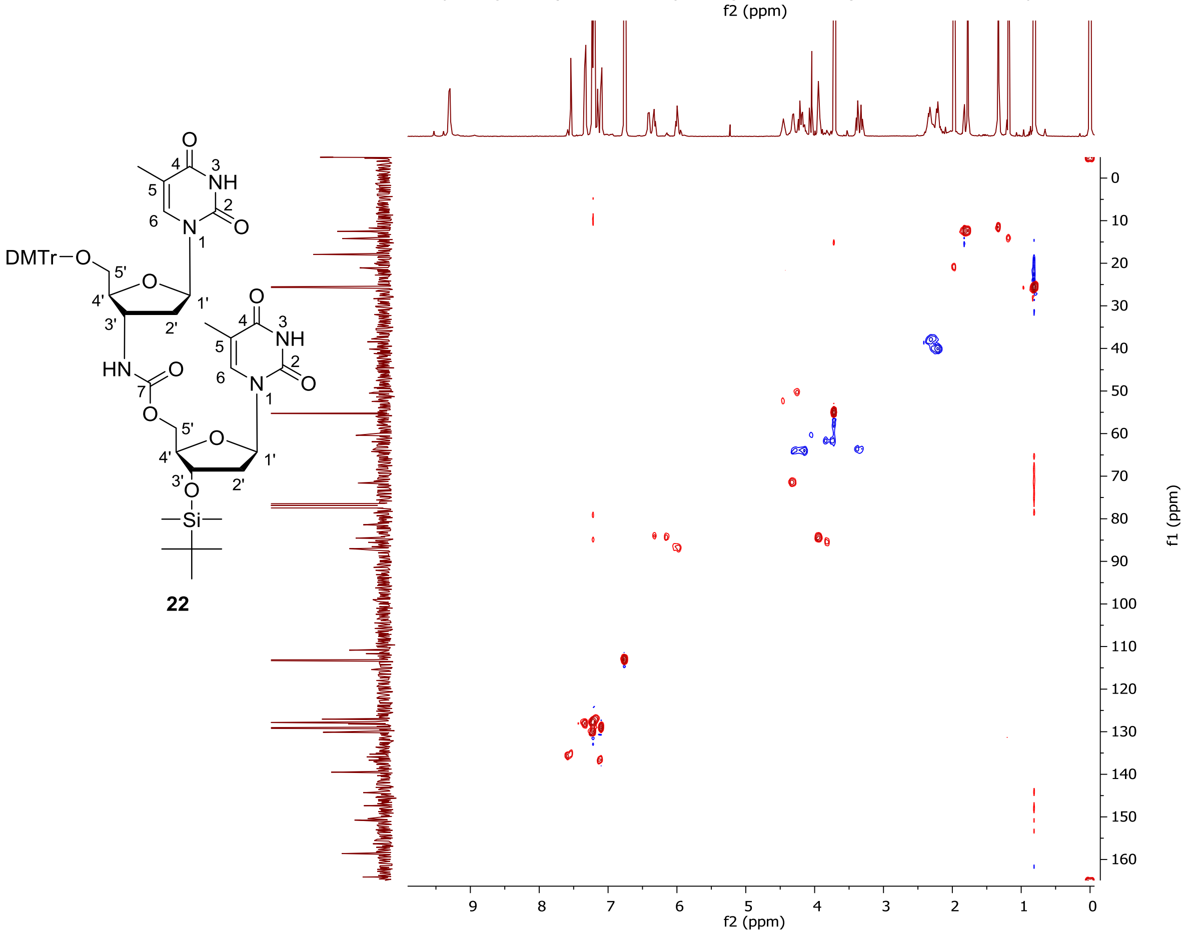
DMTr–O
22
f2 (ppm)
f1 (ppm)

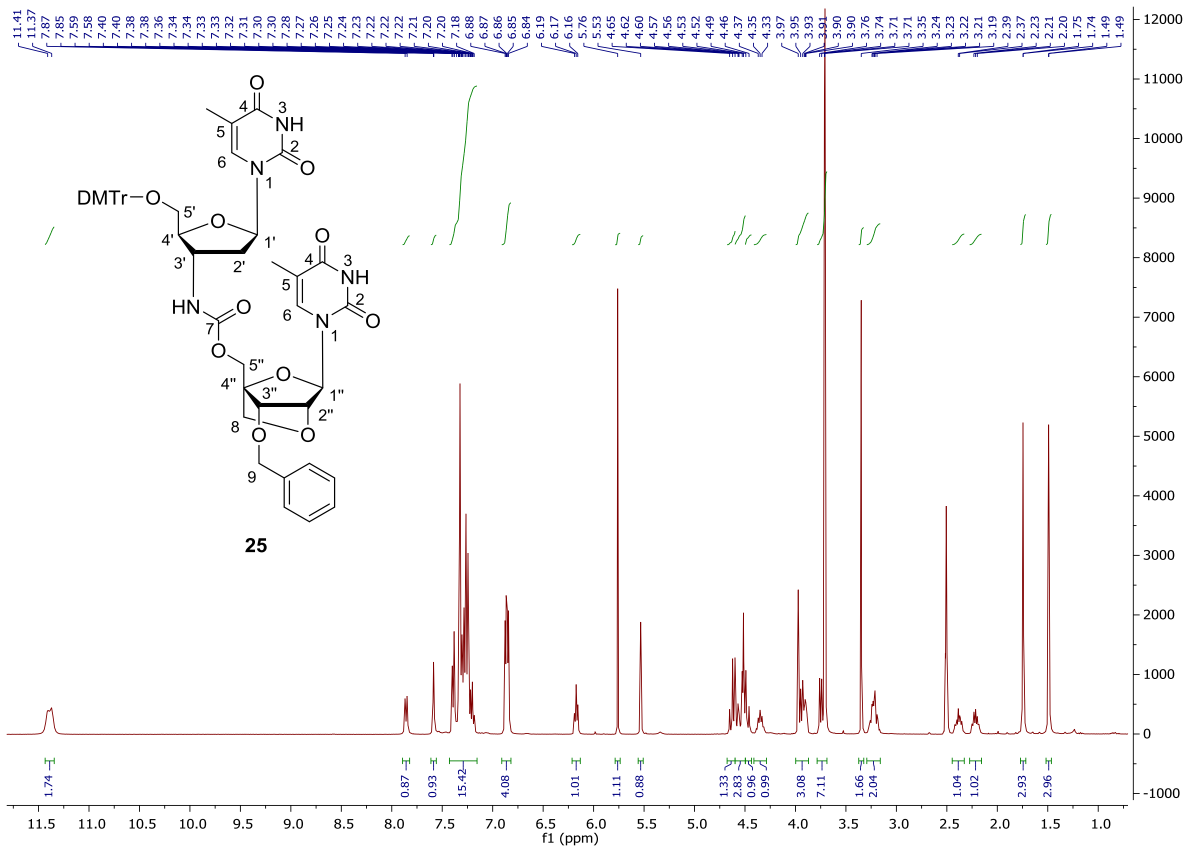

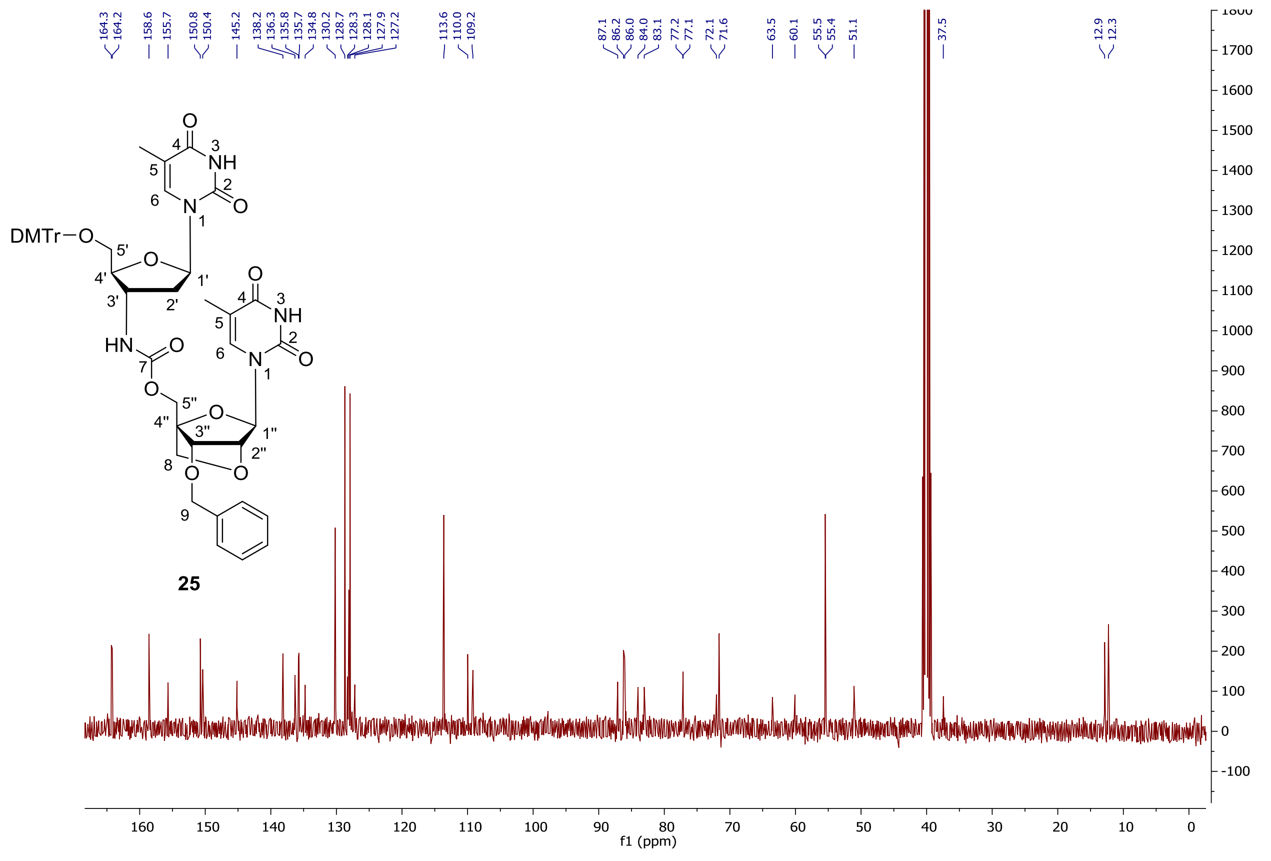

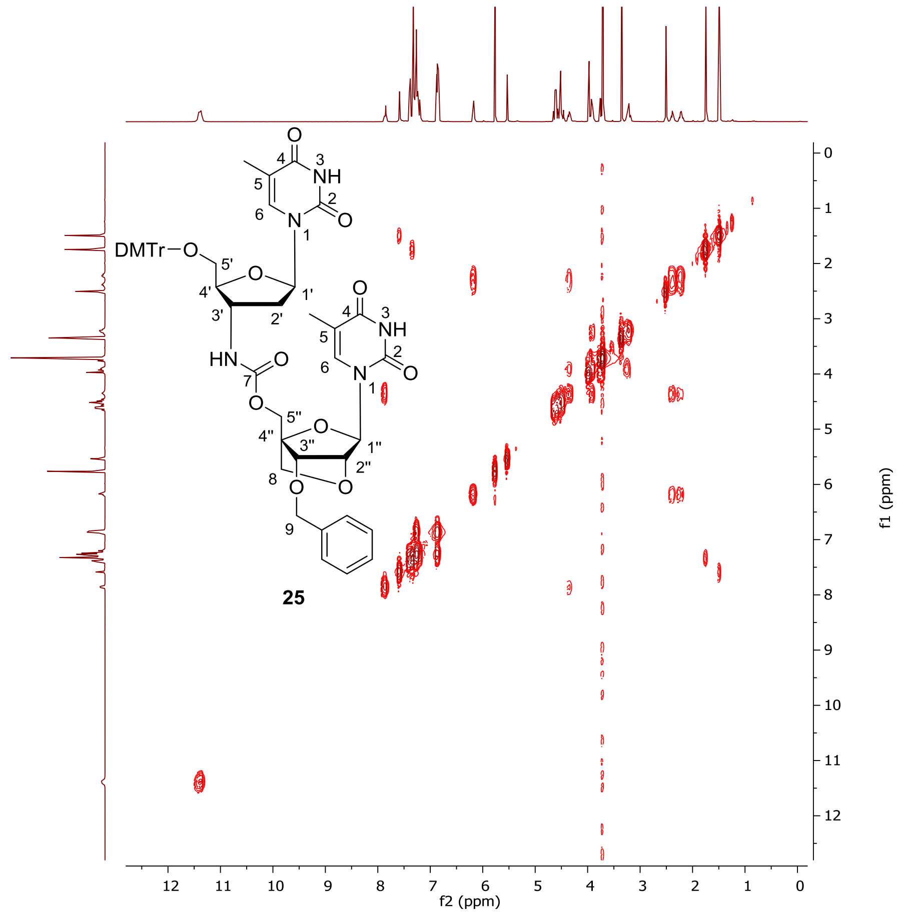
DMTr
25
f2 (ppm)
f1 (ppm)

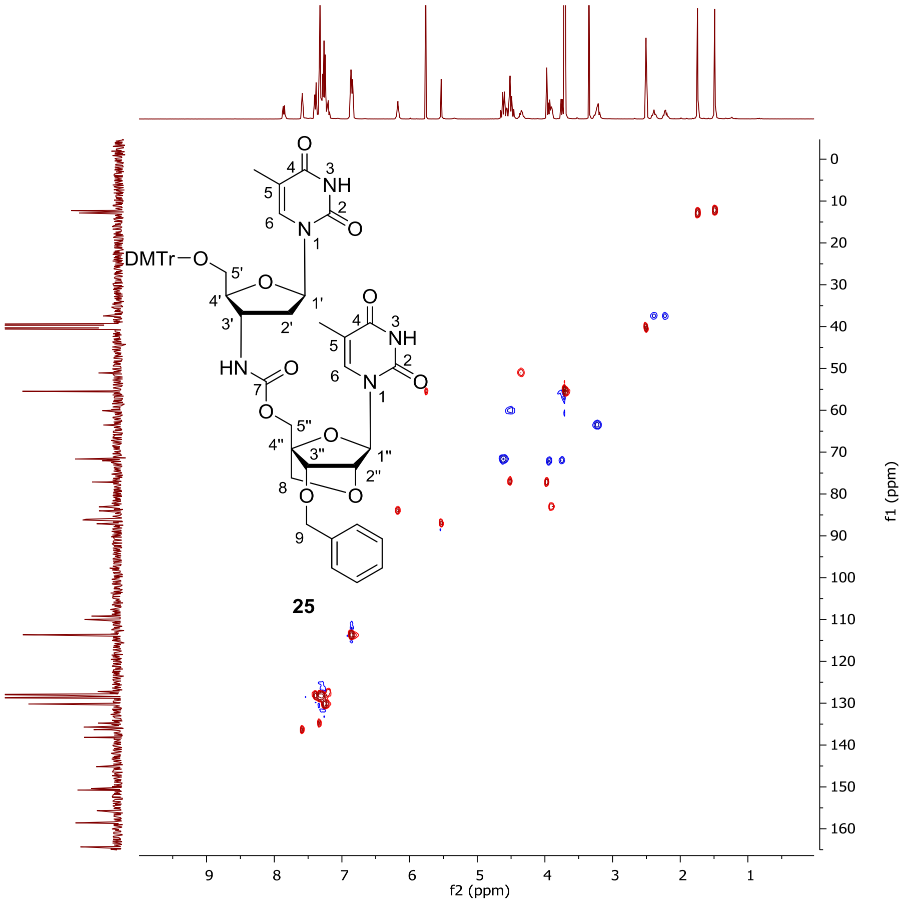
DMTr
25
f2 (ppm)
f1 (ppm)

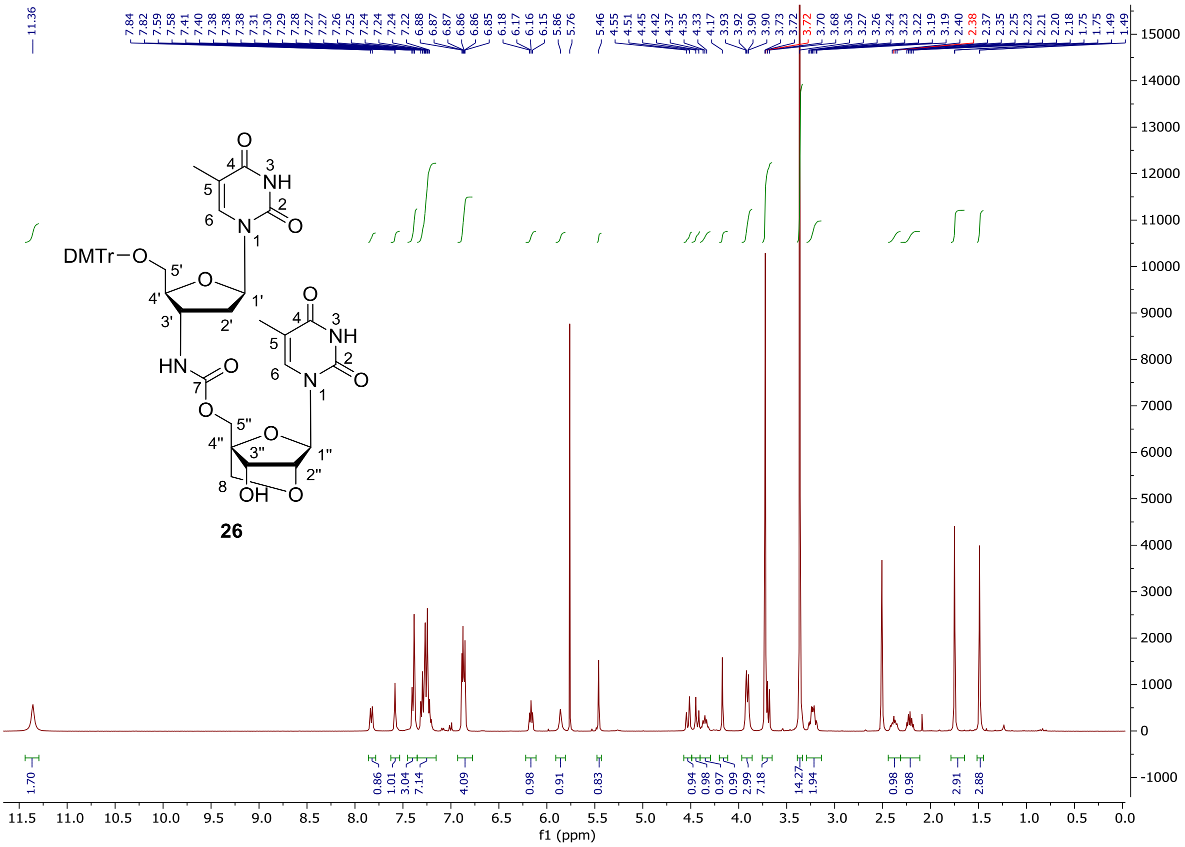

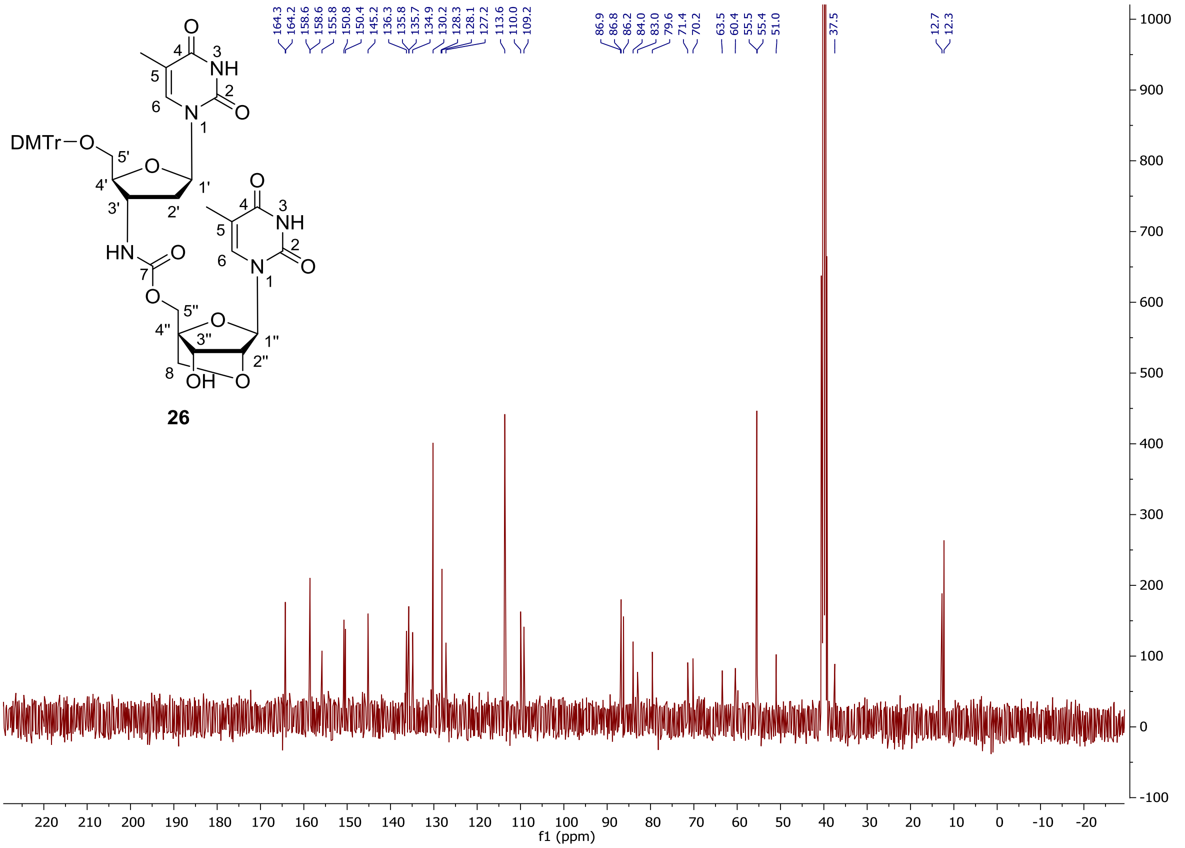

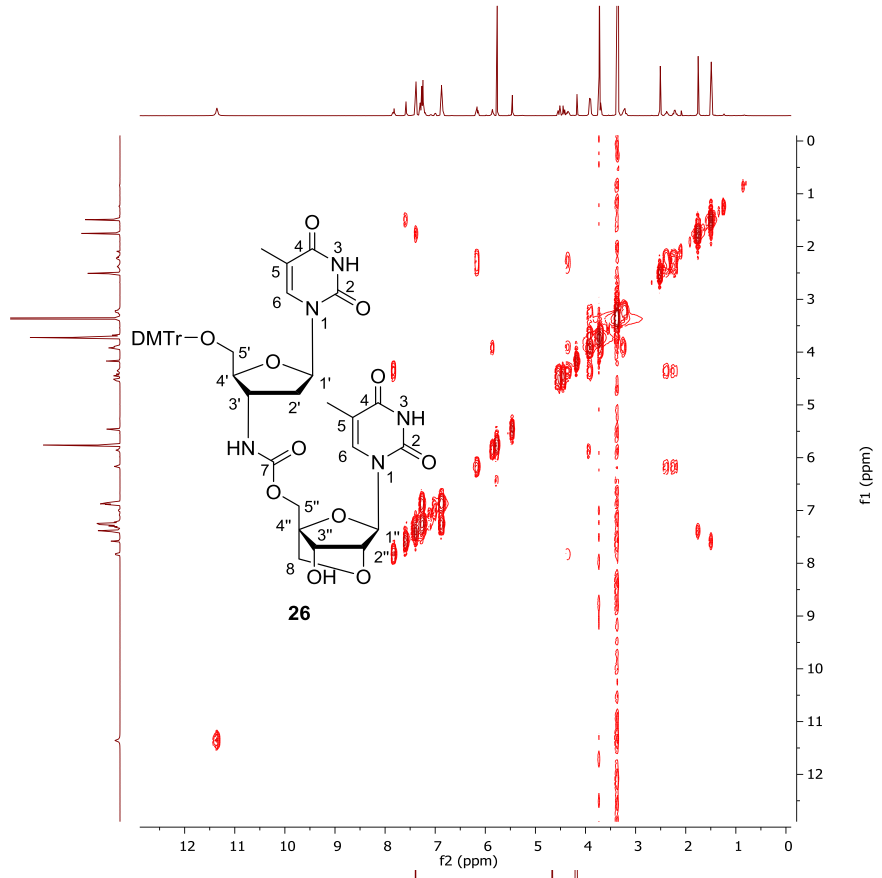
DMTr—O
HN
OH
26
f2 (ppm)
f1 (ppm)

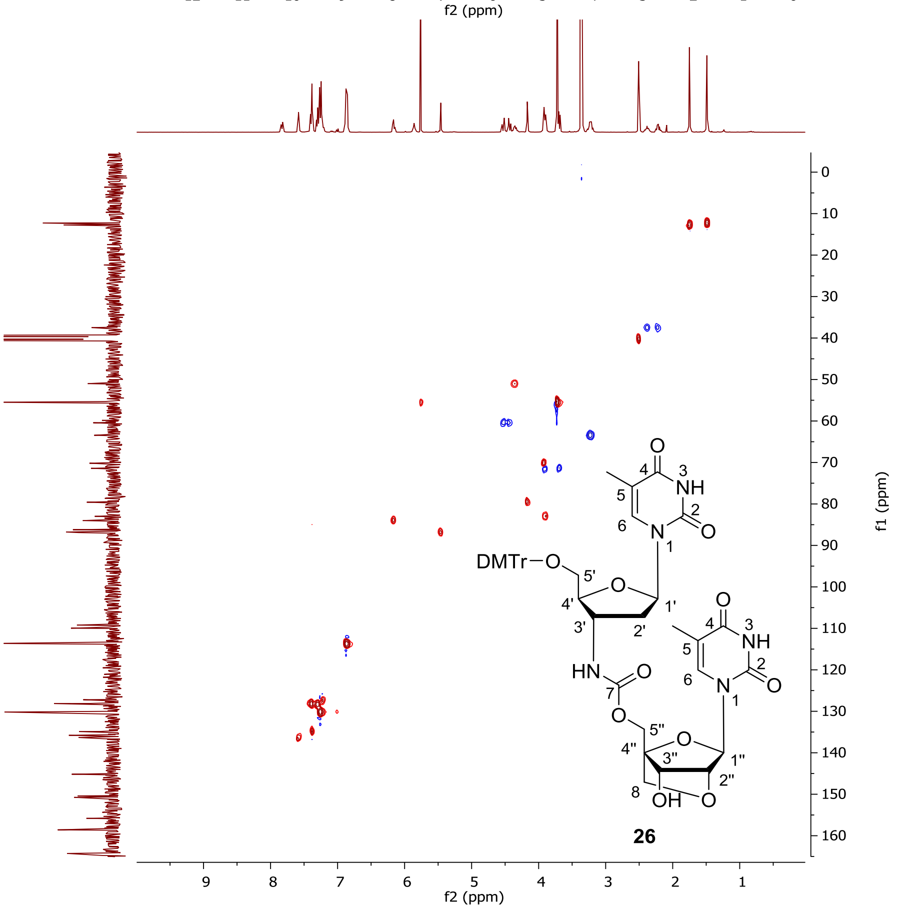
DMTr—O
HN
OH
26
f2 (ppm)
f1 (ppm)

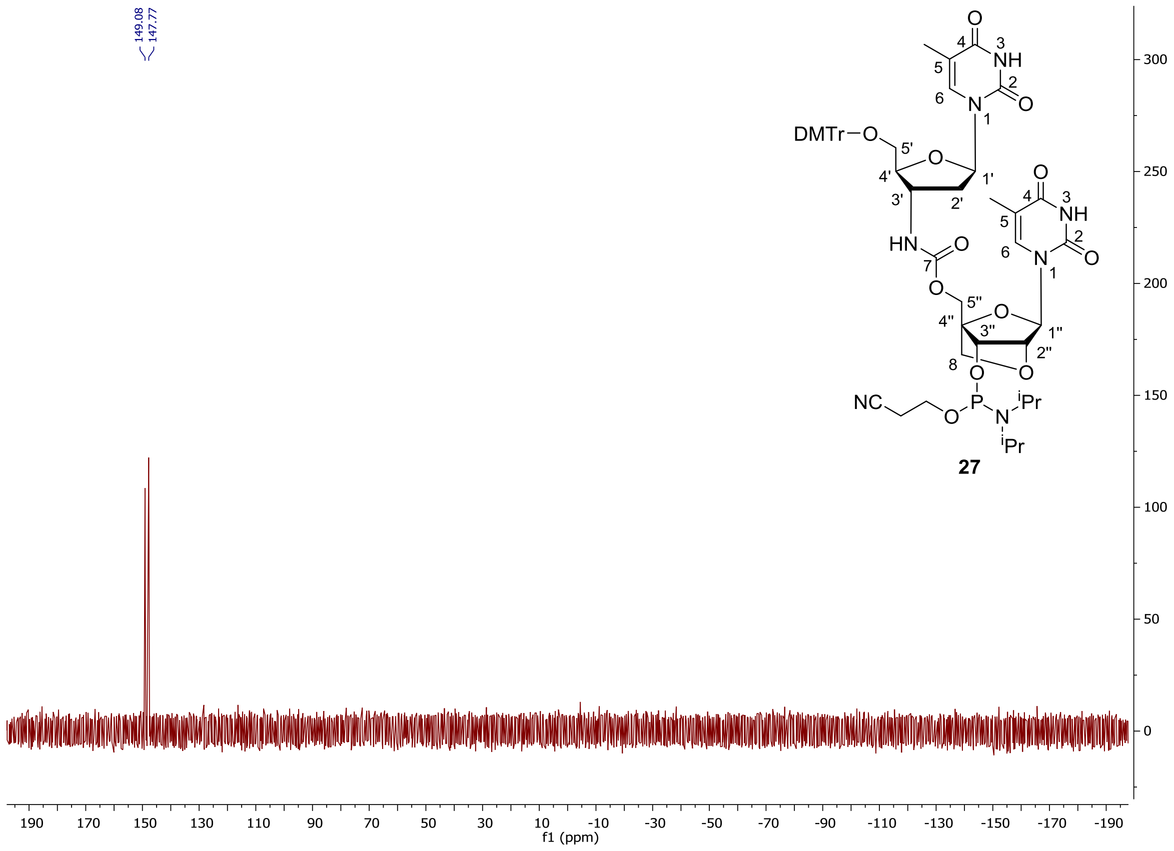

References

1 S. Obika, O. Nakagawa, A. Hiroto, Y. Hari and T. Imanishi, *Chem. Commun.*, 2003, **3**, 2202–2203.

2 A. A. Koshkin, J. Fensholdt, H. M. Pfundheller and C. Lomholt, *J. Org. Chem.*, 2001, **66**, 8504–8512.
